# Genome-wide association study of 12 agronomic traits in peach

**DOI:** 10.1038/ncomms13246

**Published:** 2016-11-08

**Authors:** Ke Cao, Zhengkui Zhou, Qi Wang, Jian Guo, Pei Zhao, Gengrui Zhu, Weichao Fang, Changwen Chen, Xinwei Wang, Xiaoli Wang, Zhixi Tian, Lirong Wang

**Affiliations:** 1 The Key Laboratory of Biology and Genetic Improvement of Horticultural Crops (Fruit Tree Breeding Technology), Ministry of Agriculture, Zhengzhou Fruit Research Institute, Chinese Academy of Agricultural Sciences, Zhengzhou 450009, China; 2 Institute of Animal Science, Chinese Academy of Agricultural Sciences, Beijing 100193, China; 3 State Key Laboratory of Plant Cell and Chromosome Engineering, Institute of Genetics and Developmental Biology, Chinese Academy of Sciences, Beijing 100101, China

## Abstract

Peach (*Prunus persica* L.) is a highly valuable crop species and is recognized by molecular researchers as a model fruit for the Rosaceae family. Using whole-genome sequencing data generated from 129 peach accessions, here we perform a comprehensive genome-wide association study for 12 key agronomic traits. We show that among the 10 qualitative traits investigated, nine exhibit consistent and more precise association signals than previously identified by linkage analysis. For two of the qualitative traits, we describe candidate genes, one potentially involved in cell death and another predicted to encode an auxin-efflux carrier, that are highly associated with fruit shape and non-acidity, respectively. Furthermore, we find that several genomic regions harbouring association signals for fruit weight and soluble solid content overlapped with predicted selective sweeps that occurred during peach domestication and improvement. Our findings contribute to the large-scale characterization of genes controlling agronomic traits in peach.

## Abstract (web summary)

Peach is both an economically important crop species and a model for Rosaceae fruit development research. Here, the authors perform genome-wide association analysis in peach and find candidate genes associated with variation in agronomically important fruit phenotypes.

## Graphical abstract

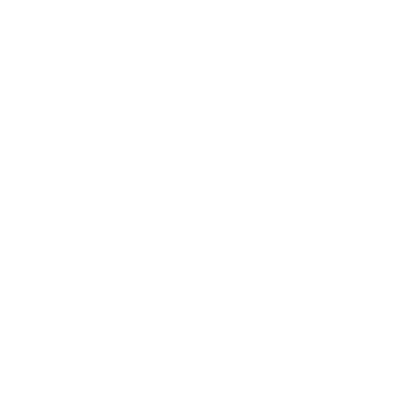

Peach (*Prunus persica* L.) is an economically important deciduous fruit, only exceeded by apple, grape and pear in worldwide production quantity^1^ (FAO^1^). Owing to its small genome and relatively short juvenile period, the peach is considered as a model species for comparative and functional genomic studies of the Rosaceae family^2^. So far, a number of linkage analyses to examine the genetic basis for peach fruit traits have been performed (www.rosaceae.org), but only a few genes were clearly identified as related to qualitative traits such as flesh adhesion^3^, texture^3^ and colour^4^ and fruit hairiness^5^.

Recently, a genome-wide association study (GWAS) was performed using 1,580 peach accessions and genotype data for 5,378 polymorphic SNPs (single-nucleotide polymorphisms) derived from the 9K SNP array developed by the International Peach SNP Consortium^6^. This analysis provided valuable genetic information, but could not precisely determine the candidate genes controlling major agronomic traits in peach due to low coverage of SNPs. An alternative approach, to identify candidate genes is to discover whole-genome-wide SNPs using resequencing technology^7^ and then perform higher resolution GWAS^8^. This approach has been successfully applied to species with short life cycles^9^,^10^,^11^.

Here we present a GWAS for 12 agronomic traits by exploiting natural variation in 129 peach accessions and using high-throughput resequencing technology. Several genomic loci underlying these agronomic traits are identified for the first time. We also find that the linkage disequilibrium (LD) values of peak GWAS signals in peach exhibit different patterns from those reported for annual crops. These findings may help inform peach breeding as well as future sequencing studies and GWAS of peach and other fruit crops.

## Results

### Genotyping of 129 peach accessions

In this study, a total of 129 peach accessions were used for resequencing and subsequent GWAS analysis (Supplementary Tables 1 and 2). Among the 129 accessions, 84 were resequenced as part of our previous study focusing on the evolution and identification of gene regions where domestication had the greatest influence in *P. persica*^7^; 45 accessions were newly added to balance the biased trait segregation (Methods and Supplementary Fig. 1). After resequencing, 121 gb of data were generated, with a mean coverage of ∼89.28% for each accession and an average sequencing depth of ∼4.21 × (Supplementary Table 3). A total of 4,063,377 high-quality SNPs were obtained after SNP mapping and calling (Supplementary Fig. 2). To validate SNP calling results, we randomly selected 35 SNPs and verified them using 105 accessions in the Sequenom MassARRAY platform. We found that the homozygous SNPs were 93.59% accurate and heterozygous SNPs were 83.82% accurate (Supplementary Data 1; Supplementary Table 4). To minimize the effect of the high error rate of heterozygous SNPs in the GWAS analysis, we performed SNP imputation using a genotype likelihood method^12^; although heterozygous SNPs accuracy was not significantly improved, the percentage of untyped (missing) loci decreased abruptly (Supplementary Table 4) after performing this method. Therefore, SNPs imputation data was also used for the following analysis.

To avoid identifying spurious associations^13^, the genetic structure of the 129 accessions was analysed through several methods. Using *Prunus mume*^14^—the closest related species to peach^15^—as an outgroup, the phylogenetic analysis clearly classified the 129 accessions into two divergent groups within the phylogenetic tree (Supplementary Fig. 3a): one corresponding to *P. persica* (cyan, blue and green lines) and another to its closely related wild species (red line). Accordingly, population structure analysis indicated that the LnP(D) (the estimated likelihood values) increased significantly when *K* was increased from 1 to 2 (Supplementary Fig. 3b), suggesting the 129 accessions might be categorized into two populations. Principal component analysis (PCA) (Supplementary Fig. 4) also suggested that the accessions could be separated into two groups. Therefore, accessions were considered as two sub-populations in the following GWAS analysis. Next, we further explored the genetic diversity (Supplementary Tables 5 and 6) among several subgroups (ornamental landraces, edible landraces, and improved varieties) of *P. persica* and their evolutionary status (Supplementary Fig. 3c; Supplementary Note 1).

### Association study of agronomic traits

In this study, three models were adopted and tested: (1) naive model: general linear model (GLM) without any correction for population structure (GLM-no PCA); (2) GLM-PCA model: GLM with PCAs as correction for population structure; (3) MLM model: mixed linear model (MLM) with PCAs and Kinship as correction for population structure. For the 10 qualitative traits, all three models were tested (Supplementary Figs 5–14) whereas for the two quantitative traits, only the MLM model was adopted because previous studies have suggested that it is more reliable than GLM^10^,^16^ (Supplementary Figs 15 and 16). Signals that were repeatedly detected by both GLM-PCA and the other models for qualitative traits were considered as high-confidence GWAS results.

Genetic structure and the type of phenotypic variance of a population can greatly influence the power of GWAS^16^. When the population is selected, a small population size might lead to the detection of significant GWAS signals, particularly for qualitative traits. For instance, in *Arabidopsis*, meaningful GWAS results were obtained from the analysis of only 107 accessions^9^. Our results showed that the detected association signals (Supplementary Table 7) were consistent with previous linkage analyses (Supplementary Table 8), suggesting that the 129 accessions included here were sufficient for identifying real association signals between SNPs and qualitative traits in peach. For example, association peaks related to fruit flesh adhesion and fruit flesh texture were located on chromosome 4, which includes two polygalacturonase genes identified through linkage analysis^3^.

Due to the small population size, it was difficult to determine the leading SNPs within a LD-GWAS region. To investigate the SNPs leading to LD, we performed an additional association analysis on candidate GWAS loci by using a new subset of 345 accessions for six traits (Supplementary Table 9). Flesh adhesion, texture and colour around the stone, kernel taste, fruit weight (FW) and soluble solid content (SSC), were discarded from this additional analysis because: (a) the genes responsible for the first two traits have already been determined^3^; (b) anomalies in the association signals of flesh colour around the stone were found (Supplementary Fig. 17); (c) kernel taste had a severely distorted phenotypic distribution (Supplementary Fig. 1); and (d) little phenotypic information was available for FW and SSC in the additional accessions. We selected three to five representative SNPs around the lead SNP for each of the six qualitative traits (Methods) and performed Sequenom MassARRAY analysis (Supplementary Table 10). Based on these results, the GLM-no PCA and MLM model identified several spurious associations, although the results appeared to be improved by applying the GLM-PCA model because the highest −log_10_ *P* values of the selected SNPs for each trait were all obtained in the GLM-PCA model (Table 1). Additionally, all the GWAS signals from the GLM-PCA model were detected by either or both the GLM-no PCA or the MLM models, further suggesting that GLM-PCA might be the most appropriate model for the examination of qualitative traits in peach. Furthermore, the quantile–quantile plot for the GLM-no PCA model showed the highest deviation from the line of expected *P* values versus observed ones, for traits such as fruit hairiness, flesh colour and flower shape-showyF. The MLM was better than GLM model in this respect but was considered overcorrect, for traits such as flesh adhesion and texture with no significant trait-marker associations. As a result, for qualitative traits, it was expected that controlling population structure would not necessarily yield better results than with the mixed model. However, GLM-PCA, produced a distribution of *P* values comparable to the theoretical one, and could perform better in association mapping for single-gene-controlled traits compared with the other models.

The additional association analysis helped us to determine the lead SNP and significantly increased the association signals, which explained 25.6–91.6% of the phenotypic variance (Table 1). Considering that LD decay was about 20–50 kb for the different subgroups of cultivated peach^7^, we subsequently performed candidate gene searches in the space defined by ±25 kb on either side of the significant association peaks for each agronomic trait. This allowed several candidate genes to be identified that may be responsible for the qualitative traits examined here (Table 1).

### Candidate genes for qualitative traits in peach

**Figure 1:**
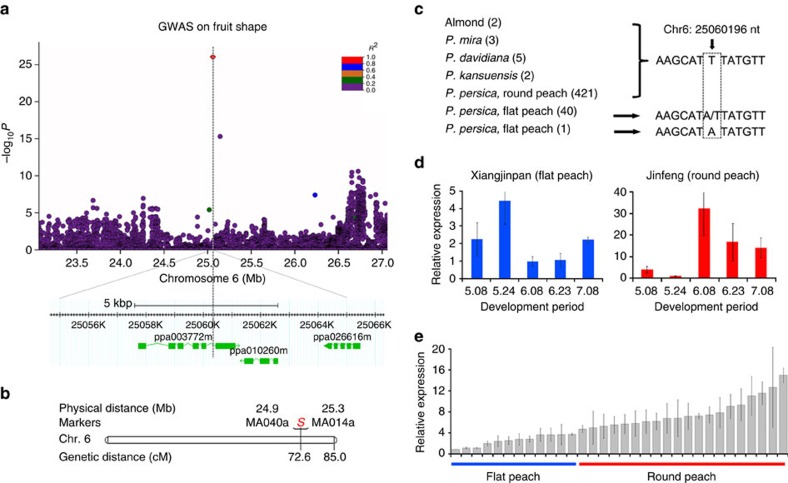
Association results for fruit shape and expression profiles of candidate genes in peach. (**a**) Location of the locus associated to fruit shape in scaffold_6 identified using the GLM-PCA model. The graph plots genomic position (*x* axis) against its significance expressed as −log_10_ *P* value (*y* axis). Genomic position spans ∼2 Mb on either side of the peak SNP, indicated by a black dashed vertical line. The red diamond indicates the associated SNP, and the lighter colours of the remaining markers reflect their successively lower *r*^2^ values (indicated in the legend on the top right). The annotated candidate genes are represented by green boxes displayed below the graph. (**b**) Previous association and linkage analyses as reported in Micheletti^6^ and Dirlewanger^17^, with *S* indicating the gene responsible for fruit shape. (**c**) The mutation found in the fifth intron of gene *ppa003772m* that encodes a constitutively activated cell death 1 protein. Numbers between brackets indicate the sequences analysed within each species/accession. (**d**) Expression patterns of the candidate gene *ppa003772m* in round and flat peach samples, obtained with qRT–PCR, along five developmental periods from May 8 to July 8 at two weeks intervals. (**e**) Relative expression patterns of the candidate gene *ppa003772m* in mature fruits of 32 accessions differing in fruit shape. Values represent mean±s.d. of triplicates for qRT–PCR.

Fruit shape is an important trait affecting peach appearance. We found that its variation was highly associated with an A/T polymorphism detected in the fifth intron of the *CAD1* gene (*ppa003772m*, designated as *PpCAD1*) located on scaffold_6 (25,060,196 bp; Fig. 1a). Notably, *PpCAD1* coding frames did not correspond to the previously annotation of this region (www.rosaceae.org). Cloning of the GDR (Genome Database for Rosaceae)-annotated mRNA sequence in multiple tissues was not achieved, implying that the original annotation was incorrect and that *PpCAD1* might have an additional exon (Supplementary Fig. 18). Both association and expression analyses supported *PpCAD1* as a candidate gene for fruit shape. First, the association signal location correlated with a fruit shape-related quantitative trait locus (QTL)^6^,^17^ (Fig. 1b). Second, the 433 round peach accessions out of the total 474 sampled accessions (474=129+345) exhibited the T/T homozygous allele, while the 41 flat peach accessions exhibited either the A/T heterozygous allele or the A/A homozygous allele (the A genotype is thus dominant; Fig. 1c) of the leading SNP. Third, *PpCAD1* (*ppa003772m*) expression increased earlier in flat peach than in round peach development (Fig. 1d), and high expression was found in round peach at later development stages, indicating the gene was expressed during the phase of fruit development were most growth is due to cell expansion rather than cell proliferation. Fourth, a higher expression was found in mature round peaches (*N*=20) compared with flat peaches (*N*=12) (Fig. 1e). These results provide the first evidence that CAD1 (constitutively activated cell death 1) may be involved in determining fruit shape. Moreover, this SNP (scaffold_6: 25,060,196 bp), which is in an intron, might lead to the observed differences between expression levels and target traits in the association panel. CAD1, a subunit of the highly conserved metabolic switch AMPK (5′-AMP-activated protein kinase) was previously implicated in cell death, pollen germination and pollen tube growth in *Arabidopsis*^18^. Thus, CAD1 may possibly play multiple roles in peach fruit development.

**Figure 2:**
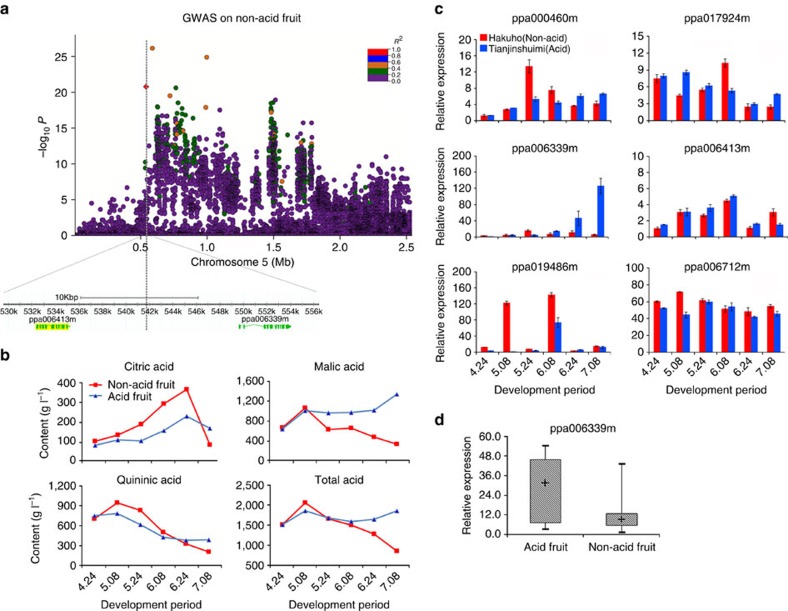
Association results for the non-acid fruit trait and expression profiles of candidate genes in peach. (**a**) Location of the locus associated with the non-acid fruit trait identified on scaffold_5 using the GLM-PCA model. Legends and colour codes for *r*^2^ are the same as described in Fig. 1. (**b**) Variation in citric acid, malic acid, quininic acid and total acid contents during the development of acid and non-acid peach accessions. (**c**) Relative expression of the six genes associated with acid and non-acid peach accessions (indicated in different colours), as quantified by qRT–PCR. (**d**) Relative expression of the candidate gene *ppa006339m* in the mature fruits of nine acid and 15 non-acid accessions. Values represent mean±s.d. of triplicates for qRT–PCR in each sample.

For the non-acidity fruit trait, we determined that the SNP of scaffold_5: 541,075 bp was a leading SNP, as previously reported in a QTL study (scaffold_5: 467,067.. 2,270,122 bp)^6^. The association signal found in the present study was located between two genes, *ppa006413m* and *ppa006339m* (Fig. 2a). We found most of the accessions with the C/T genotype exhibited non-acidity whereas accessions with the T/T genotype exhibited the acidity. Selecting two representative accessions, Hakuho (non-acid with C/T genotype) and Tianjinshuimi (acid with T/T genotype), we evaluated their fruit acid component at different development stages (Fig. 2b) and significant differences were identified. Furthermore, the expression of six flanking genes adjacent to the leading SNPs was examined by quantitative real-time (qRT)–PCR (Fig. 2c). The results showed that *ppa006339m* exhibited significant differences between Hakuho and Tianjinshuimi at almost all developmental stages, particularly at the mature stage whereas no significant differences were found for the other five genes. The expression of *ppa006339m* in the two accessions was positively correlated with changes in total acid and in malic acid content. Furthermore, *ppa006339m* was more highly expressed in the mature stages of acidic fruits (nine accessions) than in non-acidic fruits (15 accessions) (Fig. 2d), further supporting *ppa006339m* as a candidate gene for the control of fruit acidity. In addition, *ppa006339m* encodes a membrane protein annotated as an auxin-efflux carrier. A previous study suggested that a membrane transporter controlled apple acidity^19^. Thus, the mechanisms controlling acidity in peach and apple may both involve membrane transport, although the responsible genes and the transporters they encode may be different.

Another major trait of interest was fruit hairiness; variation in this trait leads to two different fruit types, nectarine and peach. The fruit glabrous trait (nectarine) was recently associated with a recessive transposable element insertion in an *MYB* gene (*ppa026143m*) on scaffold_5, located in the 15,897,836 to 15,899,002 bp interval^5^. The present results revealed that the SNP that was mostly associated to fruit hairiness was located in the 17,576,893 bp of scaffold_5, with a −log_10_ *P* value of 121.92 and a minor allele frequency of 0.22, corresponding to 1.7 Mb distance of *ppa026143m* (Table 1). According to the peach genome annotation results, this SNP resided 343 bp downstream of the stop codon of *ppa010316m*, encoding a pre-mRNA-splicing factor homolog (SPF27). In humans, SPF27 is a ubiquitously expressed nuclear protein, identified as part of the Prp19 complex^20^. Many genes that control trichome initiation and morphogenesis have been identified recently in *Arabidopsis thaliana* and tomato. For example, GLABROUS1 (GL1) belongs to the large family of MYB transcription factors plays a central role in trichome initiation^21^. Both nectarine and the GL1 mutant of *Arabidopsis* lack trichomes. A gene encoding a MYB-like protein (*ppa000733m*) was found among the 13 annotated genes located around the peak association signals for fruit hairiness (±25 kb interval). More research is required to verify if peach genes are indeed contributing to trichome cell elongation.

A single locus (*Y*) controls fruit flesh colour and the gene for white flesh is dominant over that for yellow flesh^22^. However, this trait is occasionally regulated by multiple modifier genes (or duplicate loci) and the markers significantly associated with it were located in linkage group 1 (same as scaffold_1)^23^. Recently, peach flesh colour was characterized using mutant material^4^, and it was revealed that a carotenoid cleavage dioxygenase gene (*ccd4*, scaffold_1: 25,639,445..25,641,500 bp) was responsible for determining flesh colour. The yellow phenotype originated from mutations disrupting *ccd4* function, which prevented carotenoid degradation. In this study, the peak signal in scaffold_1 (24,968,892 bp, Supplementary Fig. 7), which significantly associated with flesh colour, was ∼0.67 Mb away from the *ccd4* gene. However, according to a recent study^6^ the SNP having the strongest association with fruit flesh colour is located behind the *ccd4* gene (scaffold_1: 26,749,525 bp), and relatively distant from the gene. The gene *ppa027093m*, which is involved in the function of flavanone 3-hydroxylase and NAD(P)H-dependent 6'-deoxychalcone synthase, was found in that associated region in our study. Although NAD(P)H-dependent 6'-deoxychalcone synthase plays a role in anthocyanin biosynthesis^24^, the potential participation of these proteins in carotenoid metabolism and their relationship with fruit flesh colour remains unclear.

The presence of anthocyanin pigments is a fruit quality trait that potentially effects on human health. Linkage analysis showed anthocyanin pigments control the flesh colour around the stone trait, which is located in linkage group 3 (ref. 25). Earlier studies also suggested that a hot spot for this trait was located on scaffold_3, because a major transcription factor, *PpMYB10* (regulator of anthocyanin content), was also located on scaffold_3 (12,840,372 to 12,842,225 bp)^26^. A GWAS signal on scaffold_3 also revealed a weak association peak at position 12,902,851 bp, around which two genes encoding MYB transcription factors were found. However, in this study, the SNP most strongly associated with flesh colour around the stone was located on scaffold_6: 2,183,867 bp, scaffold_8: 16,905,885 bp, and scaffold_8: 16,795,565 bp using the GLM-no PCA, GLM-PCA and MLM models, respectively (Supplementary Fig. 10). This observation suggested that, in addition to *PpMYB10*, other modifier genes might be involved in variation in anthocyanin biosynthesis. The weak association near the *PpMYB10* locus might have resulted from the limited sample size or from complex regulation involving numerous genes.

Kernel taste was mapped to linkage group 5 (Table 1) and the association signal peak was close (∼200 kb) to the position deduced from linkage analysis (Supplementary Table 8). The associated SNP explained a high proportion of the phenotypic variance (78.1%) and was located at the promoter region of *ppa015634m*, which encoded the transcription factor bHLH14.

Flower shape-DoubleF has been mapped to linkage group 2 (www.rosaceae.org), with single flowers being the dominant trait. In this study, we found three significant peaks on scaffold_2 and scaffold_6 (Supplementary Fig. 13). Using the additional 345 accessions, the −log_10_ *P* value on scaffold_2 (position: 21,480,531 bp) was only 20.64, which is lower than the value found for other traits (Table 1). Flower shape-ShowyF has been mapped to linkage group 8 (www.rosaceae.org/), where showy flower is the dominant character. In this study, a clear association signal was located at 13,740,117 bp on scaffold_8 (Table 1), with a −log_10_ *P* value of 121.47. This is in agreement with the recent results using SNP arrays (scaffold_8: 13,756,987 bp)^6^. Unfortunately, we were unable to further analyse these candidate genes, as few characterization studies have been performed on kernel taste and on these two flower shape traits.

### Selected regions related to two quantitative traits

FW and SSC are two economically important quantitative traits in peach, but we know little about their genetic basis and the molecular mechanism by which they are controlled. Previous studies revealed that SSC-related QTLs are mainly located in scaffold_1, _2, _4, _5 and _6 (refs 27, 28), while FW-related QTLs are located in scaffold_1 to _7 (ref. 29). In this study, no obvious FW and SSC association signals were found at the genome level (Supplementary Figs 15 and 16), which might have resulted from our small sample size or from the low phentoypic variability observed in the two sub-populations. However, significant FW-associated signals were found in 2007 (*P*<1 × 10^−6^) and in 2010 (*P*<1 × 10^−4^), as well as significant SSC-related loci (*P*<1 × 10^−4^), as listed in Supplementary Table 11. In total, 33 association regions for FW were mapped on each scaffold and 24 association regions for SSC were mapped on each of scaffolds _1 to _8, with the exception of scaffold_5.

FW and SSC are also two frequently considered in peach breeding. To investigate the effects of artificial selection on the loci controlling FW and SSC, the association signals of these two traits (Supplementary Table 11) were compared with domestication and improvement sweeps. A total of 143 potential domestication sweeps (Supplementary Table 12), corresponding to 5% of the assembled genome and ranging from 50 to 650 kb in length (77 kb on average), were identified. Eight large-effect domestication sweep regions longer than 200 kb were distributed on scaffolds_1, _3, _4, _5, _6 and _8. Scaffold_1 had the largest potential domestication region, from 41.05 to 41.70 Mb. Among the 116 potential improvement sweeps identified (Supplementary Table 12), ranging from 50 to 350 kb in length (94 kb on average), 14 large-effect sweeps longer than 200 kb were located on scaffolds_1, _2, _4, _5 and _6.

**Figure 3:**
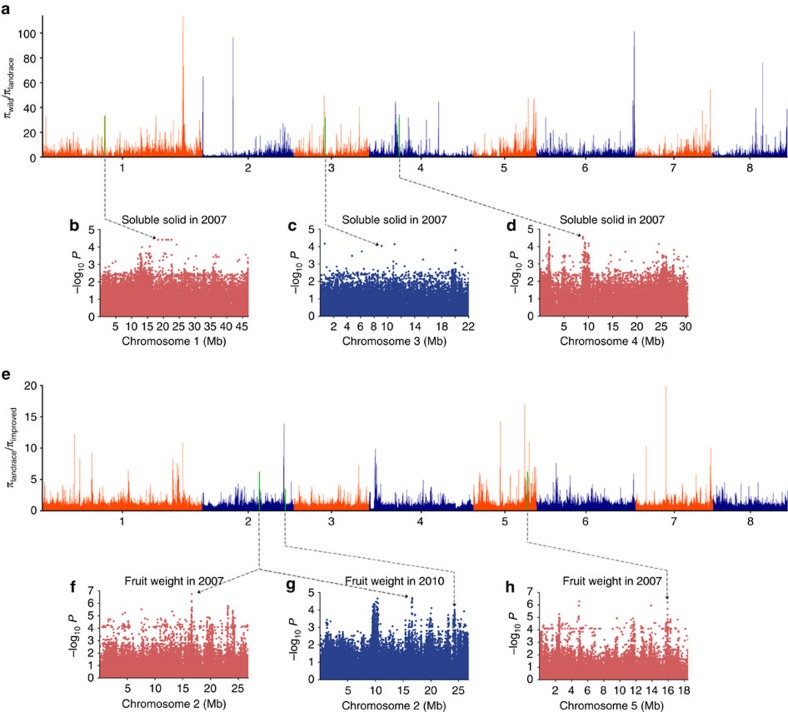
Whole-genome screening for predicted selective sweeps that occurred during peach domestication and improvement. (**a**) Whole-genome screening of the selective sweeps predicted to have occurred during peach domestication, according to the *π*_wild_/*π*_landrace_ values calculated for the eight chromosomes. The selective sweeps that overlapped with characterized GWAS loci are shown in green. (**b**–**d**) The three strong selective sweeps associated with soluble solid content in 2007 in scaffold_1: 18,146,487 bp, scaffold_3: 9,101,540 bp, and scaffold_4: 8,873,368 bp. The *x* axis shows the genomic position, and the *y* axis shows the significance expressed as −log_10_ *P* value. (**e**) Whole-genome screening of the selective sweeps predicted to have occurred during peach improvement, according to the *π*_landrace_/*π*_improved_ values found along the eight chromosomes. The selective sweeps that overlapped with characterized GWAS loci are shown in green. (**f**) The strong selective sweep associated with fruit weight in 2007 in scaffold_2: 16,609,769 bp. (**g**) The two selective sweeps associated with fruit weight in 2010 in scaffold_2: 16,609,769 bp and scaffold_2: 24,271,176 bp, respectively. (**h**) The selective sweep associated with fruit weight in 2007 in scaffold_5: 15,928,967 bp.

Among all identified SSC association signals, three overlapped with domestication sweep regions (scaffold_1: 17.80..18.50 Mb, scaffold_3: 9.10..9.20 Mb, nd scaffold_4: 8.85..8.90 Mb) (Fig. 3a–d), encompassing 60 annotated genes (Supplementary Table 13) that were classified into 11 groups according to gene ontology. However, none of these genes were annotated as being involved in sugar transport or metabolism, and there is little information on their function. The allele frequency of the three lead SNPs in the wild, landrace and improved varieties of peach were also calculated. The most frequent allele of scaffold_3: 9,101,545 bp was observed in 99.09% of landraces, while this allele was only present in 50% of wild peach accessions. However, the most frequent allele at the first and third loci (scaffold_1: 18146487, bp and scaffold_4: 8,873,368 bp) was equally present in landraces and wild peach populations (100% in both). These results suggest that only the second locus was selected during domestication.

In addition to these genes, four FW association signals (three loci) overlapped with the improvement sweep (scaffold_2: 16.60..16.90 Mb, scaffold_2: 24.25..24.30 Mb, and scaffold_5: 15.90..16.05 Mb) (Fig. 3e–h), encompassing 88 genes (Supplementary Table 13) that could be classified into 16 groups according to gene ontology. We focused on the gene sets annotated as ‘primary metabolic process', ‘cellular component organization' and ‘biological regulation'. These gene sets included two genes, *ppa021446m and ppa022308m*, predicted to encode pectinesterase or pectinesterase inhibitor proteins, a set of three repeat genes (*ppa002727m*, *ppa002732m* and *ppa004378m*) predicted to encode β-fructofuranosidase/hydrolase proteins and *ppa021277m*, which is similar to *AtGRF9*, which encodes the growth-regulating factor (GRF) 9 protein in *Arabidopsis*. After calculating the allele frequency of the three lead SNPs in different populations, the percentage of the most frequent allele was 99.09% on the first locus (scaffold_2: 16,609,769 bp) of improved varieties while this value decreased to 83.96% in landraces. The most frequent variant on scaffold_2: 24,271,176 bp was present in 99.09% of improved varieties and 90.57% of landraces. The homozygous genotype of the most frequent allele of the third SNP (scaffold_5: 15,928,967 bp) was more frequent in improved varieties (100.00%) than in landraces (84.91%).

Interestingly, three SSC-associated loci appeared to have experienced selection during domestication, but this does not appear to be the case for FW-associated loci (Fig. 3b–d). However, during peach improvement, four loci associated with FW overlapped with sweeps but this was not the case for loci associated with SSC traits (Fig. 3f–h). This may be because it is more difficult to identify association signals in unstable than in stable traits such as FW^30^. Our results also suggest that some genes related to fruit taste were selected for during domestication but not during improvement. This is also supported by analysis of the distribution of SSC in different peach populations; during peach domestication, from ornamental to edible landraces, the average soluble solid content changed abruptly. However, such marked variation was not found during improvement from edible landraces (11.39%) to improved variety groups (11.32%). Moreover, the titratable acid content, an important component of flavour, decreased during improvement (Supplementary Table 14), and fruit volatiles such as C6 compounds, aldehydes and alcohols decreased from wild peaches to landraces to improved varieties^31^.

## Discussion

Association mapping has identified QTL in annual crops, such as *Arabidopsis*^9^, rice^10^, foxtail millet^11^ and maize^32^. In the present analysis, peak association signals for fruit hairiness, fruit shape, flesh adhesion, flesh texture and non-acidity were located close to the candidate genes identified by linkage analysis. Some candidate genes for flesh adhesion and flesh texture that were previously identified by linkage analysis, were also identified by GWAS. Furthermore, two candidate genes for fruit shape (*ppa003772m*) and non-acid fruit (*ppa006339m*) were identified. These results reveal that GWAS can be employed to find candidate genes for specific qualitative traits in peach, even using relatively few accessions. However, this may have only been possible in peach due to its low rate of polymorphism, resulting from self-pollination and grafting^7^. On the other hand, results for fruit hairiness and fruit flesh colour were not consistent with the results of gene identification obtained through reverse genetics^4^,^5^. This inconsistency might be due to fruit hairiness and fruit flesh colour being determined by the insertion of transposable elements^4^,^5^, a type of variation difficult to screen using next-generation sequencing with low sequence depth, and that cannot be identified by GWAS, using SNPs only.

The discovery of selective sweeps related to domestication and improvement can also aid in candidate gene identification, as was the case in recent studies reporting causative variants for pink colouration in tomato^33^ and bitterness in cucumber^34^. Overlap between associated loci and selective sweeps may suggest that the genes underlying these traits are under selection. In the present study, three gene sets were found in the regions overlapping FW association signals and improvement sweeps. Proteins predicted to be encoded by those genes (pectinesterase and β-fructofuranosidase) have been reported as cell wall-bound enzymes, the activity of which increases steadily during fruit maturation in tomato^35^. They are considered to be a component of cell wall loosening and involved in developmental events where cell wall modification occurs^36^. We speculate that these enzymes may increase fruit weight by transporting more sugar into parenchyma cells, which results in greater water absorption and enlarged cells. One gene, *ppa021277m*, is annotated as encoding a GRF protein. GRF proteins and their effects on cell proliferation have been studied intensively in rice^37^ and *Arabidopsis*^38^,^39^. In *Arabidopsis*, most *GRF* genes are strongly expressed in actively growing and developing tissues. Overexpression of *AtGRF1* (ref. 38), *AtGRF2* (ref. 38) and *AtGRF5* (ref. 39) results in larger leaves whereas triple null mutants of *AtGRF1*–*AtGRF3* or single mutants of *AtGRF5* had smaller leaves due to decreased in cell number. However, whether *ppa021277m* is involved in fruit enlargement is unknown.

Self-fertilization in peach favours the maintenance of extended LD^40^. However, peach landraces showed low levels of LD^41^, suggesting a conflict between maintaining LD levels and LD decay. In this study, lower levels of LD (*r*^2^) were found for the peak signals of fruit shape (Fig. 1a), non-acid fruit (Fig. 2a), and eight other qualitative traits with their surrounding SNPs when compared with their genome-wide LD levels (Supplementary Fig. 19). We also examined this aspect for other unidentified loci associated with important characters. We calculated the values of *r*^2^ and *D*′ of peach in contiguous sliding windows (Supplementary Fig. 20), and found a normal distribution for *r*^2^ across the total peach genome (Supplementary Fig. 21a). However, the performance of *r*^2^ across some chromosome regions under domestication and improvement selection showed a discrete distribution (Supplementary Fig. 21b,c), although the *r*^2^ distribution in improvement regions showed an opposite trend to that of domestication regions, in which *r*^2^ tended to be lower than the average value found for the total genome. These differences might be due to differences in breeding methods of the accessions used to identify domestication or improvement regions. For example, self-fertilized landraces were mainly selected from seedlings and propagated using grafting over a long period, whereas improved varieties were selected from population crosses, and used in experiments in a short period.

To determine the reason for the lower *r*^2^ of domestication regions compared with peach's total genome, we hypothesized that a mutation resulting in a new character and being under domestication would ultimately be fixed through propagation. However, this process might not happen in plants propagated by sexual reproduction. It is known that both *r*^2^ and *D*′ measure recombination history, but *r*^2^ also summarizes mutational history^42^. Thus, the difference between *r*^2^ and *D*′ might reflect the effect of mutational loci in LD. This effect was compared between soybean, a self-fertile crop that is propagated by sexual reproduction^43^, and peach which has a fertilization similar to soybean but a different propagation mode. Results showed that *r*^2^ values were lower than *D*′ in most sliding windows of soybean (Supplementary Fig. 22a). In peach, *r*^2^ values were considerably lower than *D*′ in a few special genome regions (Supplementary Fig. 22b), suggesting a link between grafting and decreased LD. This might have resulted from selecting offspring from bud mutations that were maintained through vegetative propagation^44^ during domestication, leading to the fixation of those mutations in the absence of recombination. It is well-known that peach mutations are very common, and therefore vegetative propagation has been one of the main methods of peach breeding (Supplementary Fig. 23). This hypothesis was supported by most regions with high *D*′/*r*^2^ (0.5% of 4370 blocks with 50 kb length) being related to domestication regions (Supplementary Table 15; red dots in Supplementary Fig. 22b). In rice, a narrower GWAS interval was also obtained for wild rice populations with short recombination history than for cultivated rice populations^45^. Therefore, candidate regions for peach should be regarded cautiously as this species exhibited reduced recombination and extended LD in the whole genome but quick decay in particular regions usually containing candidate causal genes related to important traits.

This phenomenon has serious consequences. For example, considering the LD decay of edible peach (14 kb)^7^, about 16,042 SNPs will cover the total peach genome (224.6 Mb)^46^. However, domestication regions exhibited faster decay than total genome and more SNPs need to be identified in the domestication regions containing key genes to carry out association analysis. Therefore, the present study used deep sequencing to perform GWAS to screen for SNPs in regions with low LD levels^13^,^47^ because there is a positive correlation between sequencing depth and the ratio of called SNPs to whole genotype (Supplementary Fig. 3 in Cao *et al*.^7^). Additionally, deeper sequencing may be needed in future studies to identify the genes underlying phenotypic variation caused by structural variation (such as transposable elements insertion), similarly to that performed for fruit flesh colour^4^ and fruit hairiness^5^ characteristics. This statement differs from that in early studies, which suggested that low-coverage sequencing might be powerful enough for GWAS in self-pollinated species^48^.

The present findings help clarify the genetic basis for major agronomic traits in peach, potentially allowing for more efficient breeding. Future GWAS analysis, in conjunction with deep sequencing, can be expected to further examine the genetic basis for agronomic traits in peach given it high resolution despite the low level of LD.

## Methods

### Plant materials

We used 129 peach accessions: 12 wild accessions, 62 landraces and 55 ancient and modern improved varieties (Supplementary Table 1). Among them, only 14 cultivars were bred and released in the United States, Japan, Canada and Italy, while the remaining were obtained from China. To ensure the representativeness of the selected samples, except for the several wild-related species of peach, different known geographic subgroups of *P. persica* were included. The well-known breeding parents were also present in the improved varieties groups, such as Shu Guang, Jing Yu and Zhong You Tao 4# of Chinese accessions, as well as in the ones outside China, Okubo, Bailey, May Fire and Elberta. All plant materials were collected and maintained at the National Fruit Tree Germplasm Repository, Zhengzhou Fruit Research Institute, Chinese Academy of Agricultural Sciences, in China. During the growing period, all cultivars were equally managed. Eighty-four accessions had been previously introduced^7^; to increase the proportion of improved varieties, for studying the improving event, and to keep a agronomic segregation balance of 10 targeted traits, the number of improved varieties under study was increased. Whereas in a previous study, 23 out of 84 accessions corresponded to improved varieties, in the present study, 36 out of 45 accessions were improved varieties. The ratio of dominant to recessive characters in the 10 qualitative traits, namely fruit hairiness, fruit shape, fruit flesh colour, flesh adhesion, flesh texture, flesh colour around the stone, non-acid fruit, flower shape-DoubleF, flower shape-ShowyF and kernel taste, was also different from the one used in a previous study: 5.46, 0.08, 4.25, 1.86, 5.92, 1.25, 0.93, 6.64, 15.60 and 26.67 in the previous study versus 1.47, 0.27, 1.16, 1.27, 6.88, 1.06, 1.05, 13.00, 1.35 and 19.50 in the present study. This suggested all traits tended to have a balanced segregation, with the exception of flesh adhesion, flesh texture and flower shape-DoubleF.

### Agronomic evaluation

Agronomic traits under investigation for association analysis were divided into three categories: fruit characteristics (fruit hairiness, fruit shape, fruit flesh colour, flesh adhesion, flesh texture, flesh colour around the stone, non-acid fruit, FW and SSC), seed characteristics (kernel taste), and flower characteristics (flower shape-DoubleF and flower shape-ShowyF). The fruit and seed characteristics were evaluated using fully matured fruits. All qualitative traits were analysed in at least five fruits or flowers in 2010, which were randomly picked from two trees for each accession. Some traits, such as fruit flesh colour, flesh adhesion, flesh texture, flesh colour around the stone and non-acid fruits were verified in two successive years, from 2011 to 2012. All agronomic traits considered here were characterized based on previously published plant genetic resources' evaluation criteria^49^. Although fruit non-acidity has been regarded as a quantitative trait^50^, in the present study it was evaluated as a qualitative trait according to Dirlewanger^17^, meaning that fruits were considered to have low-acidity if the pH was >4. When pH and taste were not correlated, the titratable acid content of the fruit, which was measured in 2014, was also taken into account. FW was determined as the average value of 10 fruits collected from two trees in 2007 and in 2010. SSC was determined with a hand refractometer in 2007, 2010 and 2014, using a mix of the juice of those 10 fruits prepared in the laboratory, and the SSC determined in the first two years were used in association analysis. Supplementary Fig. 1 shows the frequency distribution of all listed traits (Supplementary Tables 1 and 2) across the 129 peach accessions.

In addition, the acid content was regarded as a quantitative character when analysing its changes during the fruit development period. Hakuho (non-acid) and Tianjinshuimi peaches were picked in the field, during fruit development period, from April 24 to July 8, 2014 representing 30, 45, 60, 75, 90 and 105 days after full bloom and brought to the laboratory. The mesocarp was immediately removed and placed in liquid nitrogen; after that, the flesh was frozen at −80 °C until analysis. Samples were homogenized in 80% ethanol and centrifuged at 12,000*g* for 15 min. The resulting supernatant was filtered through a 0.22-μm mesh before being subjected to an ion chromatography (DX-500, Dionex, American) analysis. The different acids were detected using a Dionex ED-40 detector and an Inertsil AS-11 column (250 mm × 4.6 mm i.d., 5 μm particle size). The column was maintained at 35 °C. Samples were eluted with 0.022 mol l^−1^ KH_2_PO_4_ solution with pH 2.4 and injected at a flow rate of 0.8 ml min^−1^. Eluted compounds were detected by UV absorbance at 210 nm. All samples were prepared in triplicate. Citric acid, malic acid and quininic acid were identified via comparisons of their retention times and UV spectra with external standards. Acid concentrations were calculated from calibration curves obtained from the corresponding external standards and expressed in g l^−1^.

### DNA preparation and sequencing

DNA was extracted from the fresh leaves of the added 45 peach accessions using the CTAB (hexadecyl trimethyl ammonium bromide) method^51^. The library insert size was 500 bp and the pair-end reads were 49 bp. All libraries underwent high-throughput sequencing using Illumina GA2.

### SNP calling

To minimize the negative influence of sequencing errors in gene identification, two SNP-calling software packages were used simultaneously. SNP detection was performed using SAMtools^52^ and the Genome Analysis Toolkit (GATK, version 2.4-7-g5e89f01)^53^. Only the SNPs detected by both methods were further analysed. The detailed processes were as follows: (1) After Burrows-Wheeler Alignment Tool (BWA) alignment, the reads around indels were realigned. Realignment was performed with GATK in two steps. The first step used the RealignerTargetCreator package to identify regions where realignment was needed, and the second step used IndelRealigner to realign the regions found in the first step, which produced a realigned BAM (sequence alignment/map format) file for each accession. (2) SNPs were called at the population level with GATK and SAMtools. For GATK, the SNP confidence score was set as greater than 30, and the parameter -stand_call_conf was set as 30; a total of 7,559,771 SNPs was reported. The same realigned BAM files were used in SNP calling through the SAMtools mpileup package, and a total of 5,509,663 SNPs was reported. (3) In the filter step, the sites commonly identified by both GATK and SAMtools were selected using the SelectVariants package; SNPs with allele frequencies lower than 1% in the population were discarded. In the end, a total of 4,063,377 SNPs were retained.

### Population genetics analysis

Using the neighbour-joining method and a distance matrix calculated in PHYLIP 3.68 (ref. 54), a phylogenetic tree was constructed and displayed in MEGA 5.0 (ref. 55). Using all SNPs, we evaluated the population structure of the 129 peach accessions in Structure 2.3.4 (ref. 56). The input parameter *K* in Structure software ranged from 2 to 8, representing the simulated number of groups in ancient populations. Population genotypes were also transformed into a matrix containing 0, 1 and 2. The homozygous and heterozygous genotypes aligning with the reference genome were indicated with 0 and 1, respectively, while a homozygous genotype for the non-reference was indicated with 2. Sample covariance was then calculated from this matrix containing information for all individuals' information. A PCA of whole-genome SNPs was performed with the EIGENSOFT 4.2 software^57^. The eigenvector decomposition of the matrix was calculated in R using the reigen function, and the PCA was plotted using those values.

### Genotype imputation

The genotyping error associated to heterozygotes was inevitable given the low sequencing depth (5 × ). Therefore, we treated the loci which GQ (genotype quality)≤3 as missing in the raw VCF file. For imputation, we used the function infer sporadic missing genotype data of Beagle software^58^ and the following default parameter settings: unphased and non-reference; 15 iterations for imputation. Imputation was performed using a sliding window of 50,000 SNPs in the sequence data. No pedigree information was used in the imputation procedure.

### Genome-wide association study

In these study, three models (run in TASSEL^59^) were used in the association analysis based on the resequencing data of 129 peach accessions: the first used a GLM without any consideration for PCA (GLM-no PCA); the second also used GLM but took PCA results into account as the fixed effect (GLM-PCA); and the third was a MLM using PCA results and the Kinship value (the random effect based on the genetic relatedness across all accessions), as it considered population structure and cryptic relationships thereby minimizing false positives and increasing the statistical power. Results were compared with determine the best model for our analysis. Before the analysis, the SNPs with deficiency rates above 20% in all accessions were filtered out and the SNPs with minor allele frequency lower than 0.05 were conserved, albeit with consideration for the fact that they had a severe distorted trait. We defined a whole-genome significance cutoff from the Bonferroni test threshold (1 × 10^−8^). In the association analysis of the additional 345 accessions, only GLM-no PCA was used to obtain final results. The specific commands to run each model were as follows: (1) GLM-no PCA:./tassel4.0_standalone/run_pipeline.pl -fork1 -h./genotype.txt -fork2 -r./phenotype.txt -combine4 -input1 -input2 -intersect -glm -export glm_output -runfork1 -runfork2; (2) GLM-PCA:./tassel4.0_standalone/run_pipeline.pl -fork1 -h./genotype.txt -fork2 -r./phenotype.txt -fork3 -q./PCA.cov -combine5 -input1 -input2 -input3 -intersect -glm -export glm_output -runfork1 -runfork2 -runfork3; (3) MLM:./tassel4.0_standalone/run_pipeline.pl -fork1 -h./genotype.txt -fork2 -r./phenotype.txt -fork3 -q./PCA.cov -fork4 -k./kinship.txt -combine5 -input1 -input2 -input3 -intersect -combine6 -input5 -input4 -mlm -mlmVarCompEst P3D -mlmCompressionLevel None -export mlm_output -runfork1 -runfork2 -runfork3 -runfork4.

### Genome scanning for selection

Under domestication sweeps, genetic regions in landraces of *P. persica* should have significantly lower diversity than in wild peach, including *Prunus tangutica*, *Prunus communis*, *Prunus mira*, *Prunus kansuensis* and *Prunus davidiana*. Under improvement sweeps, improved varieties should have lower diversity than landraces. Thus, the genome was scanned in 50 kb sliding windows, with a step size of 5 kb, to identify the regions with significantly low levels of polymorphisms in each group, based on genetic diversity ratios (*π*; wild to domesticated accessions and domesticated to improved varieties). The value of *π* was calculated on the basis of the genotypes of each line at the SNP positions using BioPerl. Windows in the top 5% of *π* values were considered candidate regions for domestication and improvement sweeps.

### LD analysis

To evaluate the effects of mutation on LD, we calculated the *r*^2^ of several non-overlapping 50 kb sliding windows across the peach genome, using PLINK^60^. The *D*′ value in a random chromosome was also measured to compare *r*^2^ and *D*′ distributions across the genome.

### Expression analysis using qRT–PCR

Using an extraction kit (Aidlab, Beijing, China), RNA was isolated from the fruit flesh of the following three groups: the first comprised two accessions with different fruit shapes (Xiangjinpan: flat peach; Jinfeng: round peach, Fig. 1d), which were examined in different developmental stages; the second comprised 32 accessions of round and flat peach (Fig. 1e) and 24 accessions of acid and non-acid mature peach (Fig. 2d) at the mature stage; the third comprised two accessions with different acid contents harvested at six developmental stages (Fig. 2b). After extraction, first-strand cDNA synthesis was carried out using 2 μg RNA and the Transcriptor First Strand cDNA synthesis kit (Takara, Dalian, China), according to the manufacturer's protocol. Candidate gene sequences of flat peach (*ppa003772m*) and non-acid fruit (*ppa006413m* and *ppa006339m*) traits were downloaded from the Genome Database for Rosaceae (GDR; www.rosaceae.org). Gene-specific primers were designed using Primer-BLAST software (National Center for Biotechnology Information, Maryland, USA). We performed qRT–PCR using the LightCycler System (Roche LightCycler 480; Roche Diagnostics), following the manufacturer's protocol. Relative expression levels were estimated by the 2^−ΔΔCT^ method.

### Data availability

All raw sequences of the 129 peach accessions have been deposited in the Sequence Read Archive (SRA) of National Center for Biotechnology Information with accession codes SRA073649 and SRA261655. The SNPs have also been deposited into Figshare database (https://figshare.com/articles/Peach_SNP_database/3486737). All other relevant data is contained within the paper and its Supplementary Files or are available from the corresponding author on request.

## Additional information

**How to cite this article:** Cao, K. *et al*. Genome-wide association study of 12 agronomic traits in peach. *Nat. Commun.* **7,** 13246 doi: 10.1038/ncomms13246 (2016).

**Publisher's note:** Springer Nature remains neutral with regard to jurisdictional claims in published maps and institutional affiliations.

## Supplementary Material

Supplementary Information Supplementary Figures 1 - 24, Supplementary Tables 1 - 16, Supplementary Note 1 and Supplementary References

Supplementary Data 1 The SNP validation by MassArray sequenome technology.

## Figures and Tables

**Table 1:** Lead SNPs significantly associated with 10 agronomic traits according to the genome-wide association study performed for the 129 peach accessions and related candidate genes.

**Trait**	**Scaffold**	**Lead SNP**	**Major allele**	**Minor allele**	**minor allele frequency**	**−Log**_**10**_ ***P*** value*	**−Log**_**10**_ ***P*** **value**†	**Explained variation (%)**	**Candidate genes**	**Annotation**
Flesh adhesion	4	22,695,515^b,c^	A	C	0.31	10.20		30.9	*ppa006857m*	Polygalacturonase
Flesh texture	4	22,695,495^a,b^	C	T	0.49	14.22		36.5	*ppa006857m*	Polygalacturonase
Flesh colour around stone	7	12,901,845^a,b^	G	T	0.18	6,89		22.6	—	—
Kernel taste	5	12,622,852^b,c^	T	A	0.03	40.93		78.1	*ppa015634m*	Transcription factor bHLH14
Fruit hairiness	5	17,576,893^a,b^	A	G	0.22	27.11	121.92	80.6	*ppa010316m*	Pre-mRNA-splicing factor SPF27 homolog
Fruit flesh colour	1	24,968,892^b,c^	C	A	0.47	13.72	25.43	30.2	*ppa027093m*	Flavonol synthase/flavanone 3-hydroxylase
Fruit shape	6	25,060,196^a,b,c^	A	T	0.04	26.04	182.72	91.6	*ppa003772m*	CAD1 (constitutively activated cell death 1)
Non-acid fruit	5	541,075^b^	T	C	0.21	20.77	89.42	71.3	*ppa006339m*	Auxin efflux carrier family protein
Flower shape-doubleF	2	21,480,531^a,b^	A	G	0.09	46.55	20.64	25.6	*ppb023753m*	Cyclic nucleotide-gated ion channel 1
Flower shape-ShowyF	8	13,740,117^a,b,c^	A	C	0.15	23.86	121.47	82.0	*ppa016980m*	Predicted protein

The letters ‘a', ‘b' and ‘c', present in the column ‘lead SNP', indicate that the locus was identified in an association study using GLM-no PCA, GLM-PCA and MLM models, respectively.

^*^The association values resulting from resequencing 129 accessions. Among them, the *P* value of the association signals of flesh adhesion and flesh texture were selected from the results derived from the GLM-PCA model, since the GLM-PCA and MLM models presented the same result.

^†^The *P* value of the associations resulting from the Sequenom MassARRAY analysis of 345 accessions.
