# Supplementary material for: Genome-wide association study of 12 agronomic traits in peach
Source: Nat Commun. 2016 Nov 8;7:13246. doi: 10.1038/ncomms13246 (PMC5105138; doi:10.1038/ncomms13246)
Supplement: Supplementary Information — Supplementary Figures 1 - 24, Supplementary Tables 1 - 16, Supplementary Note 1 and Supplementary References [file ncomms13246-s1.pdf]

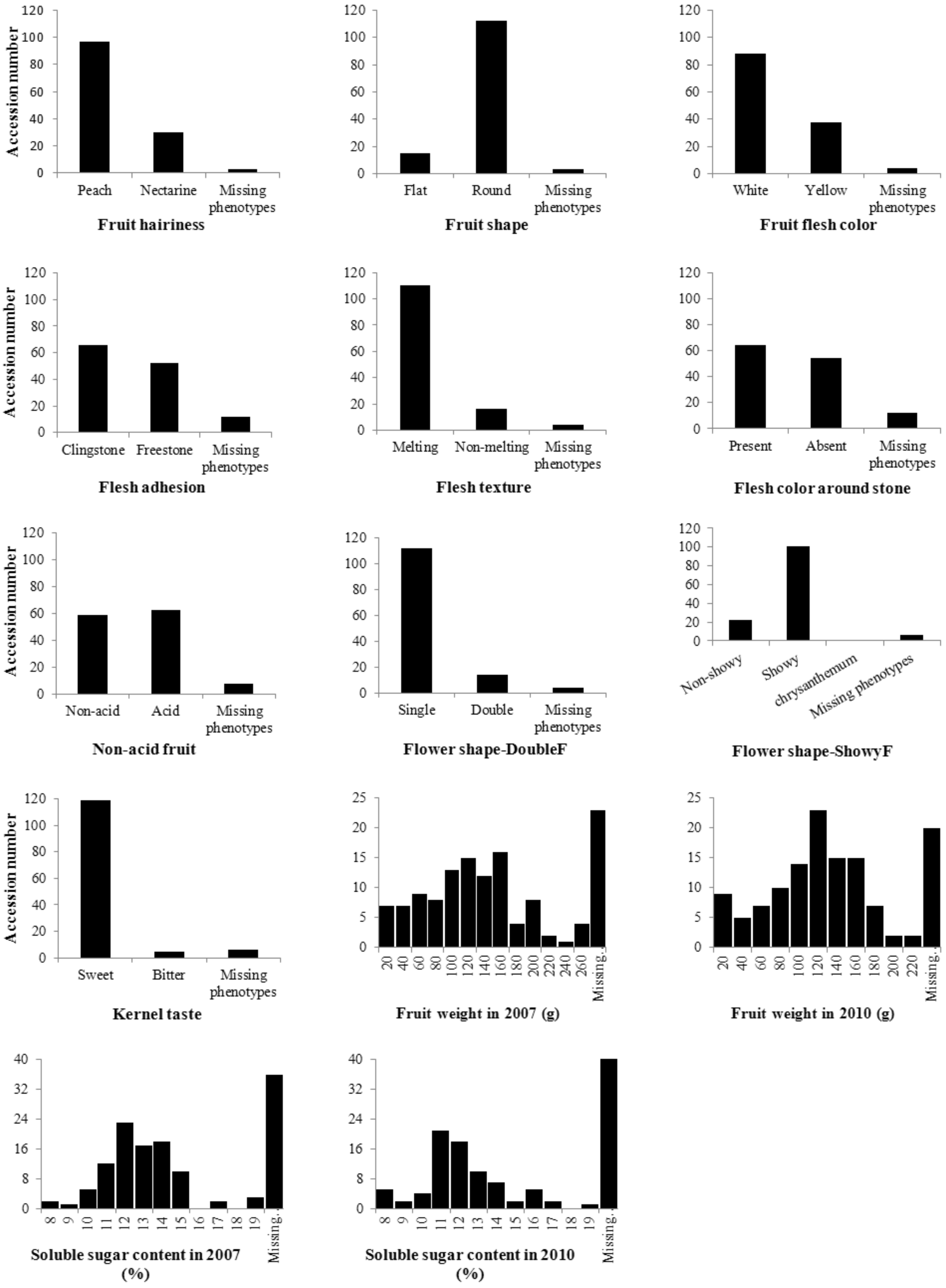

Supplementary Figure 1: Frequency distribution of the variation of 12 traits across the 129 samples used in the resequencing analysis.

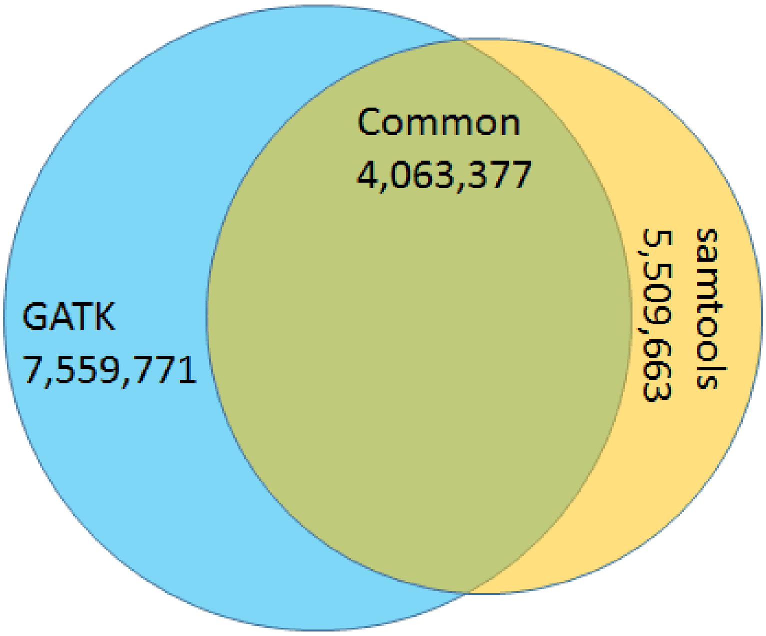

Supplementary Figure 2: Venn diagram showing the number of unique and common SNPs which called by the GATK and SAMtools software packages. The identification of the SNPs associated with variable characters was the main objective of this study, differing from a previous paper focusing on peach evolution[7]. Therefore, two software packages, GATK and SAMtools, were used to detect SNPs and those detected by both packages (common SNPs), which were classified as reliable SNPs, were further analysed (Methods).

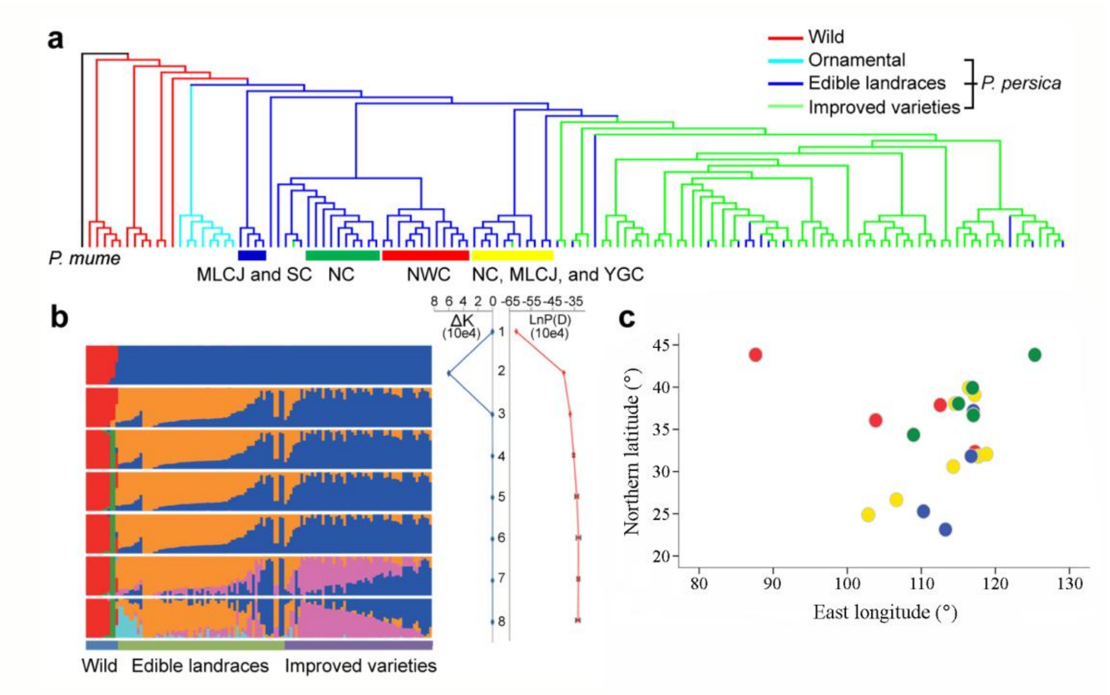

Supplementary Figure 3: Peach domestication history. (a) Rooted phylogenetic tree of the 129 peach accessions considered in this study, based on 4.1 million SNPs and using Prunus mume as the outgroup. (b) Population structure of the 129 peach accessions obtained in FRAPPE analysis. (c) The coordinates of different geographic distribution of the edible landraces subpopulations. In the Supplementary Fig. 3a, the four divergent groups, wild, ornamental, edible landraces, and improved varieties, are coloured in red, cyan, blue, and green, respectively. The description of all accession derived from two Supplementary Table 1 in this and previous study[7]. The horizontal bars in blue, green, red, and yellow indicate different subgroups within the edible landraces group. The geographic origin of each subgroup is list beneath the bars: Northwest China (NWC), YunGui plateau (YGC), Northern China (NC), the middle and lower reaches of the Changjiang River (MLCJ), and Southern China (SC). In the Supplementary Fig. 3b, Different colors represent different populations according to K (the number of populations) value analysis. In the Supplementary Fig. 3c, colors are the same as in the horizontal bars of geographic subgroups present under the phylogenetic tree. Moreover, the coordinates indicate the longitude and latitude of provincial capital where landraces originated.

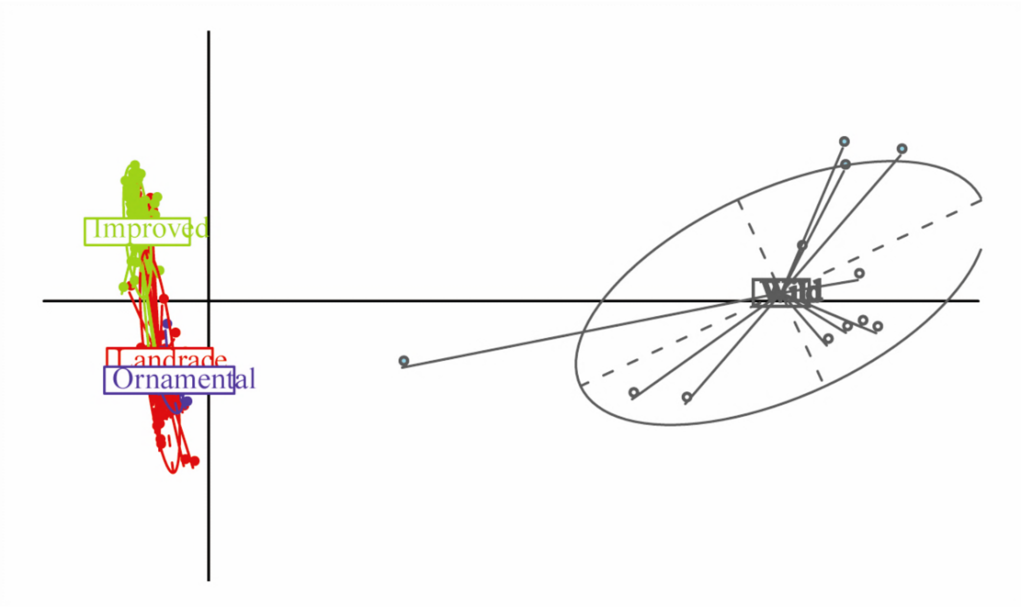

Supplementary Figure 4: Principal component analysis of the wild, ornamental, edible landraces, and improved peach varieties considered in the present study. All identified common SNPs were used in the principle components analysis. Each peach accession is represented by a point in the two-dimensional space defined by the eigenvectors of the first and second principal components. Different colours correspond to different peach groups: wild peach is indicated in black, edible landrace in red, ornamental in blue, and improved varieties in green. Samples distribution demonstrates that all cultivated peach varieties are genetically distant from wild peach.

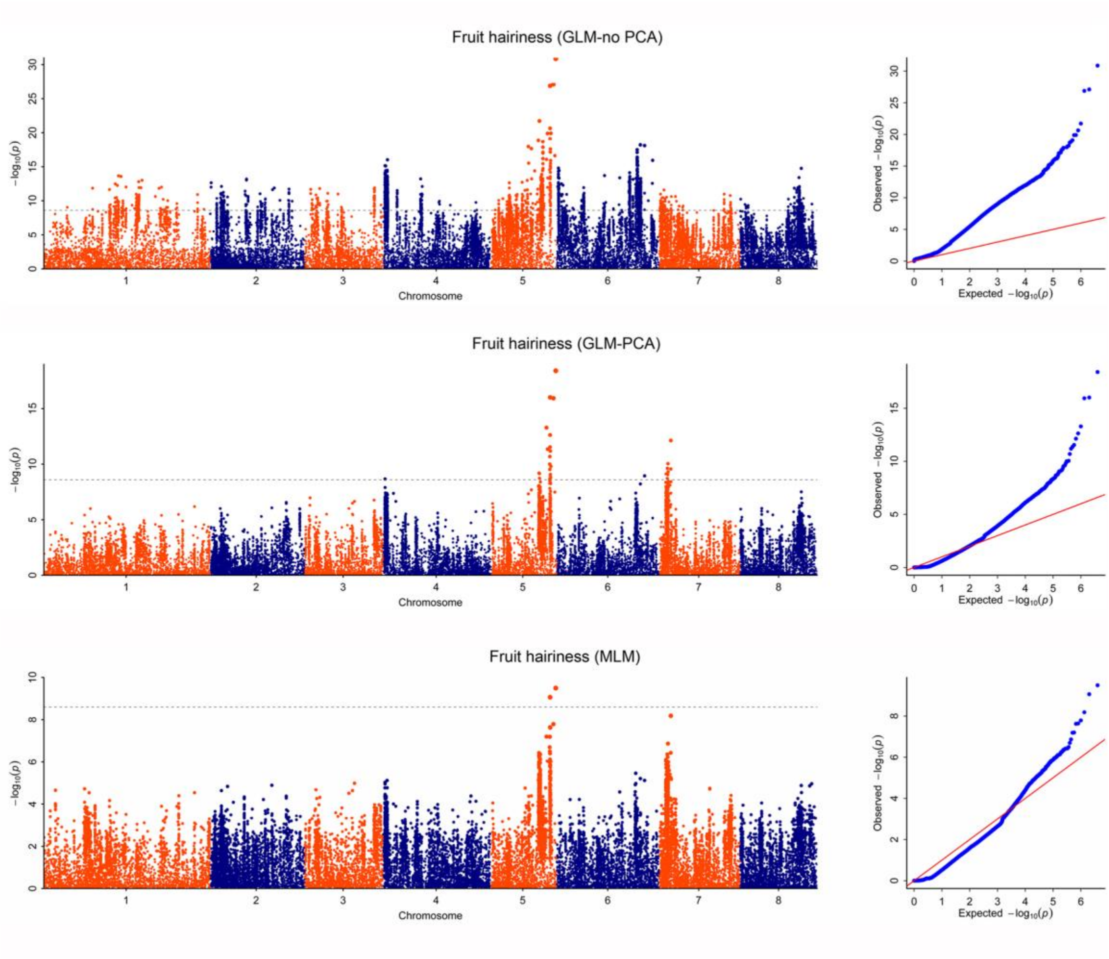

Supplementary Figure 5: Manhattan and quantile-quantile plots for Genome-wide association studies of fruit hairiness trait in 129 peach accessions using three analysis models. Negative $\log_{10}$-transformed P values from the genome-wide scan were plotted against SNPs position on each of the eight chromosomes. The dotted horizontal line indicates the genome-wide significance threshold ($P = 1.0 \times 10^{-8}$). The description of following figures were same as above.

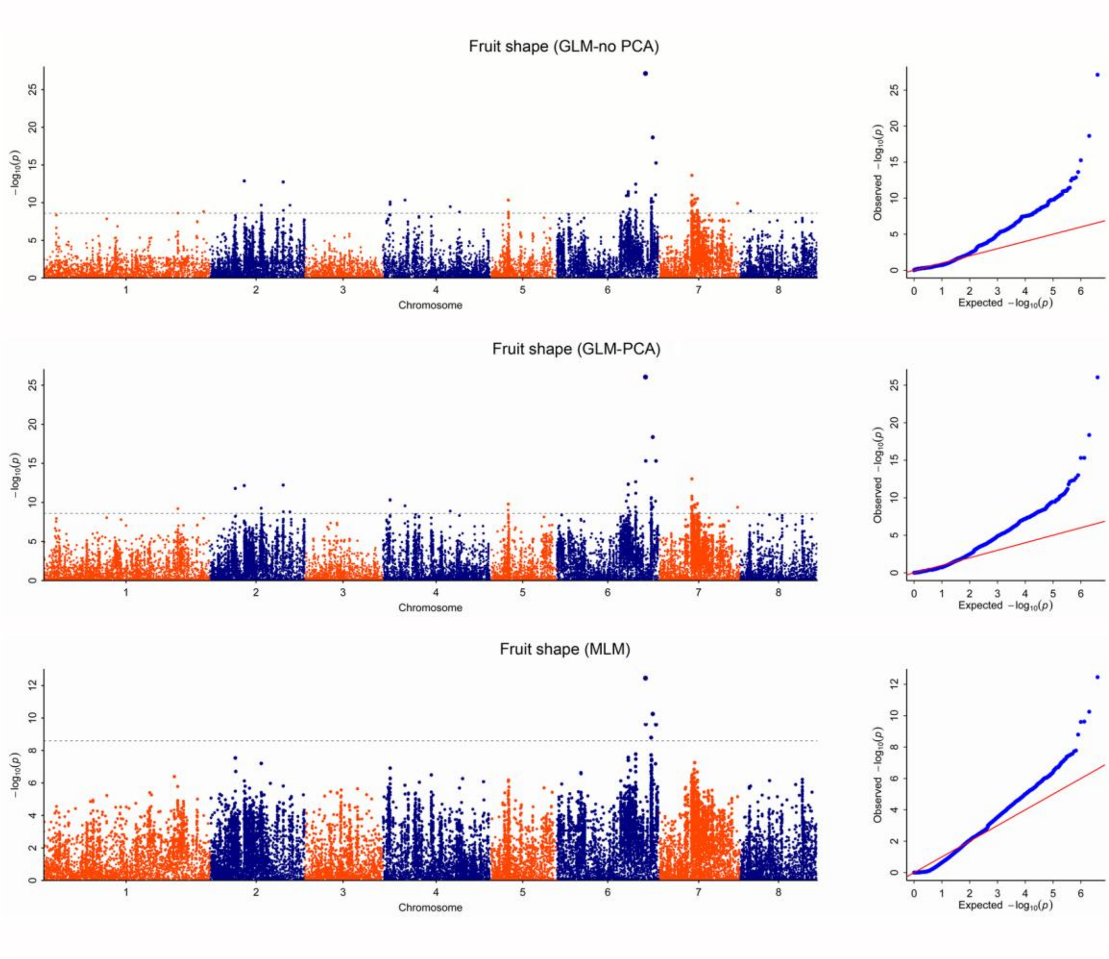

Supplementary Figure 6: Manhattan and quantile-quantile plots for Genome-wide association studies of fruit shape trait in 129 peach accessions using three analysis models.

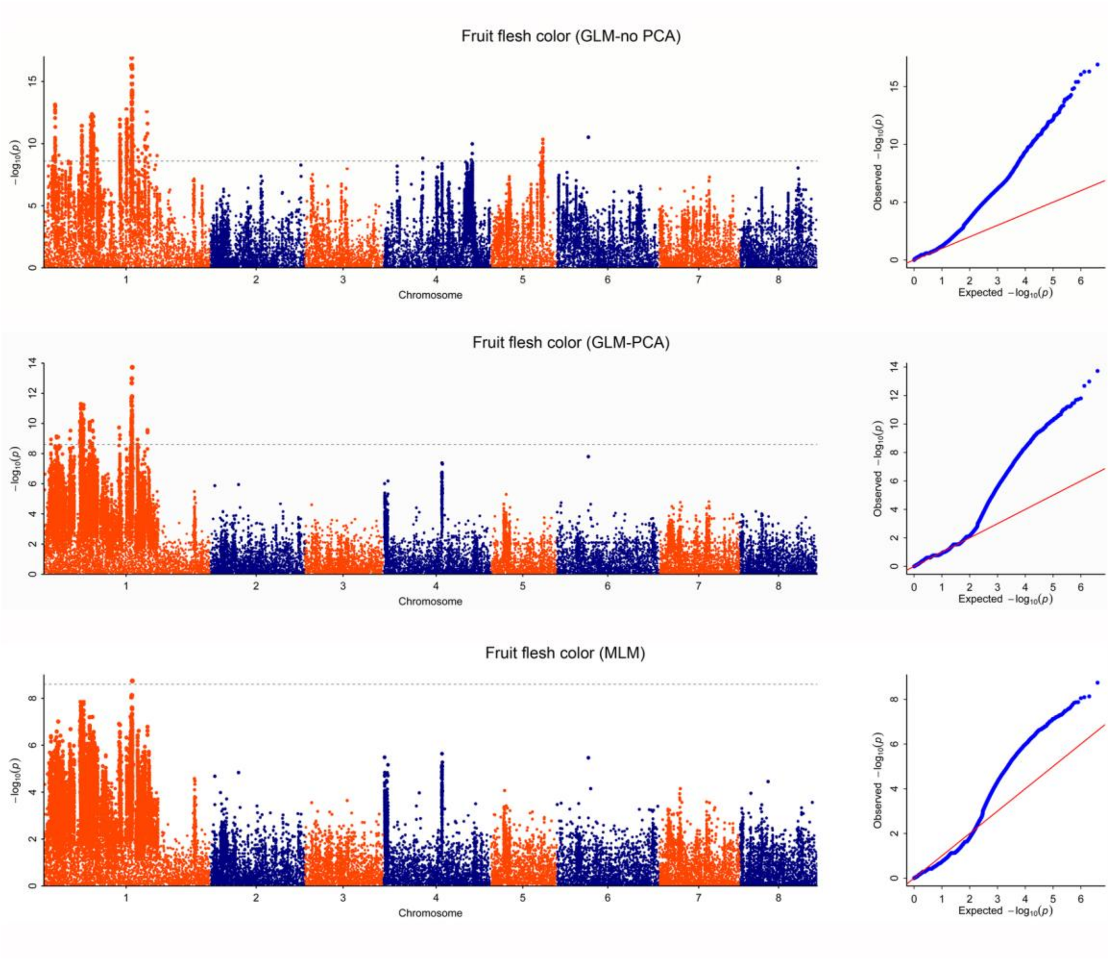

Supplementary Figure 7: Manhattan and quantile-quantile plots for Genome-wide association studies of fruit flesh color trait in 129 peach accessions using three analysis models.

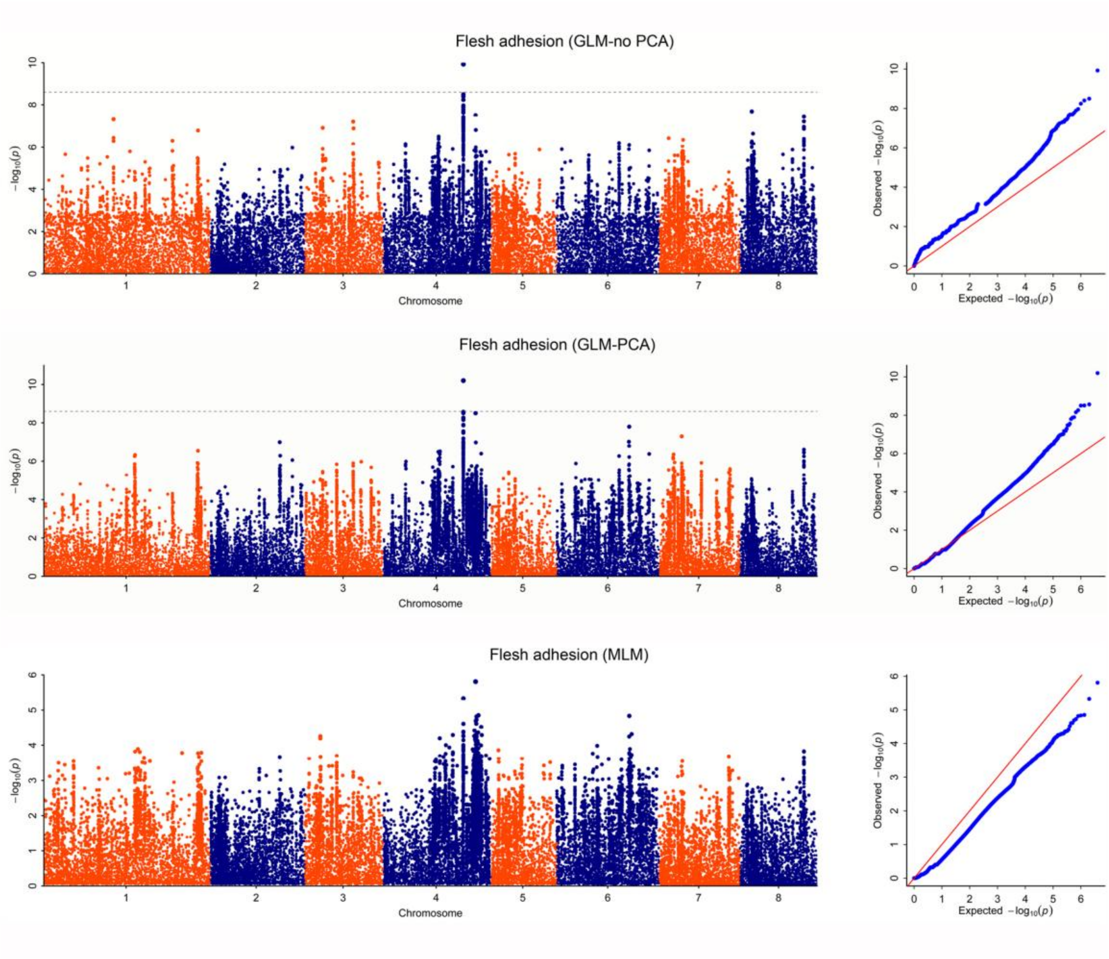

Supplementary Figure 8: Manhattan and quantile-quantile plots for Genome-wide association studies of fruit flesh adhesion trait in 129 peach accessions using three analysis models.

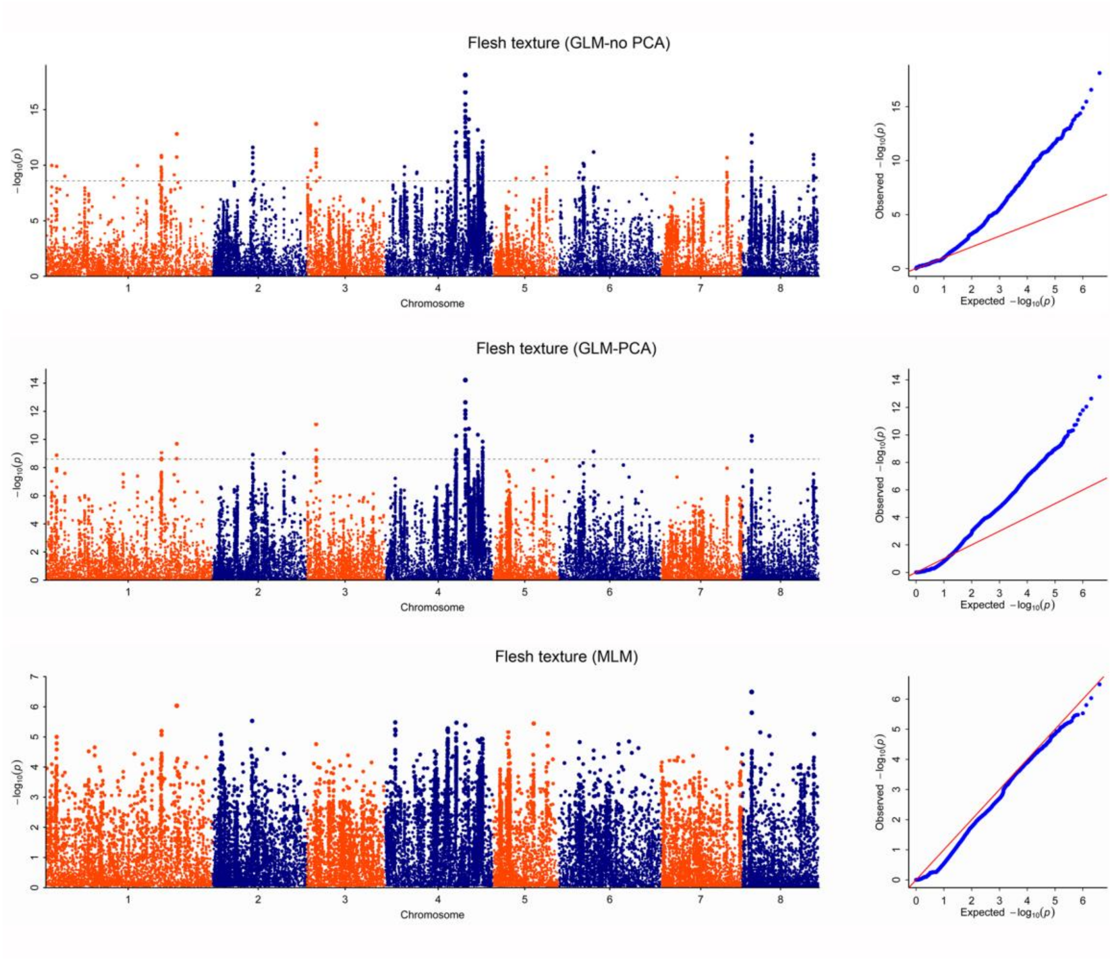

Supplementary Figure 9: Manhattan and quantile-quantile plots for Genome-wide association studies of fruit flesh texture trait in 129 peach accessions using three analysis models.

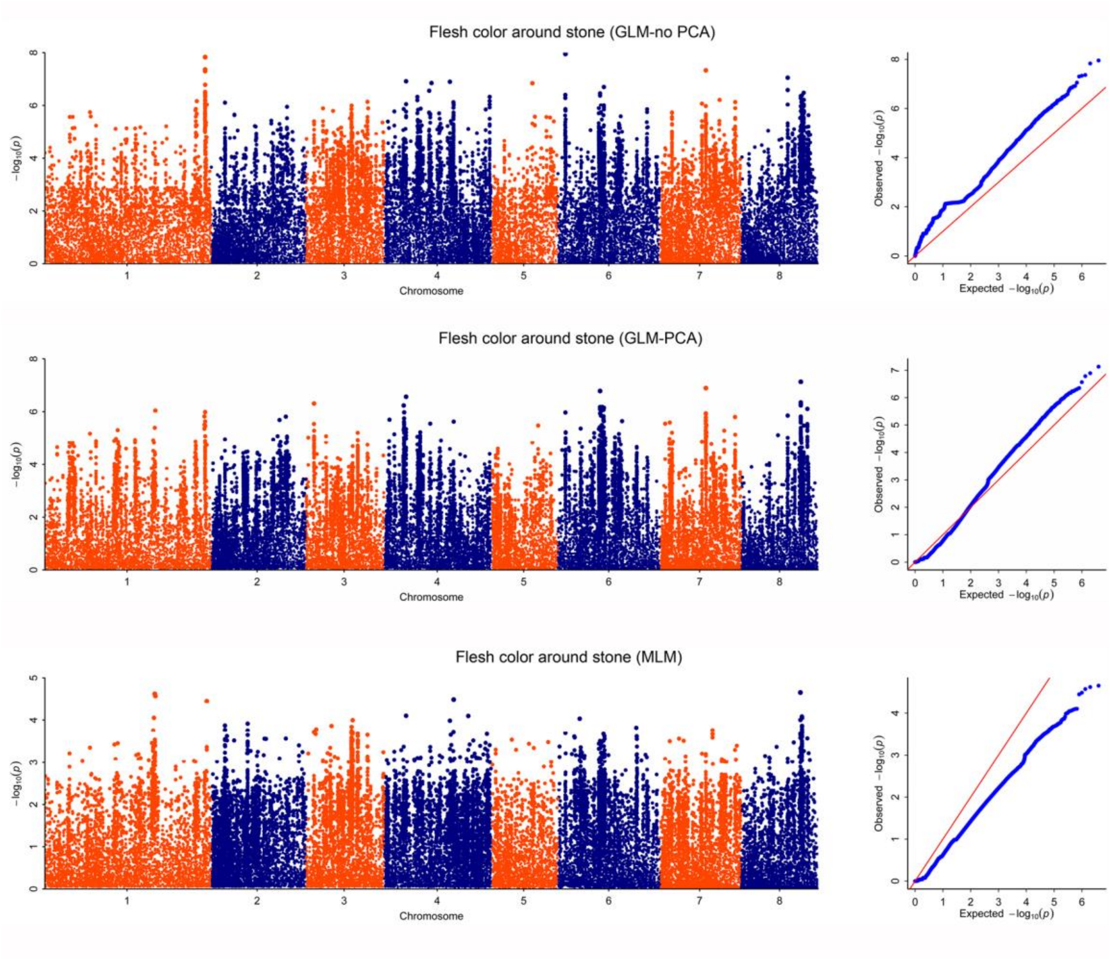

Supplementary Figure 10: Manhattan and quantile-quantile plots for Genome-wide association studies of flesh color around stone trait in 129 peach accessions using three analysis models.

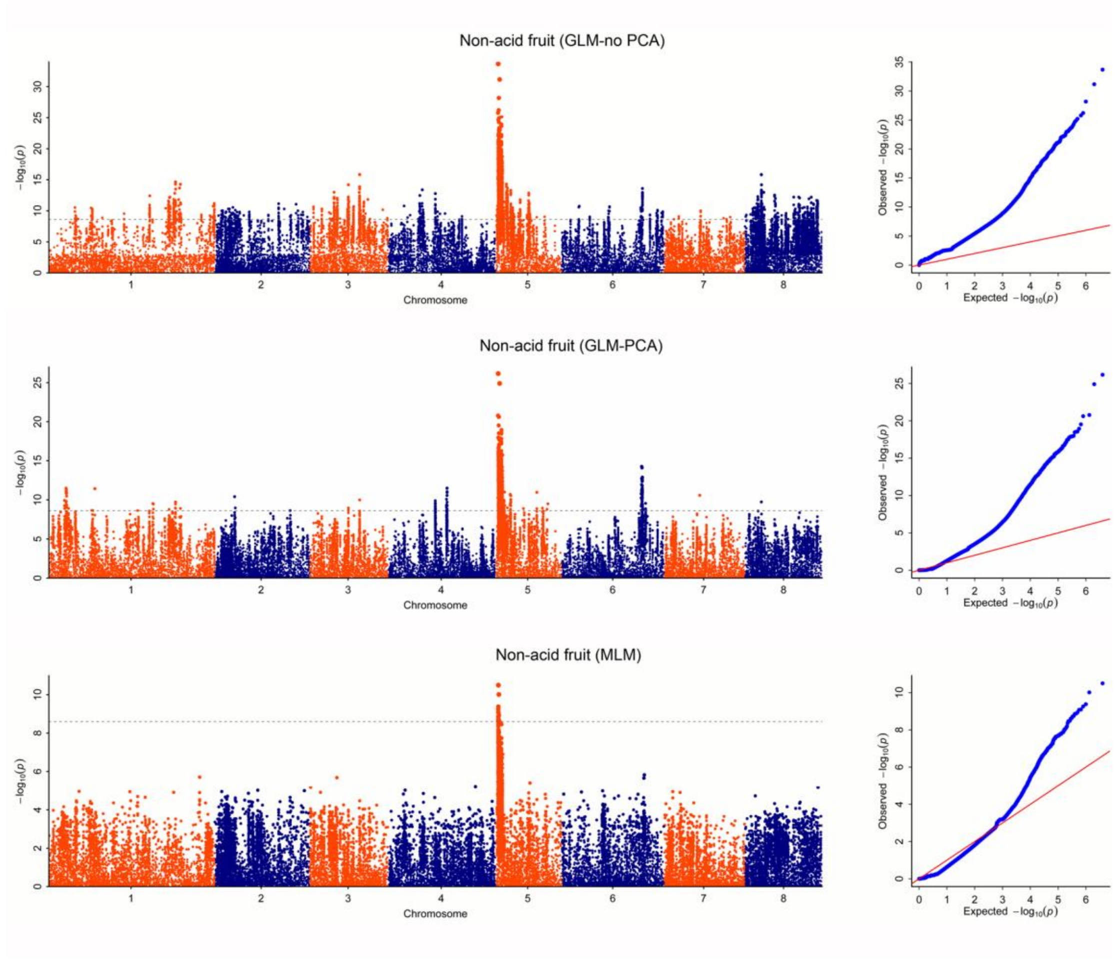

Supplementary Figure 11: Manhattan and quantile-quantile plots for Genome-wide association studies of non-acid fruit trait in 129 peach accessions using three analysis models.

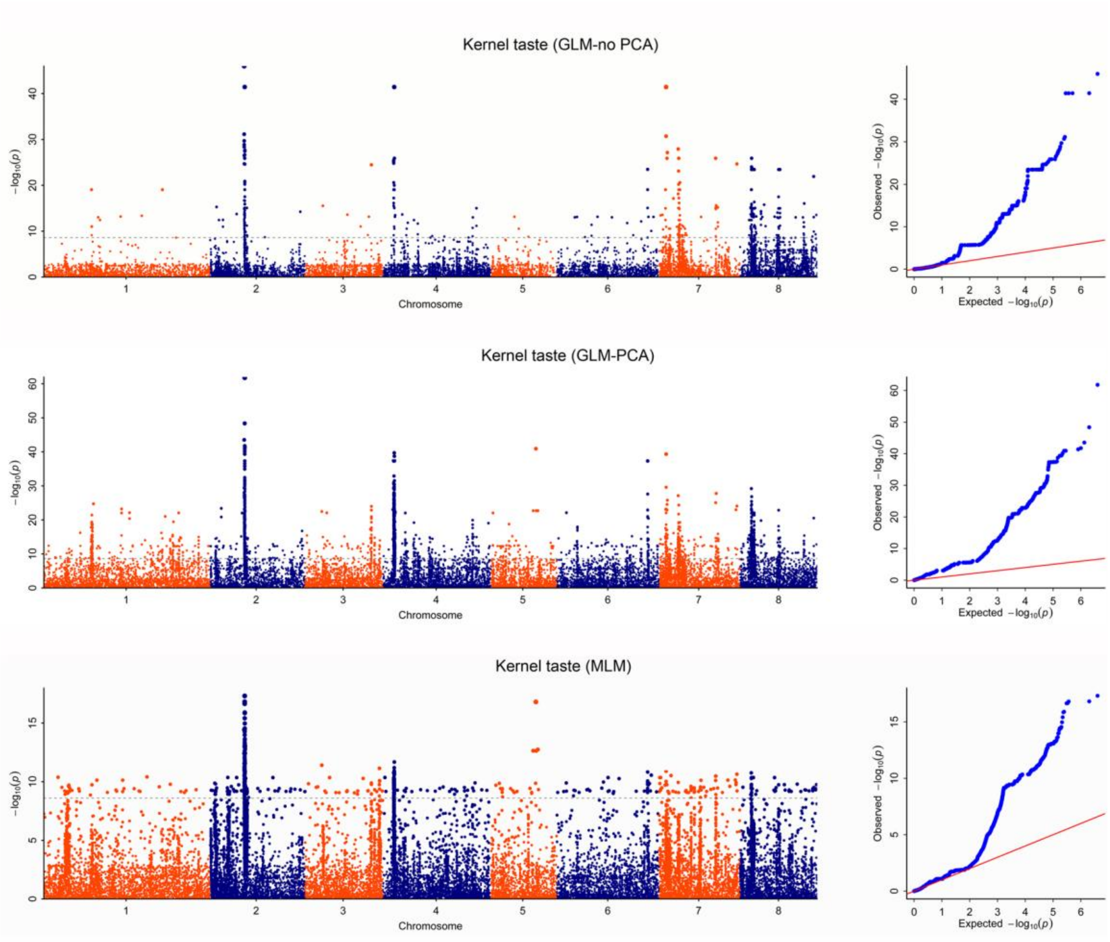

Supplementary Figure 12: Manhattan and quantile-quantile plots for Genome-wide association studies of kernel taste trait in 129 peach accessions using three analysis models.

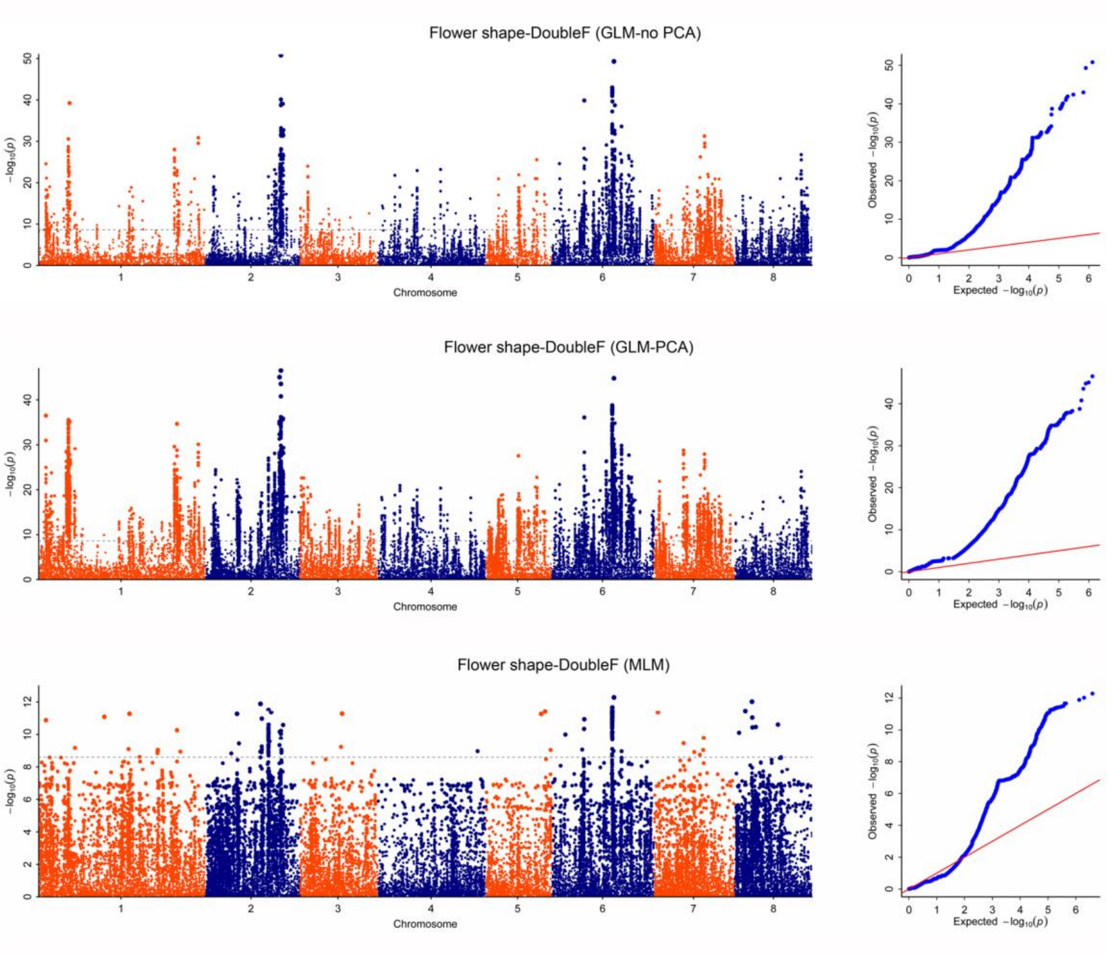

Supplementary Figure 13: Manhattan and quantile-quantile plots for Genome-wide association studies of flower shape-DoubleF trait in 129 peach accessions using three analysis models.

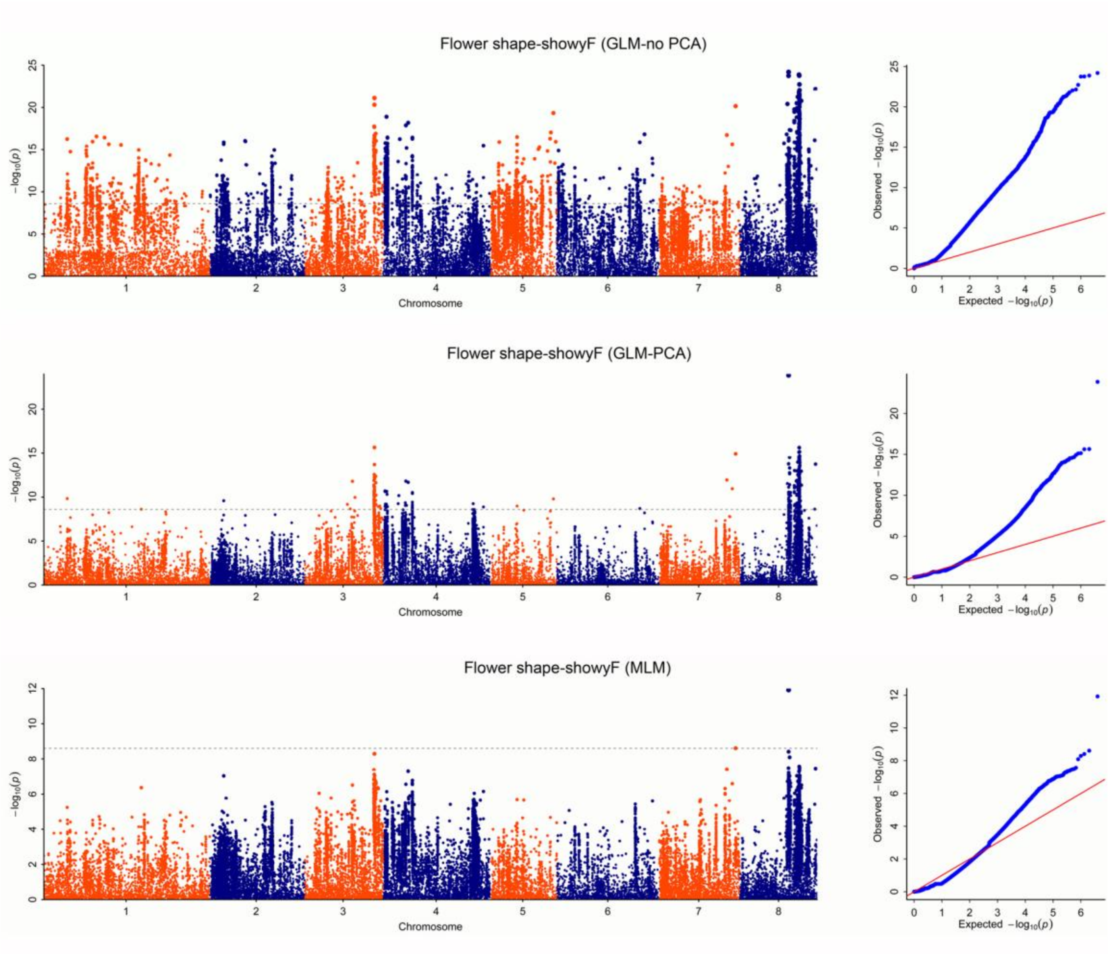

Supplementary Figure 14: Manhattan and quantile-quantile plots for Genome-wide association studies of flower shape-showyF trait in 129 peach accessions using three analysis models.

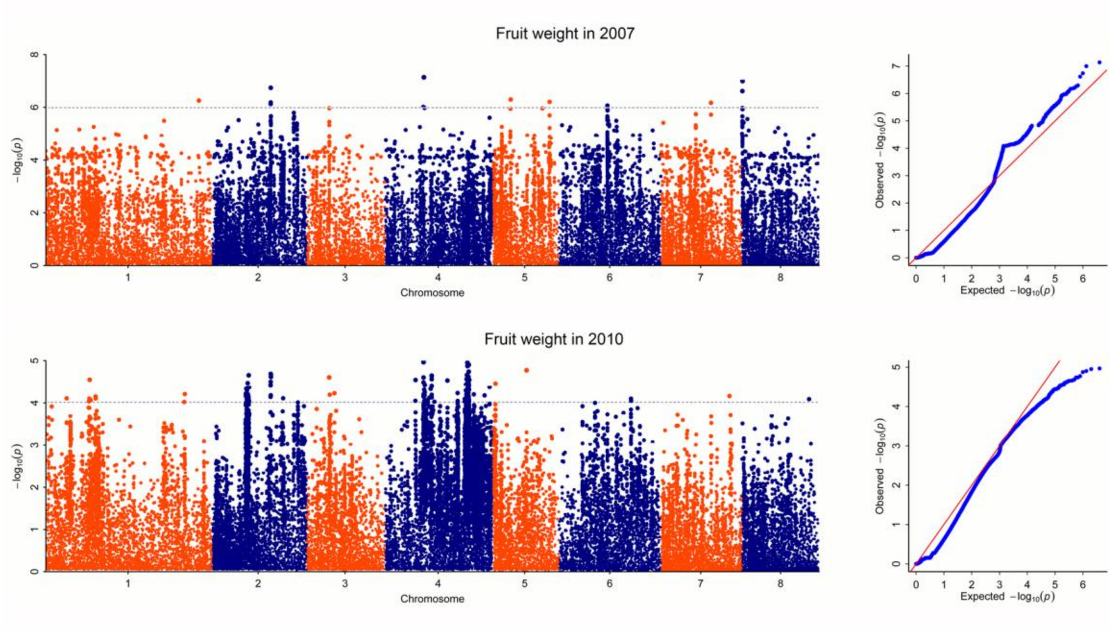

Supplementary Figure 15: Manhattan and quantile-quantile plots for Genome-wide association studies of fruit weight trait in 2007 and 2010 in 129 peach accessions using three analysis models. Negative $\log_{10}$-transformed P values from the genome-wide scan were plotted against SNPs position on each of the eight chromosomes.

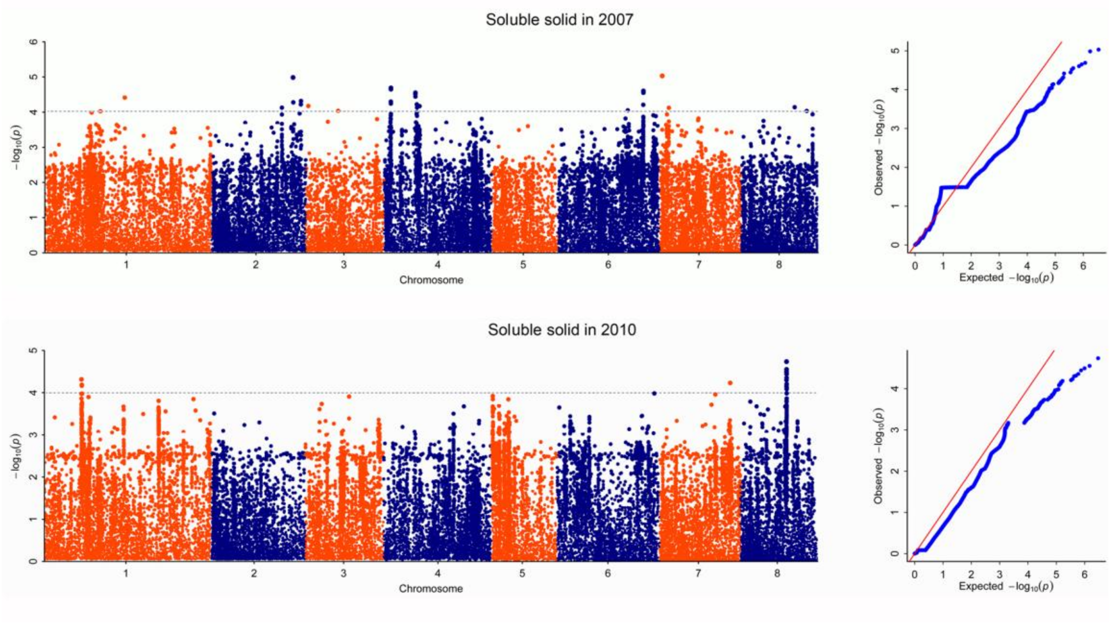

Supplementary Figure 16: Manhattan and quantile-quantile plots for Genome-wide association studies of soluble solid trait in 2007 and 2010 in 129 peach accessions using three analysis models. Negative log_{10}-transformed P values from the genome-wide scan were plotted against SNPs position on each of the eight chromosomes.

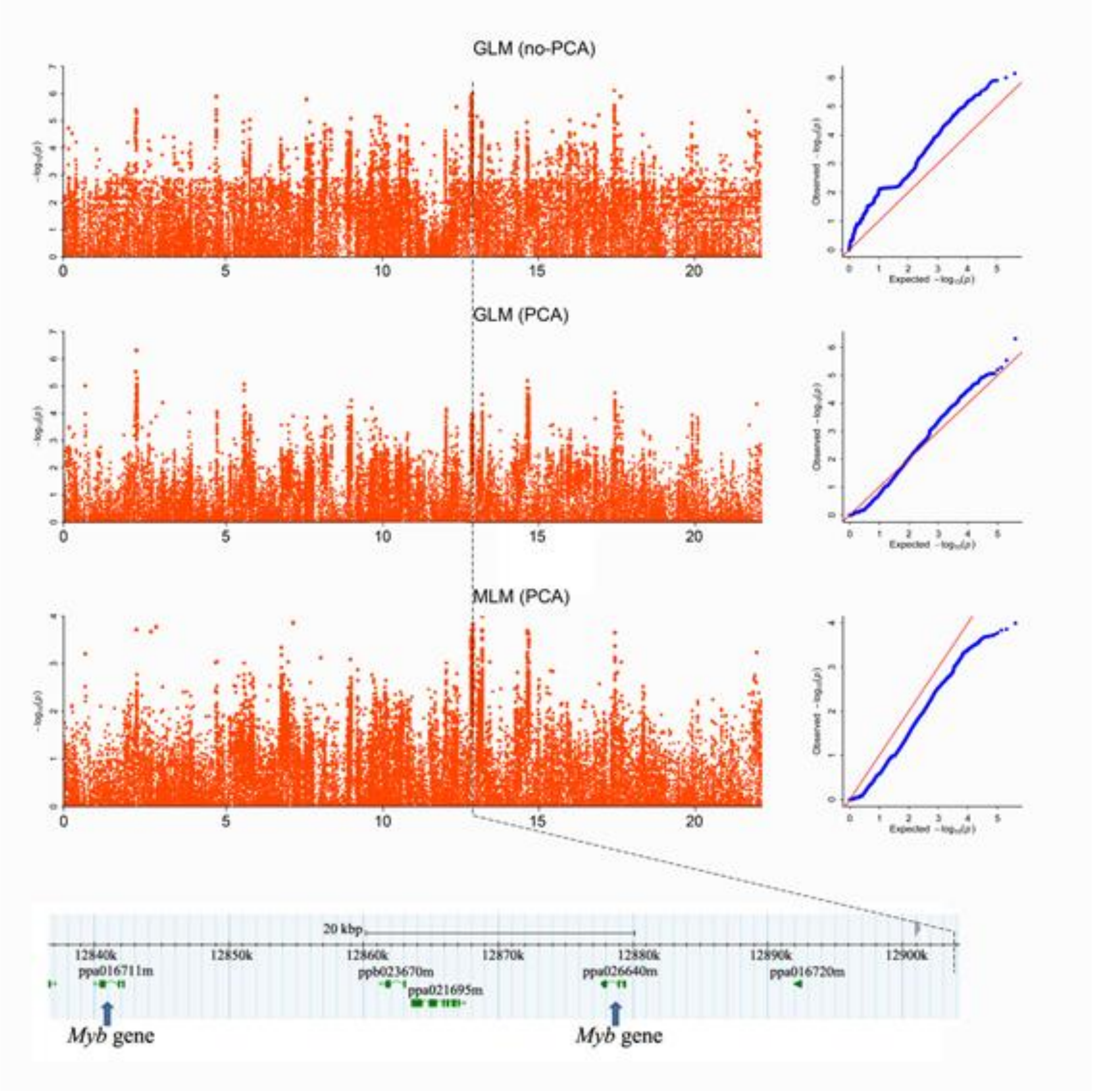

Supplementary Figure 17: Genome-wide association study of the flesh color around the stone trait in 129 peach accessions using the SNPs detected on scaffold_3. Although the highest association signal for this trait was found in chromosome 6 (Supplementary Fig. 10), its *P* value was lower than genome-wide levels (-$\log_{10} P = 8$) and this region was not reported in previous research. A SNP association peak was also found on scaffold_3: 12,902,851 bp, near two *MYB* genes (*ppa016711m* and *ppa026640m*). One of these genes (*ppa016711m*) was reported by Wang[26] as a key regulator of anthocyanin content in peach.

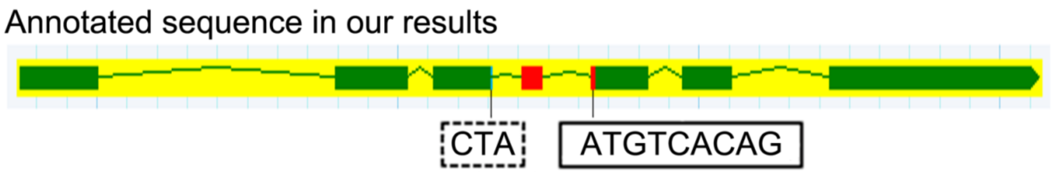

Supplementary Figure 18: The annotated transcriptional structure of the candidate gene *ppa003772m*. In our study, *ppa003772m* was considered a candidate gene for flat peach shape (Figure 1). After cloning the *ppa003772m* gene in flat and round peach accessions, their coding frames differed from those annotated in the peach genome (www.rosaceae.org). A new exon (shown in red) was present in the area corresponding to the third intron of all sampled materials. And three bases (CTA) in the tail of the original third exon (in green) were absent in the new annotated sequence. In addition to this, nine new bases (ATGTCACAG) were present in the head of the fifth exon of the new annotated gene. Moreover, the original annotated mRNA sequence was not found in peach tissues, indicating that the previous gene annotation might be incorrect.

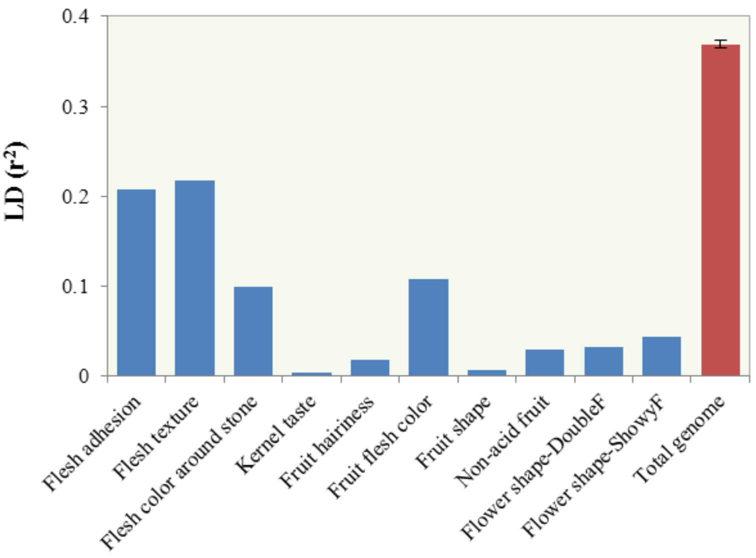

Supplementary Figure 19: The average linkage disequilibrium (LD, r^2) of peak association signal of 10 qualitative characters with each SNP during surrounding 50-kb region and the average r^2 in contiguous 50-kb windows across the peach pseudo-chromosomes. Peak association signals are listed in Table 1 and the LD across the chromosomes is shown in Supplementary Figure 20. This figure shows that the average LD of peak association signals of 10 qualitative characters with each SNP during surrounding 50-kb region (blue columns) were lower than the average LD across the eight peach chromosomes (red column), particularly those of kernel taste and fruit shape. Error bars indicate s.d.

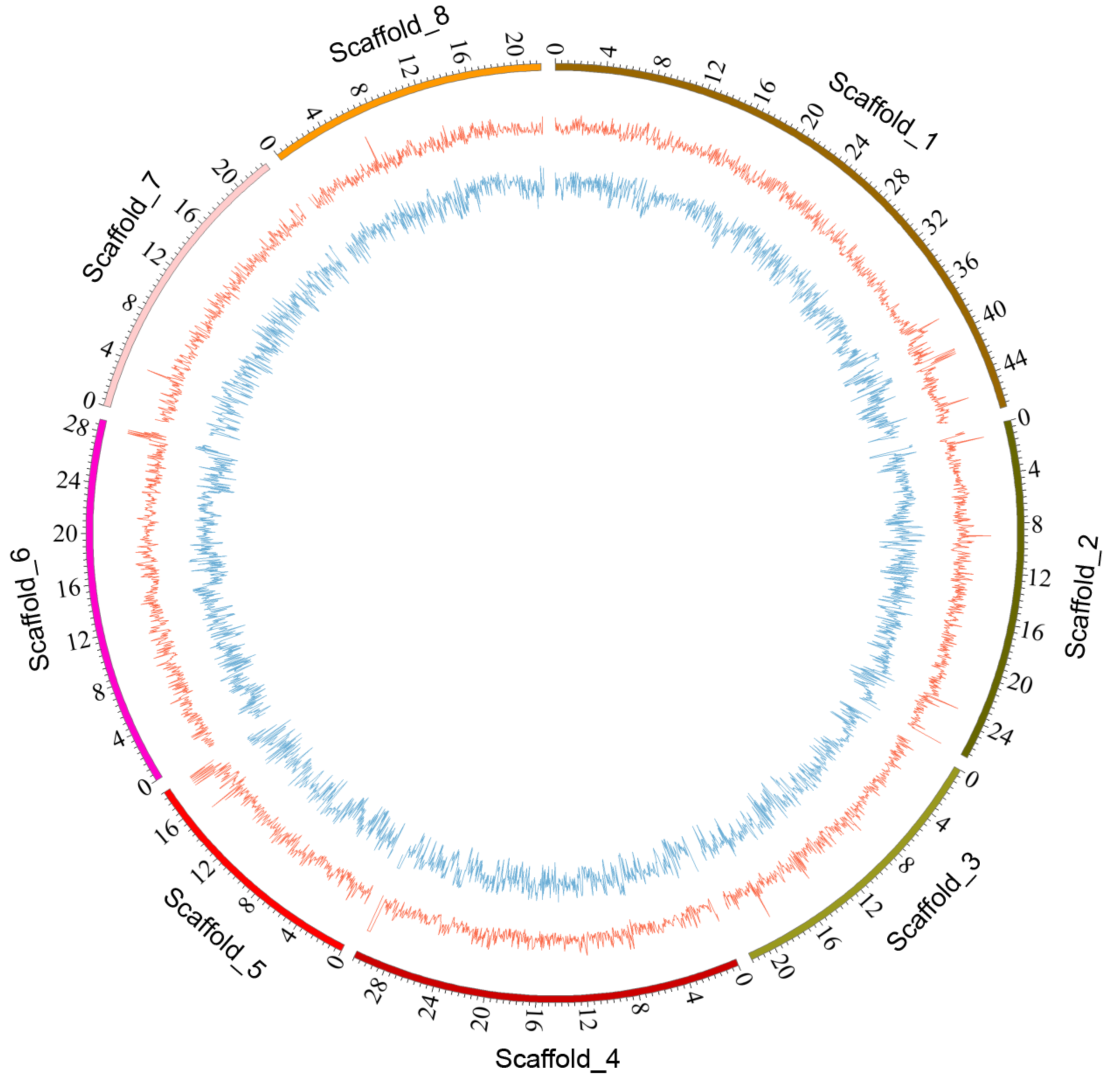

Supplementary Figure 20: Linkage disequilibrium (LD) in contiguous 50-kb windows across peach chromosomes. The outer rings indicate individual chromosomes and the numbers indicate physical locations in Mb. r^2 values are displayed in the inner blue circle and D′ in the middle red circle.

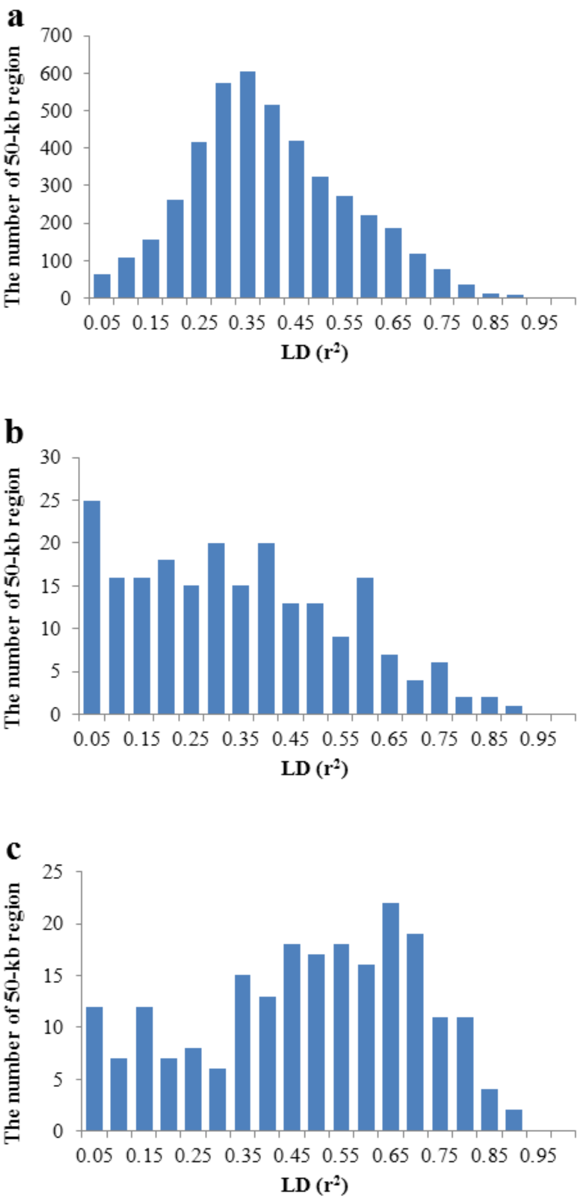

Supplementary Figure 21: Distribution of a LD value (r^2) in contiguous 50-kb windows across total (a), domestication (b), and improvement selection (c) regions in peach chromosomes.

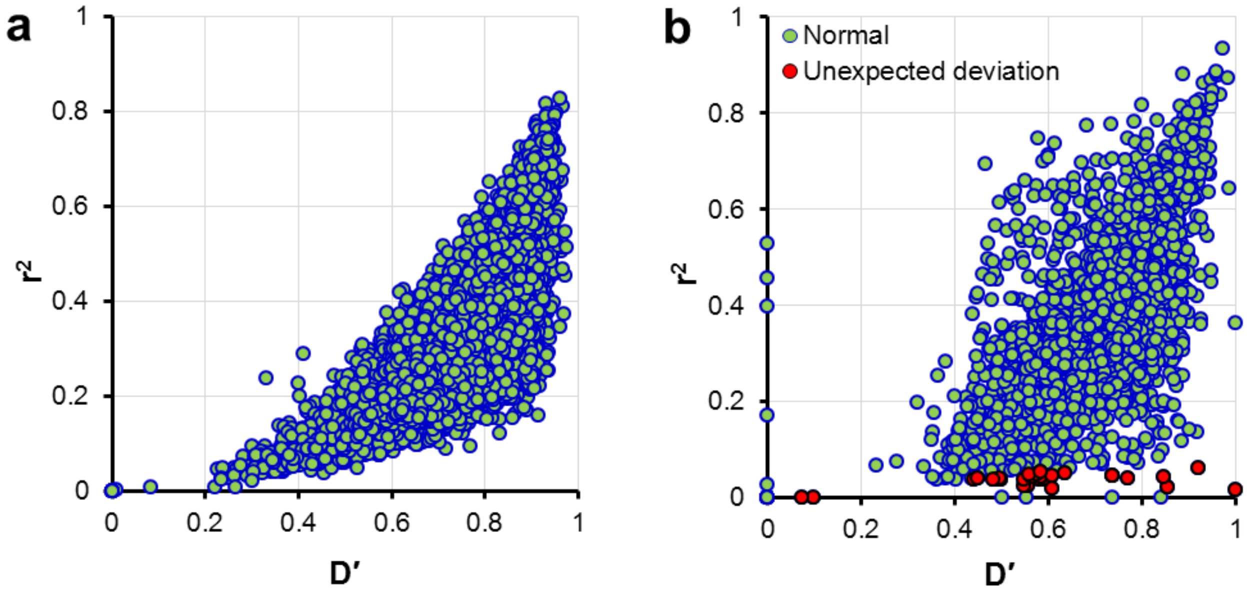

Supplementary Figure 22: The relationship between two parameters (r^2 and D') for estimating linkage disequilibrium (LD) in contiguous 50-kb genomic windows in soybean and peach. LD values (D′ and r^2) vary from 0 to 1 lies along the X and Y axis. (**a**) Due to the lack of D' values from soybean, r^2 values presented in this plot were recalculated rely on data from previous study[43] and the relationships between D′ and r^2 are shown as light green dots. In general, r^2 was lower than D′ in each given interval. (**b**) Regions with low r^2 and high D′ in peach were considered as normal and are also shown as light green dots, whereas regions with extraordinary low r^2 are shown as red dots. These regions are under domestication or improved selection.

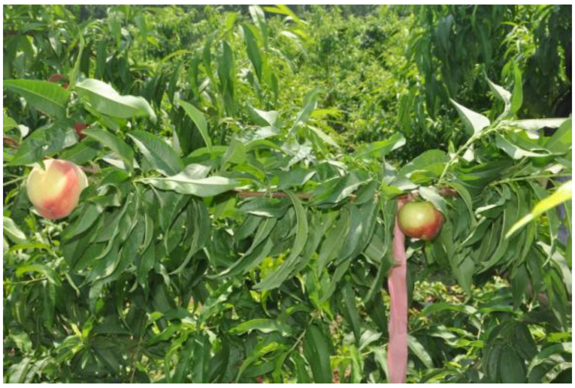

Peach burst into Nectarine

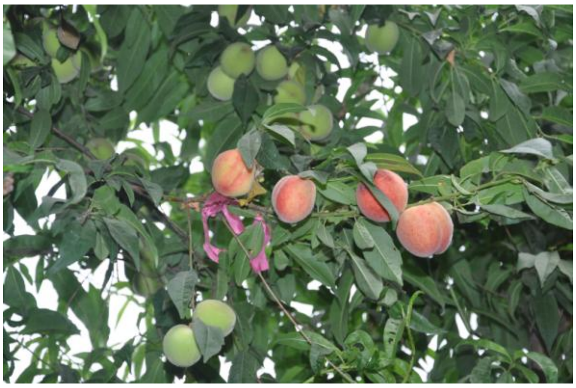

Late mature burst into early mature fruit

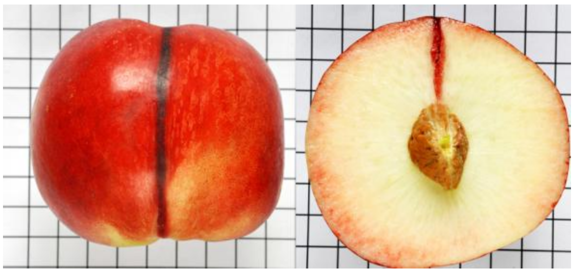

White flesh burst into red flesh in suture line of fruit

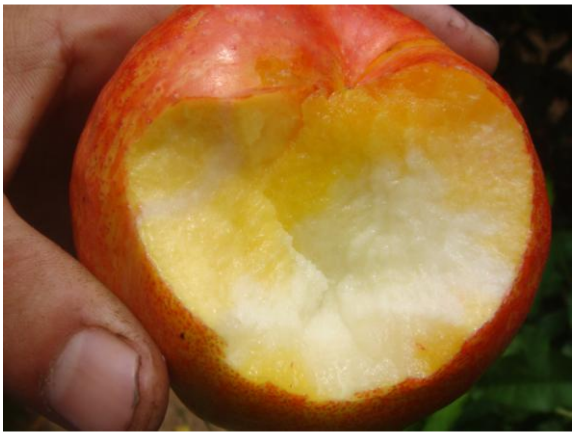

White flesh burst into yellow flesh

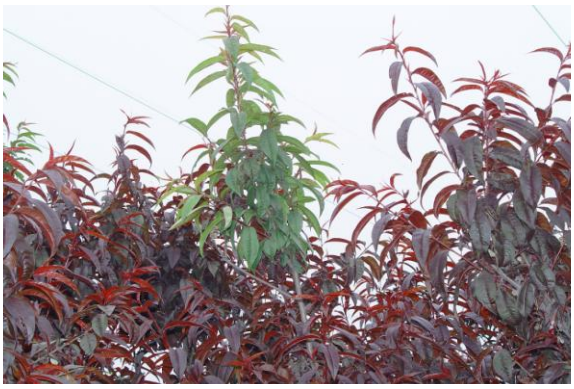

Red leaf burst into green leaf

Supplementary Figure 23: Peach mutation.

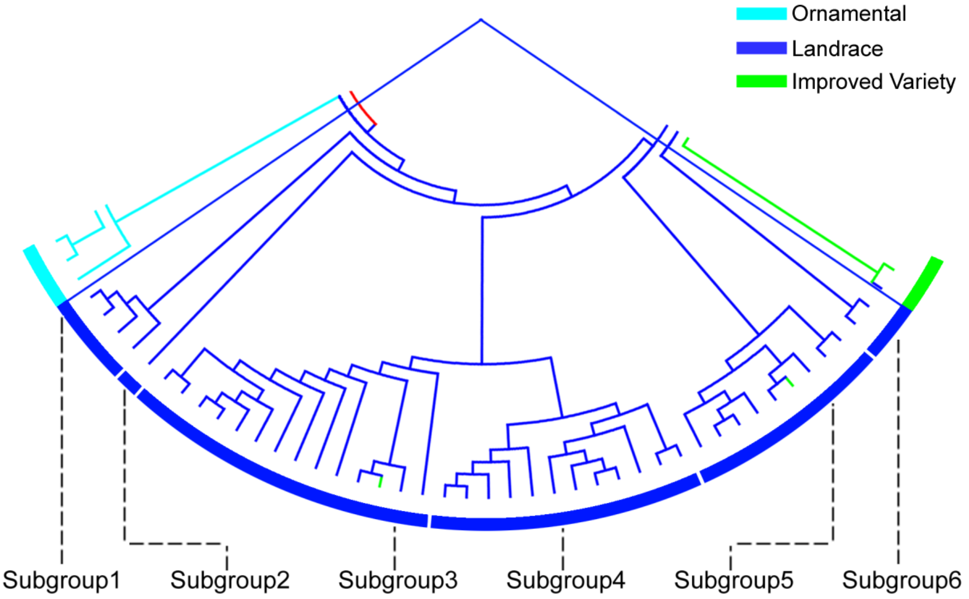

Supplementary Figure 24: Edible landraces subgroups as defined by the phylogenetic analysis based on total SNPs. This is an enlarged section of Supplementary Fig. 3a, only focusing on the edible landrace group. According to the phylogenetic tree, the edible landrace group (blue lines in the above figure) can be divided into six subgroups. Ornamental (cyan lines) and Improved varieties (green line) are not considered in this figure.

Supplementary Table 1: Peach samples added in this study compared to a previous one[7].

Name	Scientific name	Description and origin
**07-1-11**	*P. persica* (L.) Batsch.	Improved variety from crosses, Henan, PRC
**07-2-24**	*P. persica* (L.) Batsch.	Improved variety from crosses, Henan, PRC
**07-4-33**	*P. persica* (L.) Batsch.	Improved variety from crosses, Henan, PRC
**08-X-77**	*P. persica* (L.) Batsch.	Improved variety from crosses, Henan, PRC
**09-12 west-5**	*P. persica* (L.) Batsch.	Improved variety from crosses, Henan, PRC
**Bailey**	*P. persica* (L.) Batsch.	Improved variety from crosses, USA
**Da Guo Hei Tao**	*P. persica* (L.) Batsch.	Landrace, edible, Shandong, PRC
**Da Lian 4-35**	*P. persica* (L.) Batsch.	Improved variety from crosses, Liaoning, PRC
**Dan Bei Ti**	*P. persica* (L.) Batsch.	Improved variety from mutation, Henan, PRC
**Elberta (Non-showy flower)**	*P. persica* (L.) Batsch.	Improved variety from crosses, USA
**Fen Shou Xing**	*P. persica* (L.) Batsch.	Landrace, Ornamental, Hebei, PRC
**Harrow Blood**	*P. persica* (L.) Batsch.	Improved variety from crosses, Canada
**Hong Hua Bi Tao**	*P. persica* (L.) Batsch.	Landrace, edible, Beijing, PRC
**Hong Shan Hu**	*P. persica* (L.) Batsch.	Improved variety from crosses, Beijing, PRC
**Hong Ye Tao**	*P. persica* (L.) Batsch.	Landrace, edible, Henan, PRC
**Hong Ye Tao Ya Bian**	*P. persica* (L.) Batsch.	Improved variety from mutation, Henan, PRC
**Huang Jin Pan Tao**	*P. persica* (L.) Batsch.	Landrace, edible, Shanghai, PRC
**Legrand**	*P. persica* (L.) Batsch.	Improved variety from crosses, USA
**Mai Huang Pan Tao**	*P. persica* (L.) Batsch.	Improved variety from crosses, Henan, PRC
**Mao Tao (2-1-55)**	*P. persica* (L.) Batsch.	Improved variety from mutation, Henan, PRC
**Maria Serena**	*P. persica* (L.) Batsch.	Improved variety from crosses, Italy
**NJC77**	*P. persica* (L.) Batsch.	Improved variety from crosses, USA
**Nonpareil**	*P. communis* (L.) Fritsch.	Wild, USA
**Phillips**	*P. persica* (L.) Batsch.	Improved variety from crosses, USA
**Ping Ding You Pan Tao**	*P. persica* (L.) Batsch.	Improved variety from crosses, Henan, PRC
**Redhaven**	*P. persica* (L.) Batsch.	Improved variety from crosses, USA
**Rui Guang 3**	*P. persica* (L.) Batsch.	Improved variety from crosses, Beijing, PRC
**Shu Guang Hong Xian Ya Bian**	*P. persica* (L.) Batsch.	Improved variety from mutation, Henan, PRC
**Wu Han 2**	*P. persica* (L.) Batsch.	Improved variety from crosses, Shaanxi, PRC
**Xi Kang Bian Tao 1**	*P. tangutica* (L.) Batal.	Wild, Sichuan, PRC
**Xin Jiang Pan Tao**	*P. ferganensis* Kost.et Riab.	Landrace, edible, Sinkiang, PRC
**Yang Zhou 3**	*P. persica* (L.) Batsch.	Improved variety from crosses, Jiangsu, PRC
**Ying Zui Tao**	*P. persica* (L.) Batsch.	Landrace, edible, Anhui, PRC
**Zao Huang Pan Tao**	*P. persica* (L.) Batsch.	Improved variety from crosses, Henan, PRC
**Zao Shu Xia Ye Tao**	*P. persica* (L.) Batsch.	Improved variety from mutation, Henan, PRC
**Zhong You Pan Tao 2**	*P. persica* (L.) Batsch.	Improved variety from crosses, Henan, PRC
**Zhong You Pan Tao 4**	*P. persica* (L.) Batsch.	Improved variety from crosses, Henan, PRC
**Zhong You Tao 4**	*P. persica* (L.) Batsch.	Improved variety from crosses, Henan, PRC
**Zhong You Tao 4 Zao Shu Ya Bian**	*P. persica* (L.) Batsch.	Improved variety from mutation, Anhui, PRC

**Zhong You Tao 5**	*P. persica* (L.) Batsch.	Improved variety from crosses, Henan, PRC
**Zhong You Tao 5 Zao Shu Ya Bian**	*P. persica* (L.) Batsch.	Improved variety from mutation, Guangxi, PRC
**Zhong You Tao 9**	*P. persica* (L.) Batsch.	Improved variety from crosses, Henan, PRC
**Zhong You Tao 9 Hei Xian Bai Rou Ya Bian**	*P. persica* (L.) Batsch.	Improved variety from mutation, Anhui, PRC
**Zhong You Tao 9 Huang Rou Ya Bian**	*P. persica* (L.) Batsch.	Improved variety from mutation, Anhui, PRC
**Zhong You Tao 9 Wang Shu Ya Bian**	*P. persica* (L.) Batsch.	Improved variety from mutation, Henan, PRC

Supplementary Table 2: Agronomic traits of peach samples used in this study.

Name	Fruit hairiness	Fruit shape	Fruit flesh color	Flesh adhesion	Flesh texture	Flesh color around stone	Non-acid fruit	Flower shape-DoubleF	Flower shape-ShowyF	Kernel taste	Fruit weight in 2007 (g)	Fruit weight in 2010 (g)	Soluble solid in 2007 (%)	Soluble solid in 2010 (%)
**07-1-11**	No	Round	White	Clingstone	Melting	Yes	No	Single	NA	Bitter	NA	130	NA	13
**07-2-24**	Yes	Round	White	Clingstone	Melting	Yes	Yes	Single	Showy	Bitter	NA	170	NA	12
**07-4-33**	NA	NA	NA	NA	NA	NA	NA	NA	NA	NA	NA	NA	NA	NA
**08-X-77**	NA	NA	NA	NA	NA	NA	NA	Single	NA	Bitter	NA	NA	NA	NA
**09-12 west-5**	Yes	Flat	White	Clingstone	Melting	NA	No	Single	NA	Bitter	NA	110	NA	NA
**Bailey**	Yes	Round	White	Freestone	Melting	Yes	No	Single	Showy	Bitter	65.2	79	11	NA
**Da Guo Hei Tao**	Yes	Round	White	Freestone	Melting	Yes	No	Single	Showy	Bitter	187	146	12	NA
**Da Lian 4-35**	Yes	Flat	Yellow	Freestone	Melting	No	Yes	Single	Showy	Bitter	100	128.9	11	NA
**Dan Bei Ti**	No	Round	Yellow	Clingstone	Melting	NA	NA	Single	Non-showy	NA	NA	NA	NA	NA
**Elberta (Non-showy flower)**	Yes	Round	Yellow	Freestone	Melting	Yes	No	Single	Non-showy	Bitter	198	142.5	10	8
**Fen Shou Xing**	Yes	Round	White	Freestone	Melting	Yes	No	Single	Showy	Bitter	79.4	89	7	NA
**Harrow Blood**	Yes	Round	White	Freestone	Melting	Yes	No	Single	Showy	Bitter	NA	47	NA	18
**Hong Hua Bi Tao**	Yes	Round	White	Freestone	Melting	No	No	Multiple	Showy	Bitter	25	NA	11	NA
**Hong Shan Hu**	No	Round	White	Clingstone	Melting	No	Yes	Single	Non-showy	Bitter	148	151	13	NA
**Hong Ye Tao**	Yes	Round	White	NA	Melting	No	No	Multiple	Showy	Bitter	81.9	76	14	NA
**Hong Ye Tao Ya Bian**	Yes	Round	White	NA	Melting	No	No	Multiple	Showy	Bitter	NA	96	10	NA
**Huang Jin Pan Tao**	Yes	Flat	Yellow	Clingstone	Melting	Yes	Yes	Single	Showy	Bitter	112	97.7	12.5	11.5
**Legrand**	No	Round	Yellow	Freestone	Melting	Yes	No	Single	Showy	Sweet	151	126.7	11.5	11
**Mai Huang Pan Tao**	Yes	Flat	Yellow	Clingstone	Melting	No	Yes	Single	Showy	Bitter	89.6	70	NA	11
**Mao Tao (2-1-55)**	Yes	Round	Yellow	Clingstone	Melting	Yes	Yes	Single	Showy	Bitter	78	NA	11	NA
**Maria Serena**	Yes	Round	Yellow	Clingstone	Non-melting	No	No	Single	Showy	Bitter	130	113.7	7.6	7

**NJC77**	Yes	Round	Yellow	Clingstone	Non-melting	No	No	Single	Non-showy	Bitter	157.3	112.3	12.6	10
**Nonpareil**	Yes	Round	NA	Freestone	Melting	NA	NA	NA	Showy	Sweet	NA	NA	NA	NA
**Phillips**	Yes	Round	Yellow	Clingstone	Non-melting	No	No	Single	Non-showy	Bitter	135	116.5	12.9	12.5
**Ping Ding You Pan Tao**	No	Flat	Yellow	Freestone	Melting	No	No	Single	Showy	Bitter	82	78.9	10	NA
**Redhaven**	Yes	Round	Yellow	Freestone	Melting	Yes	No	Single	Non-showy	Bitter	124.1	130	10	9
**Rui Guang 3**	No	Round	White	Freestone	Melting	Yes	Yes	Single	Non-showy	Bitter	123.8	144.5	12	7
**Shu Guang Hong Xian Ya Bian**	No	Round	Yellow	NA	Melting	Yes	Yes	Single	Showy	Bitter	NA	102	13.5	NA
**Wu Han 2**	Yes	Round	White	Freestone	Melting	Yes	Yes	Single	Showy	Bitter	96	90	12.6	11
**Xi Kang Bian Tao 1**	Yes	Round	White	Freestone	Melting	NA	NA	NA	Showy	Bitter	NA	5	NA	NA
**Xin Jiang Pan Tao**	Yes	Flat	White	Freestone	Melting	No	No	Single	Showy	Bitter	52	NA	13	NA
**Yang Zhou 3**	Yes	Round	White	Freestone	Melting	No	No	Single	Showy	Bitter	150	80	12.6	10
**Ying Zui**	Yes	Round	White	Clingstone	Non-melting	Yes	No	Single	Showy	Bitter	257	160	11	10
**Zao Huang Pan Tao**	Yes	Flat	Yellow	Freestone	Melting	No	Yes	Single	Showy	Bitter	116.7	88	NA	11
**Zao Shu Xia Ye Tao**	NA	NA	NA	NA	NA	NA	NA	Single	NA	NA	NA	NA	NA	NA
**Zhong You Pan Tao 2**	No	Flat	Yellow	Clingstone	Melting	No	Yes	Single	Non-showy	Bitter	80	83.5	18	NA
**Zhong You Pan Tao 4**	No	Flat	Yellow	Clingstone	Non-melting	Yes	Yes	Single	Non-showy	Bitter	120	NA	18	NA
**Zhong You Tao 4**	No	Round	Yellow	Clingstone	Melting	Yes	Yes	Single	Non-showy	Bitter	NA	150	NA	11
**Zhong You Tao 4 Zao Shu Ya Bian**	No	Round	Yellow	NA	Melting	Yes	Yes	Single	Non-showy	Bitter	NA	150	NA	11
**Zhong You Tao 5**	No	Round	White	Clingstone	Melting	No	Yes	Single	Non-showy	Bitter	110.1	150	NA	8
**Zhong You Tao 5 Zao Shu Ya Bian**	No	Round	White	NA	Melting	No	Yes	Single	Non-showy	Bitter	133	125.1	10	NA
**Zhong You Tao 9**	No	Round	White	Clingstone	Melting	No	Yes	Single	Non-showy	Bitter	143.1	161	10	NA
**Zhong You Tao 9 Hei Xian Bai Rou Ya Bian**	No	Round	White	NA	Melting	Yes	Yes	Single	Non-showy	Bitter	NA	216	NA	15

**Zhong You Tao 9 Huang Rou Ya Bian**	No	Round	Yellow	NA	Melting	NA	Yes	Single	Non-showy	NA	NA	161	NA	10
**Zhong You Tao 9 Wang Shu Ya Bian**	No	Round	White	NA	Melting	No	Yes	Single	Non-showy	Bitter	NA	161	NA	10

NA means the data is missing.

Supplementary Table 3: Geographic origin of the edible landraces and the coverage, mean depth, and heterozygosity rate of all samples.

Name	Coverage (%)	Mean depth (×)	Missing rate (%)	Heterozygosity rate [before imputed] (%)	Missing rate [imputed] (%)	Heterozygosity rate [imputed] (%)	The geographic region of the edible landraces
**2007-1-11**	96.96	7.77	1.7	6.45	9.08	2.63	
**2007-2-24**	95.27	5.48	2.93	5.37	8	2.63	
**07-4-33**	95.21	5.23	3.75	4.91	7.54	2.63	
**08-X-77**	96.53	7.01	1.85	6.74	9.57	2.83	
**09-12 west-5**	96.07	6.21	2.53	6.95	10.16	3.21	
**Bai Gen Gan Su Tao**	Ref. 7	Ref. 7	18.75	1.05	2.76	1.7	
**Bai Hua**	Ref. 7	Ref. 7	19.67	1.81	4.65	2.84	MLCJ
**Bai Hua Shan Bi Tao**	Ref. 7	Ref. 7	20.67	8.43	22.32	13.89	
**Bai Hua Shan Tao**	Ref. 7	Ref. 7	16.07	4.79	9.23	4.45	
**Bai Shu 55　Bian Yi**	Ref. 7	Ref. 7	15.78	2.06	4.99	2.93	
**Bai Wu 8**	Ref. 7	Ref. 7	17.02	2.93	7.69	4.76	
**Bailey**	94.9	5.41	4.71	3.63	5.7	2.07	
**Bi Nan I**	Ref. 7	Ref. 7	10.63	0.58	1.02	0.43	NWC
**Bian Tao**	Ref. 7	Ref. 7	16.11	2.66	6.53	3.87	NC
**Chinese Cling**	Ref. 7	Ref. 7	17.69	2.28	5.7	3.41	MLCJ
**Chong Ban Xiao Hua Xing**	Ref. 7	Ref. 7	19.62	2.02	5.48	3.46	
**Da Guo Hei Tao**	93.64	6.36	3.48	1.03	1.66	0.63	NC
**Da Hong Pao**	Ref. 7	Ref. 7	18.75	2.36	6.25	3.89	MLCJ
**Da Lian 4-35**	95.42	6.99	2.56	4.14	5.99	1.85	
**Da Xue Tao**	Ref. 7	Ref. 7	11.79	1.7	3.25	1.55	NC
**Dan Bei Ti**	92.06	5.14	5.88	0.96	1.53	0.56	
**Diao Zhi Bai**	Ref. 7	Ref. 7	9.99	3.97	7.38	3.4	MLCJ
**Elberta (Non-showy flower)**	97.52	6.01	1.49	7.62	10.92	3.3	
**Fei Cheng Bai Li 10**	Ref. 7	Ref. 7	3.84	3.23	4.98	1.75	NC
**Fei Cheng Hong Li 6**	Ref. 7	Ref. 7	19.1	2.31	5.96	3.65	NC
**Feng Shou Xing**	84.2	2.54	16.1	0.83	1.8	0.97	
**Ge Gu**	Ref. 7	Ref. 7	16.11	2.25	5.17	2.92	NC
**Guang He Tao (A Ba)**	Ref. 7	Ref. 7	11.66	2.81	4.98	2.17	
**Guang He Tao (Ri Ka Ze)**	Ref. 7	Ref. 7	18.47	1.67	4.07	2.4	
**Hakuho**	Ref. 7	Ref. 7	18.44	2.34	6.08	3.75	
**Harrow Blood**	97.84	8.39	1.02	0.75	1.05	0.3	
**Hei Bu Dai**	Ref. 7	Ref. 7	16.06	2.88	6.53	3.65	MLCJ
**Hong Chui Zhi**	Ref. 7	Ref. 7	21.33	1.17	3.16	1.99	
**Hong Gen Gan Su Tao**	Ref. 7	Ref. 7	9.67	2.1	3.6	1.5	
**Hong Hua Bi Tao**	93.4	4.73	6.07	3.23	5.28	2.04	NC
**Hong Hua Shan Tao**	Ref. 7	Ref. 7	11.33	5.48	8.85	3.37	
**Hong Li Guang**	Ref. 7	Ref. 7	9.94	0.91	1.61	0.71	NWC
**Hong Rou Guang He Tao**	Ref. 7	Ref. 7	17.9	2.72	5.97	3.25	

**Hong Shan Hu**	96.82	6.22	1.77	5.56	7.97	2.41	
**Hong Shou Xing**	Ref. 7	Ref. 7	15.75	0.91	2.01	1.1	
**Hong Ya Zui**	Ref. 7	Ref. 7	10.35	3.64	6.97	3.32	NC
**Hong Ye Tao**	96.96	7.26	13.93	2.68	5.7	3.03	NC
**Hong Ye Tao Ya Bian**	86.8	2.95	1.7	5.01	7.12	2.1	
**Hua Guang**	Ref. 7	Ref. 7	7.48	3.36	6.16	2.8	
**Hua Yu Lu**	Ref. 7	Ref. 7	10.5	3.53	6.45	2.91	
**Huang Jin Pan Tao**	94.59	5.47	3.09	3.9	5.81	1.91	MLCJ
**Hun Chun Tao**	Ref. 7	Ref. 7	4.13	1.12	1.81	0.7	NEC
**Huo Lian Jin Dan**	Ref. 7	Ref. 7	3.55	3.65	5.53	1.88	YGC
**Ji Lin 8903**	Ref. 7	Ref. 7	16.19	2.27	5.52	3.25	NEC
**Jin Feng**	Ref. 7	Ref. 7	14.2	2.95	6.81	3.86	
**Jin Mi Xia Ye**	Ref. 7	Ref. 7	3.99	2.79	4.37	1.58	
**Jin Ta You Pan Tao**	Ref. 7	Ref. 7	12.04	1.55	3.08	1.53	NWC
**Jing Yu**	Ref. 7	Ref. 7	12.49	3.21	6.95	3.74	
**Ju Hua Tao**	Ref. 7	Ref. 7	17.4	1.09	2.53	1.44	
**Ka Shi 1**	Ref. 7	Ref. 7	15.12	0.86	1.8	0.95	NWC
**Ka Shi Huang Rou Li Guang**	Ref. 7	Ref. 7	2.8	4.21	6.31	2.1	NWC
**Legrand**	85.2	2.44	15.84	1.96	4.8	2.84	
**Li He Tian Ren**	Ref. 7	Ref. 7	2.48	1.14	1.72	0.58	NWC
**Li-07-1-13**	Ref. 7	Ref. 7	10.14	3.34	6.39	3.05	
**Long 1-2-4**	Ref. 7	Ref. 7	17.87	0.88	2.03	1.14	NWC
**Mai Huang Pan Tao**	97.36	7.64	1.33	6.11	8.55	2.44	
**Mao Tao (2-1-55)**	96.76	7.7	1.96	4.53	6.46	1.92	
**Maria Serena**	95.66	5.38	2.75	2.34	3.51	1.17	
**May Fire**	Ref. 7	Ref. 7	12.22	1.26	2.54	1.27	
**Mei Gui Hong**	Ref. 7	Ref. 7	13.14	3.38	7.56	4.18	
**Nan Shan Tian Tao**	Ref. 7	Ref. 7	5.89	3.4	5.55	2.14	SC
**NJC77**	92.45	5.63	6.66	2.23	3.55	1.32	
**NJN76**	Ref. 7	Ref. 7	10.75	1.62	3.1	1.48	
**Nonpareil**	73.52	5.34	13.82	2.57	4.43	1.86	
**Okitsu**	Ref. 7	Ref. 7	17.46	1.94	4.83	2.89	
**Okubo**	Ref. 7	Ref. 7	22.18	1.94	5.92	3.99	
**Phillips**	98	6.1	1.19	0.88	1.23	0.36	
**Ping Bei Zi**	Ref. 7	Ref. 7	12.06	3.38	6.82	3.44	MLCJ
**Ping Ding You Pan Tao**	96.02	7.82	2.3	4.19	5.97	1.78	
**Qing Si**	Ref. 7	Ref. 7	12.17	2.27	4.44	2.17	YGC
**Qing Tao**	Ref. 7	Ref. 7	15.96	3	7.34	4.34	YGC
**Qing Zhou Bai Pi Mi Tao**	Ref. 7	Ref. 7	3.75	1.41	2.22	0.82	NC
**Redhaven**	97.13	5.87	1.83	3.67	5.32	1.65	
**Rou Pan Tao**	Ref. 7	Ref. 7	15.25	0.91	1.9	0.99	NWC
**Rui Guang 2**	Ref. 7	Ref. 7	12.26	2.47	5.31	2.85	
**Rui Guang 3**	97.14	6.41	1.75	5.12	7.33	2.21	

**Sa Hong Long Zhu Tao**	Ref. 7	Ref. 7	15.37	0.68	1.41	0.73	
**Sa Hong Tao**	Ref. 7	Ref. 7	14.61	1.05	2.26	1.21	
**Sa Hua Hong Pan Tao**	Ref. 7	Ref. 7	4.06	4.71	7.25	2.54	MLCJ
**Shan Dong Si Yue Ban**	Ref. 7	Ref. 7	15.23	1.51	3.38	1.86	NC
**Shan Gan Shan Tao**	Ref. 7	Ref. 7	9.64	6.71	10.28	3.56	
**Shen Zhou Shui Mi**	Ref. 7	Ref. 7	16.98	2.22	5.43	3.21	NC
**Shi Tou Tao**	Ref. 7	Ref. 7	16.33	2.35	5.76	3.4	NC
**Shi Wo Shui Mi**	Ref. 7	Ref. 7	17	2.21	5.35	3.14	NC
**Shou Bai**	Ref. 7	Ref. 7	16.69	0.76	1.54	0.79	
**Shu Guang**	Ref. 7	Ref. 7	14.82	2.51	6.06	3.55	
**Shu Guang Hong Xian Ya Bian**	97.04	6.92	1.85	5.43	7.8	2.37	
**Shuang Xi Hong**	Ref. 7	Ref. 7	11.28	2.69	5.59	2.89	
**Suan Tao**	Ref. 7	Ref. 7	10.44	2.05	3.98	1.93	NC
**Tian Jin Shui Mi**	Ref. 7	Ref. 7	13.26	1.68	3.55	1.86	NC
**Tian Ren Tao**	Ref. 7	Ref. 7	4.43	1.04	1.69	0.66	NWC
**Wu Han 2**	95.32	6.27	2.76	5.55	8.07	2.52	
**Wu Hei Ji Rou Tao**	Ref. 7	Ref. 7	15.95	1.17	2.74	1.57	MLCJ
**Wu Yue Xian**	Ref. 7	Ref. 7	17.33	2.53	6.36	3.82	NC
**Wu Yue Xian Bian Gan**	Ref. 7	Ref. 7	15.25	2.48	5.75	3.27	NC
**Xi Kang Bian Tao 1**	77.25	4.48	11.43	2.72	4.66	1.94	
**Xia Miao 1**	Ref. 7	Ref. 7	15.23	1.8	4.09	2.29	NWC
**Xian Tao**	Ref. 7	Ref. 7	12.78	0.84	1.68	0.84	SC
**Xin Jiang Huang Rou**	Ref. 7	Ref. 7	9.99	1.27	2.36	1.09	NWC
**Xin Jiang Pan Tao**	Ref. 7	Ref. 7	14.72	2.65	6.33	3.68	NWC
**Xin Jiang Pan Tao 1 (pollen sterility)**	81.6	2.2	19.27	1.25	3.09	1.84	NWC
**Yan Hong**	Ref. 7	Ref. 7	10.06	3.47	6.9	3.43	
**Yang Zhou 3**	94.43	5.23	3.13	6.24	9.28	3.04	
**Ying Ge Tao**	Ref. 7	Ref. 7	12.59	3.95	8.68	4.73	SC
**Ying Zui**	84.2	2.42	16.73	2.26	5.54	3.28	MLCJ
**Yu Bai**	Ref. 7	Ref. 7	17.01	2.17	5.26	3.09	NC
**Yuan Yang Chui Zhi**	Ref. 7	Ref. 7	18.72	1.04	2.5	1.46	
**Zao Huang Pan Tao**	97.72	7.96	1.15	7.46	10.4	2.94	
**Zao Shang Hai Shui Mi**	Ref. 7	Ref. 7	2.95	6.25	9.75	3.5	MLCJ
**Zao Shu Huang Gan**	Ref. 7	Ref. 7	2.11	4.29	6.17	1.88	NWC
**Zao Shu Xia Ye Tao**	96.69	6.62	3.95	3.08	4.77	1.68	
**Zhong Hua Shou Tao**	Ref. 7	Ref. 7	3.11	1.14	1.75	0.61	NC
**Zhong You Pan Tao 2**	96.07	5.62	2.32	4.87	7.13	2.26	
**Zhong You Pan Tao 4**	97.24	6.91	1.29	5.83	8.18	2.35	
**Zhong You Tao 4**	95.66	7	2.87	5.32	7.83	2.51	
**Zhong You Tao 4 Zao Shu Ya Bian**	95.75	5.95	3.03	5.56	8.09	2.52	
**Zhong You Tao 5**	95.9	5.8	1.82	5.47	7.92	2.44	

**Zhong You Tao 5 Zao Shu Ya Bian**	96.75	5.66	2.88	5.16	7.63	2.46
**Zhong You Tao 9**	96.87	6.92	1.33	5.49	7.68	2.2
**Zhong You Tao 9 Hei Xian Bai Rou Ya Bian**	95.73	5.51	1.74	5.29	7.54	2.24
**Zhong You Tao 9 Huang Rou Ya Bian**	96.77	6.45	2.81	4.75	7.09	2.34
**Zhong You Tao 9 Wang Shu Ya Bian**	97.25	7.35	1.8	5.17	7.41	2.24
**Zhou Xing Shan Tao**	Ref. 7	Ref. 7	15.28	4.91	9.2	4.29
**Zhu Fen Chui Zhi**	Ref. 7	Ref. 7	18.21	1.17	2.77	1.6
**Average**	89.28	4.21	10.1	3.08	5.54	2.45

The codes for the six geographic regions are as follows: Northwest China (NWC), YunGui plateau (YGC), Northeast China (NEC), Northern China (NC), the middle and lower reaches of the Changjiang River (MLCJ), and Southern China (SC).

Supplementary Table 4: Validation of SNPs calling before imputation and after that using Sequenom MassARRAY platform.

	Resequencing data before imputation	Resequencing data after imputation
**Untyped**	303	57
**Consistent homozygous loci with MassARRAY**	2832	2921
**Inconsistent homozygous loci with MassARRAY**	194	193
**Consistent heterozygous loci with MassARRAY**	290	325
**Inconsistent heterozygous loci with MassARRAY**	56	74
**Total**	3675	3570
**Percent of untyped loci**	8.24%	1.60%
**Percent of consistent homozygous loci with MassARRAY**	93.59%	93.80%
**Percent of consistent heterozygous loci with MassARRAY**	83.82%	81.45%

Supplementary Table 5: Average heterozygosity rates of total imputed SNPs in the different sample groups.

Groups	Subgroups	Group capacity (n)	Heterozygosity rates [imputed] (%)
**Wild related species**	Almond	2	1.90%
	P. mira	3	2.61%
	P. davidiana	5	5.91%
	P. kansuensis	2	1.60%
	Total	12	3.61%
**Landraces**	Landraces, Ornamental	9	1.25%
	Landraces, edible	53	2.32%
	Total	62	2.17%
**Improved varieties**	Occidental	10	1.57%
	Oriental	45	2.71%
	Total	55	2.50%

In the table, the description of all accession derived from two Supplementary Table 1 in this and previous study[7].

Supplementary Table 6: The polymorphism and SNP count of total imputed SNPs in the different sample groups.

Group	Wild related species	Cultivated					
		Total	Ornamental	Edible landrace	Improved varieties		
					Total	Occidental	Oriental
**Group capacity (n)**	12	117	9	53	55	10	45
**Polymorphism *π* (10-3)**	5.74	1.83	1.47	1.86	1.55	1.38	1.55
**SNP count ($\times 10^6$)**	3.63	1.67	0.93	1.53	1.39	1.35	0.92

Differences in polymorphism indicate that peach edible landraces retain little nucleotide diversity and exhibit a clear genetic differentiation from their wild progenitor. The nucleotide diversity (π) of wild species (*P. davidiana*, *P. mira*, and *P. kansuensis*) is higher than that of peach edible landraces, highlighting the strong reduction of variability associated with domestication.

Supplementary Table 7: Association analysis of 10 qualitative traits in 129 peach accessions using different models.

Trait	GLM (no PCA)	$-Log_{10}(p)$	GLM (PCA)	$-Log_{10}(p)$	MLM	$-Log_{10}(p)$
**Flesh adhesion**	Chr4: 22696643	9.93	Chr4: 22695515	10.2	Chr4: 26142583	5.81
					Chr4: 22695515	5.33
					Chr4: 26945751	4.85
**Flesh texture**	Chr4: 22695495	18.12	Chr4: 22695495	14.22	Chr8: 2759375	6.49
					Chr1: 36970794	6.03
					Chr2: 11354439	5.53
**Flesh color around stone**	Chr6: 2183867	7.95	Chr8: 16905885	7.13	Chr8: 16795565	4.65
	Chr1: 45251328	7.83	Chr7: 12901845	6.89	Chr1: 31040363	4.62
	Chr7: 12901845	7.33	Chr6: 11954708	6.78	Chr4: 19678521	4.48
**Flower shape-DoubleF**	Chr2: 21480531	50.81	Chr2: 21480531	46.55	Chr6: 17602373	12.28
					Chr8: 4862362	12.02
					Chr2: 15719828	11.88
**Flower shape-showyF**	Chr8: 13740117	24.19	Chr8: 13740117	23.86	Chr8: 13740117	11.93
	Chr8: 16687764	23.87	Chr8: 16746058	15.62	Chr7: 21584843	8.61
	Chr8: 16746058	23.76	Chr8: 16807476	15.14		
**Fruit flesh color**	Chr1: 24820676	16.91	Chr1: 24968892	13.72	Chr1: 24968892	8.74
			Chr1: 24863116	12.98	Chr1: 24863116	8.13
**Fruit hairiness**	Chr5: 18238151	30.87	Chr5: 18238151	18.41	Chr5: 18238151	9.5
	Chr5: 17576893	27.11	Chr5: 16646429	16.01	Chr5: 16646429	9.06
	Chr5: 16646429	26.88	Chr5: 17576893	15.94	Chr7: 3304942	8.18
**Fruit shape**	Chr6: 25060196	27.13	Chr6: 25060196	26.04	Chr6: 25060196	12.46
	Chr6: 27109034	18.64	Chr6: 27109034	18.36	Chr6: 27109034	10.25
	Chr6: 25142326	15.26	Chr6: 25142326	15.31	Chr6: 25142326	9.62
**Kernel taste**	Chr2: 9680893	46	Chr2: 9820779	61.79	Chr2: 9820779	17.32
	Chr2: 9820626	41.44	Chr2: 9680893	48.37	Chr5: 12622852	16.81
	Chr4: 3179343	41.43	Chr5: 12622852	40.93		
**Non-acid fruit**	Chr5: 588359	33.68	Chr5: 588359	26.16	Chr5: 628407	10.5
	Chr5: 996915	31.16	Chr5: 996915	24.9	Chr5: 764830	10.01
	Chr5: 764830	28.18	Chr5: 541075	20.77		

In the different models, we selected only one peak association signal if it has a near physical distance with the next signal according to P value degree no more than 50 kb. And if the distance was longer than that threshold, another two signals were selected as candidate SNPs.

Supplementary Table 8: Previously reported linkage results for several qualitative traits in peach.

Traits	Linkage group	Linkage map	The genetics distance in the T×E groups (cM)	The corresponding physical location estimated through linkage analysis (Mb)	Reference
**Fruit hairiness**	5	Peach-JF-F2-2006	81.4	14.7	GDR website
**Fruit shape**	6	Peach-JF-F2-2006	72.6	24.7	
**Flesh adhesion**	4	Prunus-TE-F2	52.0	22.6	
**Flesh texture**	4	Prunus-TE-F2	52.0	22.6	
**Fruit flesh color**	1	Prunus-TE-F2	31.0	16.7	
**Flesh color around stone**	3	Prunus-TE-F2	17.0	7.7	
**Non-acid fruit**	5	Prunus-TE-F2	6.0	0.9	
**Flower shape-DoubleF**	2	Peach-NJPKV77119-F2	7.2	/	
**Flower shape-ShowyF**	8	Peach-DG-F1-2009	34.4	8.3	
**Kernel taste**	5	Prunus-TE-F2	32.9	12.4	

GDR website: www.rosaceae.org.

Supplementary Table 9: Agronomic traits of the additional 345 accessions.

Accessions	Origin	Description	Fruit hairiness	Fruit shape	Fruit flesh color	Non-acid fruit	Flower shape-DoubleF	Flower shape-ShowyF	Flesh adhesion	Flesh texture
**8501**	PRC	Edible landrace	Peach	Round	White	Acid	Single	Showy	Freestone	Melting
**8601**	PRC	Edible landrace	Peach	Round	White	Acid	Single	Showy	Clingstone	Melting
**8701**	PRC	Edible landrace	Peach	Round	White	Acid	Single	Showy	Freestone	Melting
**8801**	PRC	Edible landrace	Peach	Round	White	Acid	Single	Showy	Freestone	Melting
**124 Pan Tao**	PRC	Improved variety	Peach	Flat	White	Non-acid	Single	Showy	Clingstone	Melting
**21 Shi Ji**	PRC	Improved variety	Peach	Round	White	Non-acid	Single	Showy	Clingstone	Melting
**82-9**	PRC	Improved variety	Peach	Round	White	Acid	Single	Showy	Clingstone	Melting
**A Bu Bai Tao**	Japan	Improved variety	Peach	Round	White	Non-acid	Single	Showy	Clingstone	Melting
**Akatsuki**	Japan	Improved variety	Peach	Round	White	Non-acid	Single	Showy	Clingstone	Melting
**Amusiding**	USA	Improved variety	Peach	Round	White	Acid	Single	Showy	Clingstone	Melting
**An Nong Shui Mi**	PRC	Improved variety	Peach	Round	White	Non-acid	Single	Showy	Clingstone	Melting
**Anlong Bai Tao**	PRC	Edible landrace	Peach	Round	White	Acid	Single	Showy	Freestone	Melting
**Asama Hakuto**	Japan	Improved variety	Peach	Round	White	Non-acid	Single	Showy	Clingstone	Melting
**Baby Gold 5#**	USA	Improved variety	Peach	Round	Yellow	Acid	Single	Non-showy	Clingstone	Non-melting
**Baby Gold 7#**	USA	Improved variety	Peach	Round	Yellow	Acid	Single	Non-showy	Clingstone	Non-melting
**Baby Gold 8#**	USA	Improved variety	Peach	Round	Yellow	Acid	Single	Non-showy	Clingstone	Non-melting
**Bai Chong Ban Chui Zhi**	USA	Ornamental landrace	Peach	Round	White	Acid	Double	Showy	Clingstone	Melting
**Bai Dan Ban**	PRC	Ornamental landrace	Peach	Round	NA	NA	Single	Showy	NA	NA
**Bai Dan Ban Chui Zhi Tao**	PRC	Improved variety	Peach	Round	White	Acid	Single	NA	Freestone	Melting
**Bai He Tao**	PRC	Edible landrace	Peach	Round	White	Acid	Single	Showy	Freestone	Melting
**Bai Li Hu**	PRC	Edible landrace	Peach	Round	White	Acid	Single	Showy	Freestone	Melting
**Bai Mang Pan Tao**	PRC	Edible landrace	Peach	Flat	White	Non-acid	Single	Showy	Clingstone	Melting

**Bai Nian He**	PRC	Edible landrace	Peach	Round	White	Acid	Single	Showy	Clingstone	Non-melting
**Bai Nian Hu**	PRC	Edible landrace	Peach	Round	White	Acid	Single	Showy	Clingstone	Non-melting
**Baisha**	PRC	Edible landrace	Peach	Round	White	Non-acid	Single	Showy	Freestone	Melting
**Bao Chun**	PRC	Ornamental landrace	Peach	Round	White	Acid	Double	Showy	Clingstone	Melting
**Bei Nong 2#**	PRC	Improved variety	Peach	Round	White	Non-acid	Single	Showy	Clingstone	Melting
**Bei Nong Zao Shu**	PRC	Improved variety	Peach	Round	White	Acid	Single	Showy	Clingstone	Melting
**Beijing Wang Pan Tao**	PRC	Edible landrace	Peach	Flat	White	Non-acid	Single	Showy	Clingstone	Melting
**Bi Tao**	PRC	Ornamental landrace	Peach	Round	NA	NA	Double	NA	NA	NA
**Bi Xia Pan Tao**	PRC	Improved variety	Peach	Flat	White	Non-acid	Single	Showy	Clingstone	Melting
**Chang Sheng Pan Tao**	PRC	Edible landrace	Peach	Flat	White	Non-acid	Single	Showy	Clingstone	Melting
**Changling Zao Yu Lu**	PRC	Edible landrace	Peach	Round	White	Non-acid	Single	Showy	Freestone	Melting
**Changze Hakuho**	Japan	Improved variety	Peach	Round	White	Non-acid	Single	Showy	Clingstone	Melting
**Chen Pu Pan Tao**	PRC	Edible landrace	Peach	Flat	White	Non-acid	Single	Showy	Clingstone	Melting
**Cheng Xiang**	PRC	Improved variety	Peach	Round	Yellow	Acid	Single	Showy	Freestone	Melting
**Cheng Yan**	PRC	Improved variety	Peach	Round	Yellow	Acid	Single	Showy	Clingstone	Non-melting
**Chi Yuan Mi**	PRC	Improved variety	Peach	Round	White	Non-acid	Single	Showy	Clingstone	Melting
**Chiycmarn**	USA	Improved variety	Peach	Round	Yellow	Non-acid	Single	Showy	Clingstone	Melting
**Chun Lei**	PRC	Improved variety	Peach	Round	White	Non-acid	Single	Showy	Freestone	Melting
**Compact Elberta**	USA	Improved variety	Peach	Round	Yellow	Acid	Single	Non-showy	Freestone	Melting
**Cullinan**	USA	Improved variety	Peach	Round	Yellow	Acid	Single	Non-showy	Freestone	Melting
**Da Bai Suan**	PRC	Improved variety	Nectarine	Round	White	Acid	Single	Showy	Clingstone	Melting
**Da Hong Pao (Hubei Gucheng)**	PRC	Edible landrace	Peach	Round	White	Non-acid	Single	Showy	Freestone	Melting
**Da Jin Dan**	PRC	Edible landrace	Peach	Round	Yellow	Acid	Single	Showy	Freestone	Melting
**Da Li He Huang Rou**	PRC	Edible landrace	Peach	Round	Yellow	Acid	Single	Showy	Freestone	Melting
**Da Zhao Huang Tao**	PRC	Edible landrace	Peach	Round	Yellow	Non-acid	Single	Showy	Freestone	Melting

**Da Zhen Bao Chi Yue**	PRC	Improved variety	Peach	Round	White	Non-acid	Single	Showy	Clingstone	Melting
**Dalian 22-6**	PRC	Improved variety	Peach	Round	Yellow	Acid	Single	Showy	Clingstone	Non-melting
**Dalian 22-8**	PRC	Improved variety	Peach	Round	Yellow	Acid	Single	Showy	Clingstone	Melting
**Dan Bai Ai You**	PRC	Improved variety	Nectarine	Round	White	Non-acid	NA	NA	Clingstone	Melting
**Dan Ban Zi Tao**	PRC	Ornamental landrace	Peach	Round	White	Acid	Single	NA	Clingstone	Melting
**Dan Mo**	PRC	Improved variety	Nectarine	Round	Yellow	Non-acid	Single	Non-showy	Clingstone	Melting
**Dawangzhuang Huang Tao**	PRC	Edible landrace	Peach	Round	Yellow	Acid	Single	Showy	Clingstone	Non-melting
**Denjiulo**	Japan	Improved variety	Peach	Round	White	Acid	Single	Showy	Freestone	Melting
**Dicon**	USA	Improved variety	Peach	Round	Yellow	Acid	Single	Non-showy	Clingstone	Non-melting
**Dixired**	USA	Improved variety	Peach	Round	Yellow	Acid	Single	Non-showy	Freestone	Melting
**Dunhuang Dong Tao**	PRC	Edible landrace	Peach	Round	White	Acid	Single	Showy	Clingstone	Non-melting
**Durbin**	USA	Improved variety	Nectarine	Round	Yellow	Acid	Single	Non-showy	Freestone	Melting
**Dwarf Norman**	USA	Improved variety	Peach	Round	Yellow	Acid	Single	Showy	Freestone	Melting
**Early Red 2#**	USA	Improved variety	Nectarine	Round	Yellow	Acid	Single	Showy	Freestone	Melting
**Elberta (Showy)**	USA	Improved variety	Peach	Round	Yellow	Acid	Single	Showy	Freestone	Melting
**Er Jie Bai**	PRC	Edible landrace	Peach	Round	White	Non-acid	Single	Showy	Clingstone	Melting
**Er Zao Tao**	PRC	Edible landrace	Peach	Round	White	Acid	Single	Showy	Clingstone	Non-melting
**Fantasia**	USA	Improved variety	Nectarine	Round	Yellow	Acid	Single	Showy	Freestone	Melting
**Favolate 2#**	Italy	Improved variety	Peach	Round	Yellow	Acid	Single	Non-showy	Clingstone	Melting
**Favolate 3#**	Italy	Improved variety	Peach	Round	Yellow	Acid	Single	Showy	Freestone	Melting
**Fay Elberta**	USA	Improved variety	Peach	Round	Yellow	Acid	Single	Showy	Freestone	Melting
**Fayette**	USA	Improved variety	Peach	Round	Yellow	Acid	Single	Non-showy	Freestone	Melting
**Fei Cheng Bai Li 17#**	PRC	Edible landrace	Peach	Round	White	Non-acid	Single	Showy	Clingstone	Melting
**Fei Tao**	PRC	Ornamental landrace	Peach	Round	White	Acid	Double	NA	Clingstone	Melting
**Fen Hong Bi Tao**	PRC	Ornamental landrace	Peach	Round	NA	NA	Double	NA	NA	NA
**Fen Ling Chong**	PRC	Ornamental landrace	Peach	Round	White	Acid	Double	Non-showy	Clingstone	Melting

**Feng Bai**	PRC	Improved variety	Peach	Round	White	Acid	Single	Showy	Freestone	Melting
**Feng Huang**	PRC	Improved variety	Peach	Round	Yellow	Acid	Single	Showy	Clingstone	Non-melting
**Fenghua Pan Tao**	PRC	Edible landrace	Peach	Flat	White	Non-acid	Single	Showy	Clingstone	Melting
**Flavortop**	USA	Improved variety	Nectarine	Round	Yellow	Acid	Single	Showy	Freestone	Melting
**Flordadown**	USA	Improved variety	Peach	Round	Yellow	Acid	Single	Showy	Freestone	Melting
**Flordagold**	USA	Improved variety	Peach	Round	Yellow	Acid	Single	Showy	Freestone	Melting
**Flordaking**	USA	Improved variety	Peach	Round	Yellow	Acid	Single	Non-showy	Clingstone	Melting
**Gan Xuan 2#**	PRC	Improved variety	Peach	Round	Yellow	Acid	Single	Showy	Clingstone	Melting
**Gan Xuan 4#**	PRC	Improved variety	Peach	Round	White	Non-acid	Single	Showy	Freestone	Melting
**Gaotai 1#**	PRC	Edible landrace	Peach	Round	White	Acid	Single	Showy	Clingstone	Non-melting
**GF677**	France	Improved variety	Peach	Round	White	Acid	Single	Showy	Clingstone	Melting
**Goldcrest**	USA	Improved variety	Peach	Round	Yellow	Acid	Single	Showy	Clingstone	Melting
**Grand 1#**	USA	Improved variety	Nectarine	Round	Yellow	Acid	Single	Showy	Clingstone	Melting
**Grand 2#**	USA	Improved variety	Nectarine	Round	Yellow	Acid	Single	Showy	Freestone	Melting
**Gu Ba Hong Tian Tao**	Cuba	Improved variety	Peach	Round	White	Non-acid	Single	Showy	Clingstone	Non-melting
**Gu Cheng Chun Lei**	PRC	Edible landrace	Peach	Round	White	Non-acid	Single	Showy	Clingstone	Melting
**Gua Tao**	PRC	Edible landrace	Peach	Round	Yellow	Acid	Single	Showy	Freestone	Melting
**Guangyi Bai Hua Tao**	PRC	Edible landrace	Peach	Round	White	Non-acid	Single	Showy	Freestone	Melting
**Guangyi Wu Yue Tao**	PRC	Edible landrace	Peach	Round	White	Non-acid	Single	Showy	Clingstone	Melting
**Guizhou Shui Mi**	PRC	Edible landrace	Peach	Round	White	Non-acid	Single	Showy	Clingstone	Melting
**Ha Lu Hong Shi Sheng**	PRC	Improved variety	Peach	Round	White	Acid	Single	Non-showy	Freestone	Melting
**Hakuto**	Japan	Improved variety	Peach	Round	White	Non-acid	Single	Showy	Clingstone	Melting
**Han Lu Mi**	PRC	Edible landrace	Peach	Round	White	Non-acid	Single	Showy	Clingstone	Melting
**Hang Yu**	PRC	Improved variety	Peach	Round	White	Non-acid	Single	Showy	Clingstone	Melting
**Hangzhou Zao Shui Mi**	PRC	Improved variety	Peach	Round	White	Acid	Single	Showy	Clingstone	Melting
**Harmony**	USA	Improved variety	Peach	Round	Yellow	Acid	Single	Non-showy	Freestone	Melting

**Hatsukami**	Japan	Improved variety	Peach	Round	White	Non-acid	Single	Showy	Clingstone	Melting
**Hefei Zao Tian Tao**	PRC	Improved variety	Peach	Round	NA	NA	NA	NA	NA	NA
**Hetian Huang Rou**	PRC	Edible landrace	Peach	Round	Yellow	Acid	Single	Showy	Freestone	Melting
**Hetian Wan You Tao**	PRC	Edible landrace	Nectarine	Round	Yellow	Acid	Single	Showy	Clingstone	Melting
**Heyang You Tao**	PRC	Edible landrace	Nectarine	Round	Yellow	Acid	Single	Showy	Freestone	Melting
**Hong Gan Lu**	PRC	Improved variety	Peach	Round	White	Non-acid	Single	Showy	Clingstone	Melting
**Hong Ling Chong**	PRC	Ornamental landrace	Peach	Round	White	Acid	Double	Non-showy	Clingstone	Melting
**Hong Qing Shui**	Japan	Improved variety	Peach	Round	White	Non-acid	Single	Showy	Clingstone	Melting
**Hong Tao**	PRC	Edible landrace	Peach	Round	White	Acid	Single	Showy	Freestone	Melting
**Hong Xian Shu Guang**	PRC	Improved variety	Nectarine	Round	Yellow	Non-acid	NA	NA	Clingstone	Melting
**Hu You 004#**	PRC	Improved variety	Nectarine	Round	Yellow	Non-acid	Single	Showy	Freestone	Melting
**Huang La Tao**	PRC	Edible landrace	Peach	Round	Yellow	Acid	Single	Showy	Freestone	Melting
**Huang Li Guang**	PRC	Edible landrace	Nectarine	Round	Yellow	Acid	Single	Showy	Freestone	Melting
**Huang Nian He**	PRC	Edible landrace	Peach	Round	Yellow	Acid	Single	Showy	Clingstone	Non-melting
**Huang Yan**	PRC	Edible landrace	Peach	Round	Yellow	Acid	Single	Showy	Clingstone	Non-melting
**Hui Yu Lu**	PRC	Improved variety	Peach	Round	White	Non-acid	Single	Showy	Clingstone	Melting
**Improved Flavor Crest**	USA	Improved variety	Peach	Round	Yellow	Acid	Single	Showy	Freestone	Melting
**Ji Zui Bai**	PRC	Edible landrace	Peach	Round	White	Non-acid	Single	Showy	Freestone	Melting
**Jia Na Yan**	Japan	Improved variety	Peach	Round	White	Non-acid	Single	Showy	Clingstone	Melting
**Jiang Tao**	PRC	Ornamental landrace	Peach	Round	White	Acid	Double	NA	Clingstone	Melting
**Jiangcun 1#**	PRC	Edible landrace	Peach	Round	Yellow	Acid	Single	Showy	Freestone	Melting
**Jiangcun 4#**	PRC	Edible landrace	Peach	Round	Yellow	Acid	Single	Showy	Clingstone	Non-melting
**Jiangcun 5#**	PRC	Edible landrace	Peach	Round	Yellow	Acid	Single	Showy	Clingstone	Non-melting
**Jiaqing Pan Tao**	PRC	Edible landrace	Peach	Flat	White	Non-acid	Single	Showy	Clingstone	Melting
**Jie Tu Bai**	PRC	Improved variety	Peach	Round	White	Non-acid	Single	Showy	Freestone	Melting
**Jin Shuo**	PRC	Improved variety	Nectarine	Round	Yellow	Non-acid	Single	Showy	Clingstone	Melting

**Jin Xiu**	PRC	Improved variety	Peach	Round	Yellow	Non-acid	Single	Showy	Clingstone	Melting
**Jing Chun**	PRC	Improved variety	Peach	Round	White	Non-acid	Single	Showy	Clingstone	Melting
**Jing Mi**	PRC	Improved variety	Peach	Round	White	Non-acid	Single	Showy	Clingstone	Melting
**Jing Yan**	PRC	Improved variety	Peach	Round	White	Non-acid	Single	Showy	Clingstone	Melting
**Jingmen Tao (Mao)**	PRC	Edible landrace	Peach	Round	White	Non-acid	Single	Showy	Freestone	Melting
**Jingmen Tao (You)**	PRC	Edible landrace	Nectarine	Round	NA	NA	Single	Showy	NA	NA
**Jiu Yang Qing Tao**	PRC	Edible landrace	Peach	Round	White	Acid	Single	Showy	Freestone	Melting
**June Gold**	USA	Improved variety	Peach	Round	Yellow	Acid	Single	Showy	Freestone	Melting
**June prince**	USA	Improved variety	Nectarine	Round	Yellow	Acid	Single	Showy	Freestone	Melting
**Jushan Tong Tao**	PRC	Edible landrace	Peach	Round	White	Non-acid	Single	Showy	Freestone	Melting
**Kan Zhu Bai Tao**	Japan	Improved variety	Peach	Round	White	Non-acid	Single	Showy	Clingstone	Melting
**Kanto 14#**	Japan	Improved variety	Peach	Round	Yellow	Acid	Single	Non-showy	Clingstone	Non-melting
**Kanto 5#**	Japan	Improved variety	Peach	Round	Yellow	Acid	Single	Showy	Clingstone	Non-melting
**Kashi 2#**	PRC	Edible landrace	Peach	Round	White	Acid	Single	Showy	Freestone	Melting
**Kashi 3#**	PRC	Edible landrace	Peach	Round	White	Acid	Single	Showy	Freestone	Melting
**Kashi 4#**	PRC	Edible landrace	Peach	Round	White	Acid	Single	Showy	Freestone	Melting
**Kawanakajima Hakuto**	Japan	Improved variety	Peach	Round	White	Non-acid	Single	Showy	Clingstone	Melting
**Kogetsu**	Japan	Improved variety	Peach	Round	White	Non-acid	Single	Showy	Clingstone	Melting
**Kurakato Wase**	Japan	Improved variety	Peach	Round	White	Non-acid	Single	Showy	Clingstone	Melting
**Lai Shan Mi**	PRC	Improved variety	Peach	Round	White	Non-acid	Single	Showy	Clingstone	Melting
**Le Yuan**	PRC	Ornamental landrace	Nectarine	Round	Yellow	Acid	Single	Showy	Freestone	Melting
**Li He Pan Tao**	PRC	Edible landrace	Peach	Flat	White	Non-acid	Single	Showy	Freestone	Melting
**Lian Huang**	PRC	Improved variety	Peach	Round	Yellow	Acid	Single	Showy	Clingstone	Non-melting
**Lin Bai 10#**	PRC	Edible landrace	Peach	Round	White	Acid	Single	Showy	Clingstone	Non-melting
**Lin Bai 3#**	PRC	Edible landrace	Peach	Round	White	Non-acid	Single	Showy	Clingstone	Non-melting
**Lin Huang 1#**	PRC	Edible landrace	Peach	Round	Yellow	Acid	Single	Showy	Clingstone	Melting

**Lin Huang 9#**	PRC	Edible landrace	Peach	Round	Yellow	Acid	Single	Showy	Clingstone	Non-melting
**Lincheng Tao**	PRC	Edible landrace	Peach	Round	White	Acid	Single	Showy	Clingstone	Melting
**Liquan 54#**	PRC	Improved variety	Peach	Round	White	Non-acid	Single	Showy	Clingstone	Melting
**Liu Yue Bai**	PRC	Edible landrace	Peach	Round	White	Non-acid	Single	Showy	Freestone	Melting
**Liu Yue Kong**	PRC	Edible landrace	Peach	Round	White	Non-acid	Single	Showy	Freestone	Melting
**Long 2-4-6**	PRC	Edible landrace	Peach	Round	Yellow	Acid	Single	Showy	Clingstone	Non-melting
**Long You Pan Tao**	PRC	Edible landrace	Nectarine	Flat	Yellow	Acid	Single	Showy	Clingstone	Melting
**Longhua Shui Mi**	PRC	Edible landrace	Peach	Round	White	Non-acid	Single	Showy	Clingstone	Melting
**Lu Xiang**	PRC	Improved variety	Peach	Round	Yellow	Acid	Single	Showy	Freestone	Melting
**Lulin Shui Mi**	PRC	Edible landrace	Peach	Round	White	Non-acid	Single	Showy	Freestone	Melting
**Mai Xiang**	PRC	Improved variety	Peach	Round	White	Acid	Single	Showy	Clingstone	Melting
**Man Tian Hong**	PRC	Ornamental landrace	Peach	Round	White	Non-acid	Double	Showy	Clingstone	Melting
**Maoshan Gong Tao**	PRC	Edible landrace	Peach	Round	White	Non-acid	NA	Showy	Clingstone	Melting
**Maravilha**	USA	Improved variety	Peach	Round	White	Acid	Single	Showy	Freestone	Melting
**Marigold**	USA	Improved variety	Peach	Round	Yellow	Acid	Single	Showy	Freestone	Melting
**Matsumori**	Japan	Improved variety	Peach	Round	White	Non-acid	Single	Showy	Clingstone	Melting
**May Grand**	USA	Improved variety	Nectarine	Round	Yellow	Acid	Single	Showy	Freestone	Melting
**Mayglo**	USA	Improved variety	Nectarine	Round	Yellow	Acid	Single	Showy	Clingstone	Melting
**Mcneely**	USA	Improved variety	Peach	Round	Yellow	Acid	Single	Non-showy	Freestone	Melting
**Mei Guo Pan Tao**	PRC	Improved variety	Peach	Flat	White	Non-acid	Single	Showy	Clingstone	Melting
**Mei Xiang**	PRC	Improved variety	Peach	Round	White	Non-acid	Single	Showy	Clingstone	Melting
**Mi Yang Shan**	PRC	Edible landrace	Peach	Round	White	Non-acid	Single	Showy	Freestone	Melting
**Moyu 8#**	PRC	Edible landrace	Peach	Round	Yellow	Acid	Single	Showy	Clingstone	Melting
**Nan Fang Zao Hong**	PRC	Improved variety	Nectarine	Round	White	Non-acid	Single	Showy	Clingstone	Melting
**Nanshan 1#**	PRC	Edible landrace	Peach	Round	Yellow	Non-acid	Single	Showy	Clingstone	Non-melting
**Nemaguard**	USA	Improved variety	Peach	Round	White	Acid	Single	Showy	Clingstone	Melting

**NJ271**	USA	Improved variety	Peach	Round	Yellow	Non-acid	Double	NA	Freestone	Melting
**NJC105**	USA	Improved variety	Peach	Round	Yellow	Acid	Single	Non-showy	Clingstone	Non-melting
**NJC47**	USA	Improved variety	Peach	Round	Yellow	Acid	Single	Non-showy	Clingstone	Non-melting
**NJF10**	USA	Improved variety	Peach	Flat	Yellow	Acid	Single	Showy	Freestone	Melting
**NJF7**	USA	Improved variety	Peach	Flat	White	Non-acid	Single	Showy	Clingstone	Melting
**Norman**	USA	Improved variety	Peach	Round	Yellow	Acid	Single	Showy	Freestone	Melting
**Nunome Wase**	Japan	Improved variety	Peach	Round	White	Non-acid	Single	Showy	Freestone	Melting
**Okayama 11#**	Japan	Improved variety	Peach	Round	White	Acid	Single	Showy	Clingstone	Melting
**Okayama 3#**	Japan	Improved variety	Peach	Round	White	Non-acid	Single	Showy	Clingstone	Melting
**Okayama Wase**	Japan	Improved variety	Peach	Round	White	Non-acid	Single	Showy	Freestone	Melting
**Okinawa**	USA	Improved variety	Peach	Round	White	Acid	Single	Showy	Freestone	Melting
**Pan Tao Huang Hou**	PRC	Improved variety	Peach	Flat	White	Non-acid	Single	Showy	Clingstone	Melting
**Pan Tao Wang**	PRC	Improved variety	Peach	Flat	White	Non-acid	Single	Showy	Clingstone	Melting
**Ping Ding Mao Pan Tao**	PRC	Edible landrace	Peach	Flat	White	Acid	Single	Showy	Freestone	Melting
**Ping Yong Tao**	PRC	Edible landrace	Peach	Round	White	Acid	Single	Showy	Freestone	Melting
**Qi Tao**	PRC	Edible landrace	Peach	Round	White	Acid	Single	Showy	Clingstone	Melting
**Qian Nian Hong**	PRC	Improved variety	Nectarine	Round	Yellow	Non-acid	Single	Showy	Clingstone	Melting
**Qin Guang**	PRC	Improved variety	Nectarine	Round	White	Non-acid	Single	Showy	Freestone	Melting
**Qin Guang 2#**	PRC	Improved variety	Nectarine	Round	White	Non-acid	Single	Showy	Freestone	Melting
**Qin Wang**	PRC	Improved variety	Peach	Round	White	Non-acid	Single	Showy	Clingstone	Melting
**Qinan 2#**	PRC	Improved variety	Peach	Round	White	Non-acid	Single	Showy	Clingstone	Melting
**Qing Feng**	PRC	Improved variety	Peach	Round	White	Non-acid	Single	Showy	Clingstone	Melting
**Qing Mao Zi Bai Hua**	PRC	Edible landrace	Peach	Round	White	Non-acid	Single	Showy	Freestone	Melting
**Qingzhou Hong Pi Mi Tao**	PRC	Edible landrace	Peach	Round	White	Non-acid	Single	Showy	Freestone	Melting
**Qinling Dong Tao**	PRC	Edible landrace	Peach	Round	White	Acid	Single	Showy	Freestone	Melting
**Qiu Bai Tao**	PRC	Edible landrace	Peach	Round	White	Acid	Single	Showy	Clingstone	Melting

**Qiu Xiang**	PRC	Improved variety	Peach	Round	White	Non-acid	Single	Showy	Clingstone	Melting
**Qiu Xiang Mi**	PRC	Improved variety	Peach	Round	White	Non-acid	Single	Showy	Clingstone	Melting
**Red Diamond**	USA	Improved variety	Nectarine	Round	Yellow	Acid	Single	Non-showy	Clingstone	Melting
**Regina**	USA	Improved variety	Peach	Round	Yellow	Acid	Single	Non-showy	Freestone	Melting
**Reliance**	USA	Improved variety	Peach	Round	Yellow	Acid	Single	Non-showy	Freestone	Melting
**Ren Mian Tao**	PRC	Ornamental landrace	Peach	Round	White	Acid	Double	NA	Freestone	Melting
**Ri Ben Hong Tian Tao**	Japan	Improved variety	Peach	Round	White	Non-acid	Single	Showy	Freestone	Melting
**Robin**	USA	Improved variety	Peach	Round	White	Non-acid	Single	Showy	Freestone	Melting
**Rose prince**	USA	Improved variety	Nectarine	Round	White	Acid	Single	Showy	Freestone	Melting
**Rui Guang 18#**	PRC	Improved variety	Nectarine	Round	Yellow	Non-acid	Single	Showy	Clingstone	Melting
**Rui Guang 19#**	PRC	Improved variety	Nectarine	Round	White	Non-acid	Single	Showy	Freestone	Melting
**Rui Pan 2#**	PRC	Improved variety	Peach	Flat	White	Non-acid	Single	Showy	Clingstone	Melting
**Rui Pan 4#**	PRC	Improved variety	Peach	Flat	White	Non-acid	Single	Showy	Clingstone	Melting
**Rutgers Redleaf**	USA	Improved variety	Peach	Round	White	Acid	Single	Showy	Freestone	Melting
**Saotome**	Japan	Improved variety	Peach	Round	White	Non-acid	Single	Showy	Clingstone	Melting
**Saturn**	USA	Improved variety	Peach	Flat	White	Non-acid	Single	Showy	Freestone	Melting
**S-B**	PRC	Edible landrace	Peach	Round	White	Acid	Single	Showy	Clingstone	Melting
**September free**	USA	Improved variety	Nectarine	Round	Yellow	Acid	Single	Showy	Freestone	Melting
**Sha Hong Tao**	PRC	Edible landrace	Peach	Round	White	Non-acid	Single	Showy	Clingstone	Melting
**Sha Ji 2#**	PRC	Improved variety	Peach	Round	White	Non-acid	Single	Showy	Clingstone	Melting
**Shangshan Da Yu Lu**	PRC	Edible landrace	Peach	Round	White	Non-acid	Single	Showy	Clingstone	Melting
**Shenzhou Bai Mi**	PRC	Edible landrace	Peach	Round	White	Non-acid	Single	Showy	Clingstone	Melting
**Shenzhou Li He Shui Mi**	PRC	Edible landrace	Peach	Round	White	Non-acid	Single	Showy	Freestone	Melting
**Shi Sheng 3#**	Japan	Improved variety	Peach	Round	White	Non-acid	Single	Showy	Freestone	Melting
**Shi Tao**	PRC	Edible landrace	Peach	Round	White	Acid	Single	Showy	Freestone	Melting
**Shou Fen**	PRC	Ornamental landrace	Peach	Round	White	Acid	Double	Showy	Clingstone	Melting

**Shuho**	Japan	Improved variety	Nectarine	Round	Yellow	Acid	Single	Showy	Clingstone	Non-melting
**Silver Gem**	USA	Improved variety	Nectarine	Round	White	Acid	Single	Showy	Freestone	Melting
**Sirlo**	USA	Improved variety	Nectarine	Round	Yellow	Acid	Single	Showy	Freestone	Melting
**Southern pearl**	USA	Improved variety	Peach	Round	White	Acid	Single	Showy	Freestone	Melting
**Spring baby**	USA	Improved variety	Peach	Round	White	Acid	Single	Showy	Clingstone	Non-melting
**Spring Crest**	USA	Improved variety	Peach	Round	Yellow	Acid	NA	NA	Clingstone	Melting
**Spring gold**	USA	Improved variety	Peach	Round	Yellow	Acid	Single	Showy	Freestone	Melting
**Spring prince**	USA	Improved variety	Peach	Round	Yellow	Acid	Single	Showy	Clingstone	Melting
**Springtime**	USA	Improved variety	Peach	Round	White	Acid	Single	Non-showy	Clingstone	Melting
**Su Lian Pan Tao**	Soviet	Improved variety	Peach	Flat	White	Non-acid	Single	Showy	Freestone	Melting
**Suan Li Guang**	PRC	Edible landrace	Nectarine	Round	White	Acid	Single	Showy	Freestone	Melting
**Summergrand**	USA	Improved variety	Nectarine	Round	Yellow	Acid	Single	Showy	Freestone	Melting
**Summerset**	USA	Improved variety	Peach	Round	Yellow	Acid	Single	Non-showy	Freestone	Melting
**Sunago Wase**	Japan	Improved variety	Peach	Round	White	Non-acid	Single	Showy	Clingstone	Melting
**Sunblaze**	USA	Improved variety	Nectarine	Round	Yellow	Acid	Single	Non-showy	Freestone	Melting
**Sundollar**	USA	Improved variety	Nectarine	Round	Yellow	Acid	Single	Showy	Clingstone	Melting
**Sunraycer**	USA	Improved variety	Nectarine	Round	Yellow	Acid	Single	Non-showy	Freestone	Melting
**Sunsplash**	USA	Improved variety	Nectarine	Round	Yellow	Acid	Single	Showy	Clingstone	Melting
**Tachibana Wase**	Japan	Improved variety	Peach	Round	White	Acid	Single	Showy	Freestone	Melting
**Tai Bai**	PRC	Edible landrace	Peach	Round	White	Non-acid	Single	Showy	Clingstone	Melting
**Taiyuan Shui Mi**	PRC	Edible landrace	Peach	Round	White	Non-acid	Single	Showy	Clingstone	Melting
**Tan Chun**	PRC	Ornamental landrace	Peach	Round	NA	NA	Double	Showy	NA	NA
**Tian Hong**	South Korea	Improved variety	Nectarine	Round	Yellow	Acid	Single	Showy	Freestone	Melting
**Tian Li Guang**	PRC	Edible landrace	Nectarine	Round	White	Acid	Single	Showy	Freestone	Melting
**Tie 4-1**	PRC	Edible landrace	Peach	Round	Yellow	Acid	Single	Showy	Freestone	Melting
**Trinmph**	USA	Improved variety	Peach	Round	Yellow	Acid	Single	Showy	Freestone	Melting

**Tsukuba 85#**	Japan	Improved variety	Peach	Round	White	Non-acid	Single	Showy	Clingstone	Melting
**Tsukuba 86#**	Japan	Improved variety	Peach	Round	White	Non-acid	Single	Showy	Clingstone	Melting
**Tu-2**	PRC	Edible landrace	Peach	Round	Yellow	Acid	Single	Showy	Clingstone	Non-melting
**Tugou 1#**	PRC	Edible landrace	Peach	Round	Yellow	Acid	Single	Showy	Clingstone	Non-melting
**Wan Bai Hua**	PRC	Improved variety	Peach	Round	White	Non-acid	Single	Showy	Clingstone	Melting
**Wan Bai Mi**	PRC	Improved variety	Peach	Round	White	Non-acid	Single	Showy	Clingstone	Melting
**Wan Huang Jin**	Japan	Improved variety	Peach	Round	Yellow	Non-acid	Single	Showy	Clingstone	Melting
**Wan Pan Tao**	PRC	Edible landrace	Peach	Flat	White	Non-acid	Single	Showy	Clingstone	Melting
**Wangmo Xiao Mi Tao**	PRC	Edible landrace	Peach	Round	White	Non-acid	Single	Showy	Freestone	Melting
**Wanzhou Huang Tao**	PRC	Edible landrace	Peach	Round	NA	NA	NA	NA	NA	NA
**Wanzhou Xiang Tao**	PRC	Edible landrace	Peach	Round	NA	NA	Single	NA	NA	NA
**Wasesimizu**	Japan	Improved variety	Peach	Round	White	Acid	Single	Showy	Clingstone	Melting
**Wei Yang 2#**	PRC	Improved variety	Peach	Round	White	Non-acid	Single	Showy	Clingstone	Melting
**Weinberger**	Italy	Improved variety	Nectarine	Round	Yellow	Acid	Single	Showy	Freestone	Melting
**Wenzhou Shui Mi**	PRC	Edible landrace	Peach	Round	White	Non-acid	Single	Showy	Clingstone	Melting
**Wu Bao Tao**	PRC	Ornamental landrace	Peach	Round	NA	NA	Double	NA	NA	NA
**Wu Da Tao**	PRC	Edible landrace	Peach	Round	White	Acid	Single	Showy	Freestone	Melting
**Wu Yue Bai**	PRC	Edible landrace	Peach	Round	White	Non-acid	Single	Showy	Clingstone	Melting
**Wu Yue Jin**	PRC	Improved variety	Peach	Round	Yellow	Non-acid	Single	Showy	Clingstone	Melting
**Wuhan Da Hong Pao**	PRC	Edible landrace	Peach	Round	White	Non-acid	Single	Showy	Freestone	Melting
**Xi Jiao 1#**	PRC	Edible landrace	Peach	Round	Yellow	Acid	Single	Showy	Clingstone	Non-melting
**Xi Jiao 2#**	PRC	Edible landrace	Peach	Round	Yellow	Non-acid	Single	Showy	Clingstone	Non-melting
**Xi Jiao 3#**	PRC	Edible landrace	Peach	Round	White	Acid	Single	Showy	Freestone	Melting
**Xi Mei 1#**	PRC	Improved variety	Peach	Round	White	Acid	Single	Showy	Freestone	Melting
**Xi Mei 2#**	PRC	Improved variety	Peach	Round	White	Acid	Single	Showy	Freestone	Melting
**Xi Nong 18#**	PRC	Improved variety	Peach	Round	White	Acid	Single	Showy	Freestone	Melting

**Xi Nong 19#**	PRC	Improved variety	Peach	Round	White	Acid	Single	Showy	Freestone	Melting
**Xi Nong Shui Mi**	PRC	Improved variety	Peach	Round	White	Acid	Single	Showy	Clingstone	Melting
**Xi Nong Xia Mi**	PRC	Improved variety	Peach	Round	White	Non-acid	Single	Showy	Clingstone	Melting
**Xi Nong Zao Mi**	PRC	Improved variety	Peach	Round	White	Acid	Single	Showy	Clingstone	Melting
**Xi Wei Gold**	South Korea	Improved variety	Peach	Round	Yellow	Non-acid	Single	Showy	Clingstone	Melting
**Xia Hui 1#**	PRC	Improved variety	Peach	Round	White	Non-acid	Single	Showy	Clingstone	Melting
**Xiang Jiao Tao**	PRC	Edible landrace	Peach	Round	Yellow	Non-acid	Single	Showy	Freestone	Melting
**Xiao Bai Tao**	PRC	Edible landrace	Peach	Round	White	Non-acid	Single	Showy	Freestone	Melting
**Xiao Hong Hua**	PRC	Edible landrace	Peach	Round	White	Non-acid	Single	Showy	Clingstone	Melting
**Xiao Jin Dan**	PRC	Edible landrace	Peach	Round	White	Non-acid	Single	Showy	Freestone	Melting
**Xiboliya C**	Canada	Improved variety	Peach	Round	White	Acid	Single	Showy	Freestone	Melting
**Xin Bai Hua**	PRC	Improved variety	Peach	Round	White	Non-acid	Single	Showy	Clingstone	Melting
**Xin Hong Zao Pan Tao**	PRC	Improved variety	Peach	Flat	White	Acid	Single	Showy	Freestone	Melting
**Xiu Yu**	PRC	Improved variety	Nectarine	Round	White	Non-acid	Single	Showy	Clingstone	Melting
**Xuancheng Tian Tao**	PRC	Improved variety	Peach	Round	White	Non-acid	Single	Showy	Freestone	Melting
**Yan Guang**	PRC	Improved variety	Nectarine	Round	White	Non-acid	Single	Non-showy	Clingstone	Melting
**Yang Tao**	PRC	Edible landrace	Peach	Round	White	Non-acid	Single	Showy	Clingstone	Melting
**Yangquan Rou Tao**	PRC	Edible landrace	Peach	Round	White	Non-acid	Single	Showy	Clingstone	Melting
**Yangzhou 40#**	PRC	Improved variety	Peach	Round	Yellow	Non-acid	Single	Showy	Clingstone	Melting
**Yexian Dong Tao**	PRC	Edible landrace	Peach	Round	White	Non-acid	Single	Showy	Freestone	Melting
**Yexian Huang Rou Tao**	PRC	Edible landrace	Peach	Round	Yellow	Acid	Single	Showy	Clingstone	Melting
**Yi Xian Hong**	PRC	Edible landrace	Peach	Round	White	Non-acid	Single	Showy	Freestone	Melting
**Yilixian Huang Rou Tao**	PRC	Edible landrace	Peach	Round	Yellow	Acid	Single	Showy	Freestone	Melting
**Ying Chun**	PRC	Ornamental landrace	Peach	Round	White	Acid	Double	Showy	Clingstone	Melting
**You Ming Bai Tao**	South Korea	Improved variety	Peach	Round	White	Non-acid	Single	Showy	Clingstone	Melting
**Yu Hua Lu**	PRC	Improved variety	Peach	Round	White	Non-acid	Single	Showy	Freestone	Melting

**Yu Lu**	PRC	Improved variety	Peach	Round	White	Non-acid	Single	Showy	Clingstone	Melting
**Yu Lu Pan Tao**	PRC	Edible landrace	Peach	Flat	White	Non-acid	Single	Showy	Clingstone	Melting
**Yu Tian**	PRC	Improved variety	Peach	Round	White	Non-acid	Single	Showy	Freestone	Melting
**Yuan Chun**	PRC	Ornamental landrace	Peach	Round	White	Non-acid	Double	Showy	Clingstone	Melting
**Yuan Dong Bai Tao**	PRC	Improved variety	Peach	Round	White	Non-acid	Single	Showy	Freestone	Melting
**Yun Shu 2#**	PRC	Improved variety	Peach	Round	White	Non-acid	Single	Showy	Clingstone	Melting
**Zao Bai Feng**	PRC	Improved variety	Peach	Round	White	Non-acid	Single	Showy	Freestone	Melting
**Zao Chun Tao**	PRC	Edible landrace	Peach	Round	White	Acid	Single	Showy	Clingstone	Melting
**Zao Feng**	PRC	Improved variety	Peach	Round	White	Non-acid	Single	Showy	Clingstone	Melting
**Zao Hong Xia**	PRC	Improved variety	Nectarine	Round	White	Non-acid	Single	Showy	Clingstone	Melting
**Zao Hong Yan**	PRC	Improved variety	Nectarine	Round	White	Non-acid	Single	Showy	Freestone	Melting
**Zao Hong Zhu**	PRC	Improved variety	Nectarine	Round	White	Non-acid	Single	Non-showy	Clingstone	Melting
**Zao Huang Jin**	PRC	Edible landrace	Peach	Round	Yellow	Acid	Single	Showy	Freestone	Melting
**Zao Jiu Bao**	PRC	Improved variety	Peach	Round	White	Non-acid	Single	Showy	Freestone	Melting
**Zao Kui**	PRC	Improved variety	Peach	Round	White	Non-acid	Single	Showy	Clingstone	Melting
**Zao Kui Mi**	PRC	Improved variety	Peach	Flat	White	Non-acid	Single	Showy	Clingstone	Melting
**Zao Lu Pan Tao**	PRC	Improved variety	Peach	Flat	White	Non-acid	Single	Showy	Clingstone	Melting
**Zao Mei**	PRC	Improved variety	Peach	Round	White	Non-acid	Single	Showy	Clingstone	Melting
**Zao Tian Tao**	PRC	Improved variety	Peach	Round	White	Non-acid	Single	Showy	Clingstone	Melting
**Zao Xia Lu**	PRC	Improved variety	Peach	Round	White	Non-acid	Single	Showy	Freestone	Melting
**Zao Yan**	PRC	Improved variety	Peach	Round	White	Non-acid	Single	Showy	Freestone	Melting
**Zhang Bai 2#**	PRC	Edible landrace	Peach	Round	White	Acid	Single	Showy	Clingstone	Non-melting
**Zhang Bai 5#**	PRC	Edible landrace	Peach	Round	White	Non-acid	Single	Showy	Clingstone	Non-melting
**Zhang Bai Gan**	PRC	Edible landrace	Peach	Round	White	Acid	Single	Showy	Clingstone	Non-melting
**Zhang Huang 9#**	PRC	Edible landrace	Peach	Round	Yellow	Acid	Single	Showy	Clingstone	Non-melting
**Zhao Hui**	PRC	Improved variety	Peach	Round	White	Non-acid	Single	Showy	Clingstone	Melting

**Zhen Zhu Hong**	PRC	Improved variety	Nectarine	Round	White	Acid	Single	Non-showy	Clingstone	Melting
**Zheng Huang 2#**	PRC	Improved variety	Peach	Round	Yellow	Acid	Single	Showy	Clingstone	Non-melting
**Zheng Huang 3#**	PRC	Improved variety	Peach	Round	Yellow	Acid	Single	Showy	Clingstone	Non-melting
**Zheng Huang 4#**	PRC	Improved variety	Peach	Round	Yellow	Acid	Single	Showy	Clingstone	Non-melting
**Zhong You 7#**	PRC	Improved variety	Nectarine	Round	NA	NA	Single	Non-showy	NA	NA
**Zhong You 8#**	PRC	Improved variety	Nectarine	Round	Yellow	Non-acid	Single	Non-showy	Clingstone	Melting
**Zhong You Pan Tao 1#**	PRC	Improved variety	Nectarine	Flat	White	Non-acid	Single	Non-showy	Clingstone	Melting
**Zhu Bo 2#**	Japan	Improved variety	Peach	Round	White	Acid	Single	Showy	Freestone	Melting
**Zhu Bo 3#**	Japan	Improved variety	Peach	Round	White	Acid	Single	Showy	Freestone	Melting
**Zhu Bo 88#**	Japan	Improved variety	Peach	Round	White	Non-acid	Single	Non-showy	Clingstone	Melting
**Zhu Bo 89#**	Japan	Improved variety	Peach	Round	White	Acid	Single	Showy	Clingstone	Melting
**Zi Rou Tao**	PRC	Improved variety	Nectarine	Round	Yellow	Acid	Single	Showy	Freestone	Melting

Supplementary Table 10: Significant association results for selected SNPs using Sequenom MassARRAY data in 345 peach accessions.

Trait	GLM	Associated SNP using GLM (no PCA), GLM (PCA) or MLM model	$-Log_{10}(p)$
**Flower shape-DoubleF**	Chr2: 21480531	GLM (no PCA), GLM (PCA)	20.64
	Chr6: 17602373	MLM	31.95
	Chr8: 4862362	MLM	8.97
	Chr2: 15719828	MLM	NA
**Flower shape-showyF**	Chr8: 13740117	GLM (no PCA), GLM (PCA), MLM	121.47
	Chr8: 16687764	GLM (no PCA)	2.3
	Chr8: 16746058	GLM (no PCA), GLM (PCA)	1.5
	Chr8: 16807476	GLM (PCA)	2.64
	Chr7: 21584843	MLM	7.57
**Fruit flesh color**	Chr1: 24820676	GLM (no PCA)	21.24
	Chr1: 24968892	GLM (PCA), MLM	25.43
	Chr1: 24863116	GLM (PCA), MLM	21.51
**Fruit hairiness**	Chr5: 18238151	GLM (no PCA), GLM (PCA), MLM	32.04
	Chr5: 17576893	GLM (no PCA), GLM (PCA)	121.92
	Chr5: 16646429	GLM (no PCA), GLM (PCA), MLM	98.34
	Chr7: 3304942	MLM	5.23
**Fruit shape**	Chr6: 25060196	GLM (no PCA), GLM (PCA), MLM	182.72
	Chr6: 27109034	GLM (no PCA), GLM (PCA), MLM	73.10
	Chr6: 25142326	GLM (no PCA), GLM (PCA), MLM	48.86
**Non-acid fruit**	Chr5: 588359	GLM, GLM (PCA)	51.57
	Chr5: 628407	MLM	62.47
	Chr5: 996915	GLM (no PCA), GLM (PCA)	70.75
	Chr5: 764830	GLM (no PCA), MLM	58.02
	Chr5: 541075	GLM (PCA)	89.42

Supplementary Table 11: Significant association results for soluble solid content and fruit weight in 129 peach accessions using MLM model.

Trait	Loci	Years	Chr.	Position	-log10 (p)
**Fruit weight**	**FW-2007-1**	**2007**	1	43205141	6.26
	**FW-2007-2**	**2007**	2	16609769	6.74
	**FW-2007-3**	**2007**	3	6338918	5.97
	**FW-2007-4**	**2007**	4	10990743	7.14
	**FW-2007-5**	**2007**	5	4949487	6.30
	**FW-2007-6**	**2007**	5	13892312	5.96
	**FW-2007-7**	**2007**	5	15928967	6.21
	**FW-2007-8**	**2007**	6	13747142	6.07
	**FW-2007-9**	**2007**	7	14069835	6.17
	**FW-2007-10**	**2007**	8	168831	7.00
	**FW-2010-1**	**2010**	1	12392427	4.55
	**FW-2010-2**	**2010**	1	39241272	4.21
	**FW-2010-3**	**2010**	1	14050831	4.15
	**FW-2010-4**	**2010**	1	5879950	4.11
	**FW-2010-5**	**2010**	2	10388301	4.65
	**FW-2010-6**	**2010**	2	16602967	4.68
	**FW-2010-7**	**2010**	2	20032011	4.11
	**FW-2010-8**	**2010**	2	23069073	4.01
	**FW-2010-9**	**2010**	2	24271176	4.01
	**FW-2010-10**	**2010**	3	6281041	4.60
	**FW-2010-11**	**2010**	3	7768702	4.23
	**FW-2010-12**	**2010**	4	8634341	4.54
	**FW-2010-13**	**2010**	4	10888626	4.97
	**FW-2010-14**	**2010**	4	13194190	4.63
	**FW-2010-15**	**2010**	4	17625270	4.53
	**FW-2010-16**	**2010**	4	20500702	4.23
	**FW-2010-17**	**2010**	4	23281118	4.95
	**FW-2010-18**	**2010**	4	29527539	4.38
	**FW-2010-19**	**2010**	5	660217	4.45
	**FW-2010-20**	**2010**	5	9432987	4.77
	**FW-2010-21**	**2010**	6	20400960	4.10
	**FW-2010-22**	**2010**	7	19291656	4.16
	**FW-2010-23**	**2010**	8	18961633	4.09
**Soluble solid content**	**SSC-2007-1**	**2007**	1	15691351	4.02
	**SSC-2007-2**	**2007**	1	18146487	4.42
	**SSC-2007-3**	**2007**	1	20779945	4.42
	**SSC-2007-4**	**2007**	1	24175076	4.14
	**SSC-2007-5**	**2007**	2	20036936	4.13
	**SSC-2007-6**	**2007**	2	23174414	4.99
	**SSC-2007-7**	**2007**	2	25426115	4.32

	**SSC-2007-8**	**2007**	3	688273	4.17
	**SSC-2007-9**	**2007**	3	9101540	4.04
	**SSC-2007-10**	**2007**	3	11022073	4.14
	**SSC-2007-11**	**2007**	4	1968175	4.69
	**SSC-2007-12**	**2007**	4	5478311	4.14
	**SSC-2007-13**	**2007**	4	8873368	4.55
	**SSC-2007-14**	**2007**	4	17057020	4.14
	**SSC-2007-15**	**2007**	4	24462378	4.14
	**SSC-2007-16**	**2007**	6	19782877	4.05
	**SSC-2007-17**	**2007**	6	24180924	4.60
	**SSC-2007-18**	**2007**	7	597258	5.03
	**SSC-2007-19**	**2007**	7	2463182	4.12
	**SSC-2007-20**	**2007**	8	15176331	4.14
	**SSC-2007-21**	**2007**	8	18584579	4.03
	**SSC-2010-1**	**2010**	1	10477624	4.31
	**SSC-2010-2**	**2010**	7	19910443	4.23
	**SSC-2010-3**	**2010**	8	13010893	4.74

Supplementary Table 12: Domestication and improvement sweep regions according to the polymorphisms identified in contiguous 50-kb windows across peach chromosomes.

Scaffold	Start point (bp)	End point (bp)	Sweep regions		Scaffold	Start point (bp)	End point (bp)	Sweep regions	
**1**	200001	250000	Domestication		**4**	8850001	8900000	Domestication	
**1**	700001	850000		Improvement	**4**	11000001	11050000		Improvement
**1**	1000001	1050000	Domestication		**4**	11500001	11550000	Domestication	
**1**	2450001	2500000		Improvement	**4**	11750001	11850000	Domestication	
**1**	5600001	5650000	Domestication		**4**	15600001	15650000		Improvement
**1**	5950001	6100000		Improvement	**4**	15850001	15900000		Improvement
**1**	6400001	6450000		Improvement	**4**	16800001	16850000	Domestication	
**1**	6800001	6850000		Improvement	**4**	17750001	17800000	Domestication	
**1**	7200001	7250000	Domestication		**4**	18050001	18150000		Improvement
**1**	7950001	8000000	Domestication		**4**	18200001	18250000		Improvement
**1**	8050001	8100000	Domestication		**4**	20300001	20450000	Domestication	
**1**	8150001	8200000		Improvement	**5**	100001	200000		Improvement
**1**	9100001	9150000	Domestication		**5**	1600001	1650000		Improvement
**1**	9150001	9200000	Domestication	Improvement	**5**	1800001	2000000		Improvement
**1**	9450001	9500000		Improvement	**5**	2300001	2500000		Improvement
**1**	10400001	10450000	Domestication		**5**	2650001	2700000		Improvement
**1**	10900001	10950000		Improvement	**5**	2850001	2900000		Improvement
**1**	11000001	11050000		Improvement	**5**	3350001	3400000	Domestication	
**1**	14400001	14700000		Improvement	**5**	3850001	4050000		Improvement
**1**	14750001	14800000	Domestication		**5**	4150001	4200000		Improvement
**1**	16300001	16350000	Domestication		**5**	5400001	5450000	Domestication	
**1**	16800001	16850000		Improvement	**5**	7750001	7800000	Domestication	Improvement
**1**	17350001	17750000	Domestication		**5**	7850001	7950000		Improvement
**1**	17750001	17800000	Domestication	Improvement	**5**	8000001	8050000	Domestication	
**1**	17800001	18500000	Domestication		**5**	8800001	8850000	Domestication	
**1**	22000001	22050000	Domestication		**5**	10750001	10850000		Improvement
**1**	22650001	22800000	Domestication		**5**	12150001	12350000	Domestication	
**1**	23750001	23800000		Improvement	**5**	12450001	12500000	Domestication	
**1**	24100001	24150000	Domestication		**5**	12550001	12600000	Domestication	
**1**	25150001	25200000		Improvement	**5**	12700001	12800000		Improvement
**1**	25250001	25450000		Improvement	**5**	13150001	13200000	Domestication	
**1**	28800001	28850000	Domestication		**5**	13250001	13300000		Improvement
**1**	28950001	29000000	Domestication		**5**	13750001	13800000	Domestication	
**1**	29050001	29200000	Domestication		**5**	14650001	14700000	Domestication	
**1**	30450001	30500000		Improvement	**5**	14800001	14850000	Domestication	
**1**	30650001	30700000	Domestication		**5**	14950001	15200000		Improvement
**1**	30750001	30800000	Domestication		**5**	15250001	15350000	Domestication	Improvement
**1**	31350001	31400000	Domestication		**5**	15550001	15600000	Domestication	Improvement

**1**	31550001	31650000		Improvement	**5**	15700001	15800000	Domestication	
**1**	32950001	33000000	Domestication		**5**	15800001	15850000		Improvement
**1**	33200001	33300000	Domestication		**5**	15900001	16050000		Improvement
**1**	34400001	34450000	Domestication		**5**	16100001	16200000	Domestication	
**1**	35000001	35050000	Domestication		**5**	16200001	16250000		Improvement
**1**	35850001	35900000	Domestication		**5**	16300001	16400000		Improvement
**1**	36000001	36050000	Domestication		**5**	16400001	16450000	Domestication	Improvement
**1**	36150001	36250000	Domestication		**5**	16800001	16850000		Improvement
**1**	37650001	37700000	Domestication		**5**	17000001	17100000		Improvement
**1**	38150001	38350000		Improvement	**5**	17150001	17200000	Domestication	
**1**	38350001	38400000	Domestication	Improvement	**5**	17300001	17350000		Improvement
**1**	38450001	38500000		Improvement	**5**	17400001	17500000	Domestication	
**1**	39000001	39050000		Improvement	**5**	17550001	17600000		Improvement
**1**	39450001	39550000		Improvement	**5**	17600001	17750000	Domestication	
**1**	39650001	39900000		Improvement	**5**	17750001	17800000	Domestication	Improvement
**1**	39900001	39950000	Domestication	Improvement	**5**	17800001	17850000	Domestication	
**1**	39950001	40000000		Improvement	**5**	17900001	18000000		Improvement
**1**	40850001	40950000	Domestication		**5**	18050001	18100000	Domestication	Improvement
**1**	41050001	41100000	Domestication		**5**	18100001	18200000	Domestication	
**1**	41100001	41150000	Domestication	Improvement	**5**	18250001	18300000	Domestication	Improvement
**1**	41150001	41500000	Domestication		**5**	18300001	18350000	Domestication	
**1**	41500001	41550000	Domestication	Improvement	**5**	18350001	18450000	Domestication	Improvement
**1**	41550001	41700000	Domestication		**6**	2750001	2800000	Domestication	
**1**	45000001	45050000	Domestication		**6**	4050001	4100000	Domestication	
**1**	45300001	45350000	Domestication		**6**	4150001	4250000	Domestication	
**1**	46000001	46050000	Domestication		**6**	4850001	4900000		Improvement
**1**	46150001	46200000	Domestication		**6**	4950001	5000000	Domestication	
**1**	46350001	46400000	Domestication		**6**	5250001	5300000		Improvement
**2**	100001	150000	Domestication		**6**	5600001	5900000		Improvement
**2**	200001	250000	Domestication		**6**	6050001	6200000		Improvement
**2**	8950001	9050000	Domestication		**6**	6650001	6700000	Domestication	
**2**	9700001	9800000		Improvement	**6**	6850001	6900000		Improvement
**2**	10250001	10300000		Improvement	**6**	7400001	7450000	Domestication	
**2**	10400001	10550000		Improvement	**6**	8800001	8850000	Domestication	Improvement
**2**	11250001	11300000		Improvement	**6**	8850001	8900000		Improvement
**2**	11600001	11700000		Improvement	**6**	9700001	9900000		Improvement
**2**	12350001	12400000		Improvement	**6**	12800001	12850000	Domestication	
**2**	15100001	15150000		Improvement	**6**	15900001	15950000		Improvement
**2**	15450001	15500000	Domestication		**6**	16100001	16250000		Improvement
**2**	15750001	15800000	Domestication		**6**	16300001	16400000		Improvement
**2**	16600001	16900000		Improvement	**6**	16750001	16800000	Domestication	
**2**	16950001	17000000	Domestication		**6**	19850001	19900000		Improvement
**2**	22800001	22850000	Domestication		**6**	21250001	21400000	Domestication	
**2**	23600001	23700000	Domestication		**6**	24550001	24600000	Domestication	Improvement

**2**	23750001	24000000		Improvement	**6**	28250001	28300000	Domestication	
**2**	24050001	24100000	Domestication		**6**	28300001	28350000	Domestication	Improvement
**2**	24150001	24200000	Domestication		**6**	28400001	28450000		Improvement
**2**	24250001	24300000		Improvement	**6**	28550001	28900000	Domestication	
**2**	25100001	25150000	Domestication		**7**	3150001	3200000		Improvement
**2**	25700001	25750000	Domestication		**7**	8800001	8850000	Domestication	
**2**	26000001	26050000		Improvement	**7**	8950001	9050000		Improvement
**3**	1500001	1550000	Domestication	Improvement	**7**	12450001	12500000	Domestication	
**3**	2100001	2150000	Domestication		**7**	13500001	13600000	Domestication	
**3**	2850001	2900000	Domestication		**7**	13750001	13900000		Improvement
**3**	4550001	4600000	Domestication		**7**	14950001	15050000	Domestication	
**3**	4650001	4700000	Domestication		**7**	15400001	15450000	Domestication	
**3**	5200001	5300000		Improvement	**7**	15600001	15650000	Domestication	
**3**	5300001	5350000	Domestication		**7**	17050001	17100000	Domestication	
**3**	7500001	7550000	Domestication		**7**	17150001	17200000	Domestication	
**3**	8050001	8100000	Domestication		**7**	17300001	17350000	Domestication	
**3**	8550001	8600000	Domestication		**7**	17750001	17800000		Improvement
**3**	8650001	8750000	Domestication		**7**	17850001	17900000	Domestication	
**3**	8850001	8900000	Domestication		**7**	18900001	18950000	Domestication	
**3**	9100001	9200000	Domestication		**7**	19050001	19200000		Improvement
**3**	12450001	12500000	Domestication		**7**	19700001	19750000	Domestication	
**3**	12600001	12650000	Domestication		**7**	20450001	20500000	Domestication	
**3**	13000001	13050000		Improvement	**7**	20650001	20700000		Improvement
**3**	13100001	13150000		Improvement	**7**	20700001	20750000	Domestication	Improvement
**3**	13450001	13500000		Improvement	**7**	21850001	21950000		Improvement
**3**	13550001	13650000		Improvement	**7**	22000001	22050000	Domestication	Improvement
**3**	13850001	13900000	Domestication		**7**	22050001	22100000	Domestication	
**3**	14700001	14750000		Improvement	**7**	22100001	22250000		Improvement
**3**	16000001	16050000	Domestication		**8**	1050001	1100000		Improvement
**3**	16550001	16600000	Domestication		**8**	1900001	1950000		Improvement
**3**	18700001	18750000		Improvement	**8**	2600001	2700000		Improvement
**3**	18950001	19050000		Improvement	**8**	2750001	2900000		Improvement
**3**	19050001	19250000	Domestication		**8**	3350001	3400000		Improvement
**3**	19300001	19350000		Improvement	**8**	8200001	8250000		Improvement
**3**	19400001	19450000		Improvement	**8**	10450001	10500000		Improvement
**3**	20600001	20650000	Domestication		**8**	11700001	11750000	Domestication	
**3**	21200001	21300000		Improvement	**8**	12550001	12650000	Domestication	
**3**	21400001	21500000	Domestication		**8**	12850001	12900000	Domestication	
**4**	1650001	1950000		Improvement	**8**	13150001	13200000		Improvement
**4**	2000001	2300000		Improvement	**8**	14150001	14200000	Domestication	
**4**	3350001	3400000		Improvement	**8**	14450001	14500000	Domestication	
**4**	3700001	3750000	Domestication		**8**	14600001	14650000	Domestication	
**4**	6800001	6850000	Domestication		**8**	14700001	14750000	Domestication	
**4**	7600001	7650000	Domestication		**8**	15700001	15750000	Domestication	

**4**	7700001	7850000	Domestication	**8**	18300001	18400000	Domestication
**4**	7950001	8150000	Domestication	**8**	19800001	19850000	Domestication
**4**	8350001	8500000	Domestication	**8**	21550001	21800000	Domestication

191

Supplementary Table 13: Candidate genes identified within the overlapping regions of domestication and improvement sweep and fruit weight or soluble solid content association analysis.

	Biological process	Peach gene ID
**The overlapping region of improvement sweep and fruit weight association siganls**	Nitrogen compound metabolic process	ppa007510m, ppa002331m, ppa021345m, ppa012648m
	Catabolic process	ppa011112m, ppa004836m, ppa005453m
	Biosynthetic process	ppa007021m, ppa007023m, ppa021345m, ppa007510m, ppa014786m
	Macromolecule metabolic process	ppa002234m, ppa002331m, ppa004836m, ppa005453m, ppa011112m, ppa012641m, ppa014805m, ppa021345m, ppa024695m, ppa024757m
	Cellular metabolic process	ppa002234m, ppa002331m, ppa007021m, ppa007023m, ppa007510m, ppa011112m, ppa012641m, ppa012648m, ppa014786m, ppa014805m, ppa021345m, ppa024695m, ppa024757m
	Primary metabolic process	ppa002234m, ppa002331m, ppa002727m, ppa002732m, ppa004378m, ppa004836m, ppa005453m, ppa007021m, ppa007023m, ppa007510m, ppa009638m, ppa009641m, ppa009655m, ppa011112m, ppa012641m, ppa012648m, ppa014786m, ppa014805m, ppa021345m, ppa024695m, ppa024757m
	Oxidation reduction	ppa003219m, ppa004476m, ppa004495m, ppa004579m, ppa004947m, ppa014786m, ppa020113m, ppa026124m
	Cellular process	ppa000110m, ppa000314m, ppa001323m, ppa002234m, ppa002331m, ppa003476m, ppa004575m, ppa007021m, ppa007023m, ppa007510m, ppa011112m, ppa012641m, ppa012648m, ppa014786m, ppa014805m, ppa015394m, ppa015909m, ppa021345m, ppa021446m, ppa022308m, ppa024695m, ppa024757m, ppa027119m
	Cellular component organization	ppa000314m, ppa021446m, ppa022308m
	Pigmentation	ppa000110m, ppa004575m, ppa015394m
	Cellular component biogenesis	ppa003476m
	Response to stimulus	ppa002331m
	Localization	ppa027119m, ppa027119m
	Establishment of localization	ppa027119m, ppa015909m
	Biological regulation	ppa000110m, ppa004575m, ppa015394m, ppa021277m
	No terms assigned in this category	ppa000012m, ppa001708m, ppa002229m, ppa002451m, ppa003022m, ppa004401m, ppa004482m, ppa004671m, ppa005479m, ppa006158m, ppa006283m, ppa006728m, ppa010383m, ppa010699m, ppa010702m, ppa010703m, ppa011409m, ppa012185m, ppa012267m, ppa012348m, ppa012942m, ppa013169m, ppa013815m, ppa015556m, ppa015814m, ppa016961m, ppa017589m, ppa018285m, ppa018314m, ppa018337m, ppa018787m, ppa019578m, ppa019735m, ppa020139m, ppa020189m, ppa020346m, ppa021643m, ppa021765m, ppa022615m, ppa022622m, ppa023020m, ppa024416m, ppa025152m, ppa025538m, ppa025838m, ppa025855m, ppa026236m, ppa026854m, ppb021969m
**The overlapping**	Catabolic process	ppb021028m, ppa015079m, ppa015434m, ppa026388m, ppb019796m

**region of domestication sweep and soluble solid content association siganls**	Nitrogen compound metabolic process	ppa003404m, ppa024202m
	Biosynthetic process	ppa024202m, ppa014441m, ppa003251m, ppa003404m
	Macromolecule metabolic process	ppa001625m, ppa003651m, ppb021028m, ppa024202m, ppa014441m, ppa006217m, ppa015079m, ppa015434m, ppa026388m, ppb019796m
	Cellular metabolic process	ppa001625m, ppa003651m, ppa024202m, ppa014441m, ppa006217m, ppa003251m, ppa003404m
	Primary metabolic process	ppa001625m, ppa003651m, ppb021028m, ppa006217m, ppa014441m, ppa015079m, ppa015434m, ppa026388m, ppb019796m, ppb021028m, ppa003404m, ppa003251m
	Oxidation reduction	ppa007597m, ppa007600m, ppa014989m, ppa024861m, ppa002564m, ppa003251m
	Cellular process	ppa001625m, ppa003651m, ppa024202m, ppa014441m, ppa006217m, ppa003251m, ppa003404m
	Localization	ppa010017m
	Establishment of localization	ppa010017m
	No terms assigned in this category	ppa008169m, ppa009145m, ppa011448m, ppa018288m, ppa021260m, ppa021752m, ppa023132m, ppa023632m, ppa023728m, ppa025543m, ppb016181m, ppb022060m, ppb023061m, ppa001962m, ppa005637m, ppa007362m, ppa009249m, ppa010706m, ppa011619m, ppa014427m, ppa015483m, ppa015866m, ppa016251m, ppa018013m, ppa018087m, ppa018183m, ppa020146m, ppa020360m, ppa021260m, ppa021786m, ppa022180m, ppa024597m, ppa025357m, ppb019814m, ppa025795m, ppa000835m, ppa005874m, ppa005999m, ppa008497m, ppa010592m, ppa010704m, ppa017699m, ppa018881m

Supplementary Table 14: Agronomic variation of the soluble sugar content and titratable acid trait in 2014 in edible landraces and improved varieties of the 129 resequencing accessions.

Trait		Ornamental landrace	Edible landrace	Improved variety		
				Total	Occidental	Oriental
	Accession number	9	53	55	10	45
**Soluble sugar content (%)**	Minimum value	10.03	8.10	7.55	8.28	7.55
	Maximal value	14.71	15.55	20.28	12.50	20.28
	Average	12.57	11.39	11.32	10.43	11.66
	Standard deviation	2.24	1.72	2.51	1.37	2.78
**Titratable acid (%)**	Minimum value	0.57	0.16	0.14	0.51	0.14
	Maximal value	1.08	1.18	0.96	0.96	0.64
	Average	0.85	0.45	0.38	0.68	0.27
	Standard deviation	0.23	0.27	0.24	0.16	0.14

198 **Supplementary Table 15: Whether is the regions with abnormal low r^2 across**
199 **whole peach genome under domestication.**

**Scaffold**	**Physical location**		**LD**		**D'/r^2**	**Under domestication**
	Start point	End point	D'	r^2		
**1**	45300001	45350000	0.0981	0.0012	81.75	Yes
**4**	7600001	7650000	1	0.0173	57.80347	Yes
**2**	9000001	9050000	0.07421	0.00154	48.18831	Yes
**8**	13100001	13150000	0.8541	0.02204	38.75227	
**4**	14150001	14200000	0.60896	0.01818	33.49615	
**5**	18050001	18100000	0.55588	0.0234	23.75556	Yes
**5**	15600001	15650000	0.54655	0.02504	21.82708	
**8**	21450001	21500000	0.84545	0.04381	19.29811	
**1**	38850001	38900000	0.77029	0.04148	18.57015	
**6**	28600001	28650000	0.73592	0.04457	16.51155	Yes
**1**	38900001	38950000	0.59021	0.03697	15.96457	
**1**	38800001	38850000	0.58177	0.03651	15.93454	
**4**	24100001	24150000	0.73771	0.04673	15.78665	
**4**	2050001	2100000	0.91905	0.06189	14.84973	
**4**	16300001	16350000	0.54691	0.03787	14.44177	
**1**	16200001	16250000	0.58402	0.0432	13.51898	
**4**	17100001	17150000	0.60835	0.04538	13.40569	
**5**	12250001	12300000	0.48893	0.03689	13.25373	Yes
**7**	15050001	15100000	0.49892	0.03798	13.13639	
**3**	12850001	12900000	0.56495	0.04369	12.93088	
**2**	8200001	8250000	0.49557	0.03955	12.53021	
**1**	41300001	41350000	0.48248	0.03852	12.52544	Yes

Supplementary Table 16: Correlation between the geographic origin and the hierarchical cluster analysis of the landrace groups.

Geographic population	Total	Dendrogram based genetic similarity						χ^2
		Subgroup1	Subgroup2	Subgroup3	Subgroup4	Subgroup5	Subgroup6	
**SC**	2	2	0	0	0	0	0	χ^2=64.15
**MLCJ**	12	2	0	5	1	4	0	P<0.01
**NC**	11	0	1	7	0	3	0	$\chi^2_{0.01,\ 25}$ =
**NWC**	11	0	0	0	11	0	0	44.31
**NEC**	1	0	0	1	0	0	0	
**YGC**	3	0	0	0	0	1	2	
**Total**	40	4	1	13	12	8	2	

Supplementary Notes 1

Polymorphism change during peach domestication. Different with previous study which focused on genomic polymorphism, we mainly analyzed the heterozygosity rates of all accessions (Supplementary Table 3) and on how they differed between species groups to evaluate genomic variation. Within the wild species group, heterozygosity rates increased from almond (1.90%) to *P. mira* (2.61%), and increasing abruptly in *P. davidiana* (5.91%). As peach evolution continued, heterozygosity rates decreased, as can be determined from the values obtained for *P. kansuensis* (1.60%) and ornamental landraces (can be reckoned as wild *P. persica*, 1.25%) (Supplementary Table 5). This general decrease in heterozygosity rates during evolution might be due to peach's self-pollination system[1]. The abnormally high diversity found in *P. davidiana*, on the other hand, is in accordance with its wider geographical, climatic, and cultural range compared to the other *Prunus* species. Among the cultivated groups, the heterozygosity rate observed in the 55 improved varieties was higher than that of the 53 edible landraces (2.50% vs. 2.32%), suggesting that improved varieties tend to be more heterozygous than edible landraces (Supplementary Table 5). However, improved varieties had a lower number of SNPs than edible landraces (Supplementary Table 6). This trend might be explained by the outcross of most Chinese, European, and North American cultivars with a few founder parents, such as 'Chinese cling'[2]. And among improved varieties, we found a small reduction in the π value of all 10 occidental varieties (1.38×10^{-3}) in contrast to the other 45 oriental ones (1.55×10^{-3}) (Supplementary Table 6). The low polymorphism observed in occidental varieties is also in agreement with their low heterozygosity rate (1.57%) compared to oriental peach (2.71%) (Supplementary Table 5). However, the unbalanced sample number (10 vs. 45) might be contributing to the observed differences between these two groups. The relationships depicted here were consistent with the conclusions of the International Peach Genome Initiative[3], which suggested that a bottleneck existed between eastern and western varieties, related to the recent introduction of peach to the United States (16th to 19th century).

The evolution of different geographic subgroups in landraces. The edible landraces group were divided into six subgroups according to phylogenetic analysis (Supplementary Fig. 24).

Simultaneously, these landraces also can be clustered into six populations based on geographic origin (Supplementary Table 3): Northwest China (NWC), YunGui plateau (YGC), Northeast China (NEC), Northern China (NC), the middle and lower reaches of the Changjiang River (MLCJ), and Southern China (SC). Here we found that subgroup 1 should be considered as an admixed population as it contains accessions from the SC and MLCJ populations. Subgroup 2 consisted of only one accession from the NC population whereas subgroups 3 and 5 were mostly composed of NC and MLCJ populations' accessions. Subgroup 4 contained 12 accessions, all of which originated from the NWC population, and subgroup 6 included two accessions from the YGC population (Supplementary Fig. 3c). The correlation between the two classifications was analysed in all accessions, and the highly significant association ($\chi2 = 64.15 \geq \chi_2^{0.\ 01,\ 25} = 44.31$) between the hierarchical clusters (subgroups) based on geographic regions and the phylogenetic groups was found, revealing that peach evolution corresponded mirrored anthropogenic activity across geographic regions (Supplementary Table 16).

Meanwhile, although it was widely accepted that peach domestication originated in NWC[4] or NC[5], a recent archaeological study proposed that the lower Yangzi River valley (equivalent to MLCJ in this study) was the primary site of peach selection and domestication[6]. According to Supplementary Fig. 3a, subgroup 1 comprises the most primitive edible landraces, which is in agreement with the archaeological data[6] because SC and MLCJ populations are within the Yangtze and Pearl river valleys, respectively, being areas of ancient human activity. As domestication can be regarded as the first stage of plant breeding, and geographical differences among cultivated species are likely to result from migration and subsequent differential selection by local farmers[7], peach distribution in the SC and MLCJ regions may reflect the influence of human activity on the evolution of peach.

Supplementary References

1. Wang, Z. H. & Zhuang, E. J. *China Fruit Monograph-Peach Flora*. Beijing: *China Forestry Press* (2001).
2. Infante, R., Martínez, G. P. & Predieri, S. Quality oriented fruit breeding: peach [*Prunus persica* (L.) batsch]. *J. Food Agric. Environ.* **6**, 342-356 (2008).
3. The International Peach Genome Initiative. The high-quality draft genome of peach (*Prunus persica*) identifies unique patterns of genetic diversity, domestication and genome evolution. *Nat. Genet.* **45**, 487-494 (2013).
4. Faust, M. & Timon, B. *Origin and dissemination of peach*. *Horticultural Reviews* **17**, 331-379 (1995).
5. Li, H. L. The domestication of plants in China: Ecogeographical considerations. In: Keightley DN, editor.*The Origins of Chinese Civilization*. Berkeley, CA: *University of California Press*. pp. 21–63 (1988).
6. Zheng, Y. F., Crawford, G. W. & Chen, X. G. Archaeological evidence for peach (*Prunus persica*) cultivation and domestication in China. *PLoS One* **9**, e106595 (2014).
7. Gross, B. L. & Olsen, K. M. Genetic perspectives on crop domestication. Trends in *Plant Sci.* **15**, 529-537 (2010).
